# Northern Spotted Owl (*Strix occidentalis caurina*) Genome: Divergence with the Barred Owl (*Strix varia*) and Characterization of Light-Associated Genes

**DOI:** 10.1093/gbe/evx158

**Published:** 2017-08-23

**Authors:** Zachary R. Hanna, James B. Henderson, Jeffrey D. Wall, Christopher A. Emerling, Jérôme Fuchs, Charles Runckel, David P. Mindell, Rauri C. K. Bowie, Joseph L. DeRisi, John P. Dumbacher

**Affiliations:** 1 Museum of Vertebrate Zoology, University of California, Berkeley, California, USA; 2 Department of Integrative Biology, University of California, Berkeley, California, USA; 3 Department of Ornithology & Mammalogy, California Academy of Sciences, San Francisco, California, USA; 4 Center for Comparative Genomics, California Academy of Sciences, San Francisco, California, USA; 5 Institute for Human Genetics, University of California, San Francisco, California, USA; 6 UMR 7205 Institut de Systématique, Evolution, Biodiversité, CNRS, MNHN, UPMC, EPHE, Sorbonne Universités, Muséum National d’Histoire Naturelle, Paris, France; 7 Department of Biochemistry and Biophysics, University of California, San Francisco, California, USA; 8 Howard Hughes Medical Institute, Bethesda, Maryland, USA; 9 Runckel & Associates, Portland, Oregon, USA

**Keywords:** nuclear genome, bird, Strigidae, Strigiformes, Aves

## Abstract

We report here the assembly of a northern spotted owl (*Strix occidentalis caurina*) genome. We generated Illumina paired-end sequence data at 90× coverage using nine libraries with insert lengths ranging from ∼250 to 9,600 nt and read lengths from 100 to 375 nt. The genome assembly is comprised of 8,108 scaffolds totaling 1.26 × 10^9^ nt in length with an N50 length of 3.98 × 10^6^ nt. We calculated the genome-wide fixation index (*F*_ST_) of *S. o. caurina* with the closely related barred owl (*Strix varia*) as 0.819. We examined 19 genes that encode proteins with light-dependent functions in our genome assembly as well as in that of the barn owl (*Tyto alba*). We present genomic evidence for loss of three of these in *S. o. caurina* and four in *T. alba*. We suggest that most light-associated gene functions have been maintained in owls and their loss has not proceeded to the same extent as in other dim-light-adapted vertebrates.

## Introduction

The spotted owl (*Strix occidentalis*) is a large, charismatic inhabitant of dense forests whose range extends along the Pacific coast of North America from southwestern British Columbia to southern California and eastward into the southwest desert states and Mexico. The northern spotted owl subspecies, *S. o. caurina*, inhabits the Pacific Northwest portion of the *S. occidentalis* range from British Columbia south along the west coast to the Golden Gate strait, California. The US Fish and Wildlife Service listed *S. o. caurina* as “threatened” under the Endangered Species Act (ESA) in 1990 (Thomas etal. 1990) and the owl has been the subject of much ecological research and economic tension. Since its listing under the ESA, populations have continued to decline (Forsman etal. 1996, 2011; Dugger etal. 2015; Davis etal. 2016) despite the increased level of protection. Although it is not considered a “model species” by most researchers, there is a considerable amount of demographic and ecological data available for this species (Courtney etal. 2004), especially in comparison with other owls, which tend to be less studied than diurnal birds.

Spotted owl conservation efforts often focus on genetic challenges, including those relating to small population sizes and inbreeding, relationships to other population segments, and potential interbreeding with congeners (Barrowclough etal. 1999, 2005, 2011; Haig etal. 2001, 2004). A complete genome assembly could provide many useful tools for conservation geneticists, including independent estimates of effective population size (*N*_e_), tools for identifying and developing genetic markers such as single nucleotide polymorphisms and microsatellites, and data that can provide direct and relatively accurate measures of interbreeding.

The congeneric barred owl (*Strix varia*), formerly native to North America east of the Rocky Mountains (Mazur and James 2000), has invaded western North America in the last 50–75 years and, from British Columbia to southern California, has become broadly sympatric with the spotted owl in the last 50 years (Taylor and Forsman 1976; Livezey 2009a, 2009b) and likely poses a threat to the survival of the northern spotted owl (Forsman etal. 2011; Wiens etal. 2014; Dugger etal. 2015; Diller etal. 2016). In addition to competing for western forest habitat, barred and spotted owls interact at the genetic level as they can hybridize and successfully backcross (Haig etal. 2004; Kelly and Forsman 2004; Funk etal. 2007). Much of our motivation to assemble the northern spotted owl genome was to provide a resource to aid those studying the genetics of this owl and related taxa. Thus, we included analyses of thegenome of a barred owl from eastern North America as a baseline comparison to the spotted owl. We compared genome-derived estimates of *N*_e_ from both species and calculated *F*_ST_ between them.

Access to high-coverage, relatively complete genomes also allows researchers to address questions that, without this resource, are inaccessible or difficult to answer. For example, previous work has suggested that owls have evolved an atypical avian visual system with high numbers of dim-light-adaptive rod photoreceptors (Fite 1973; Bowmaker and Martin 1978) and a diminished capacity for color vision (Bowmaker and Martin 1978; Wu etal. 2016). Whole genome sequencing can establish what mutation(s) or genomic rearrangements resulted in their reduced color vision and, with multiple genomes, one may test whether such mutations are lineage-specific or inherited from a common ancestor. The genome assembly of the barn owl (*Tyto alba*; Aves: Tytonidae) was available and allowed us to test owl-lineage-based hypotheses, but it was one of the lower-coverage, less complete of the available avian genome assemblies (Zhang, Li B, Li C, etal. 2014). A complete spotted owl genome, in addition to providing whole genome data for a representative of Strigidae, the other of the two families of owls, could also enable a definitive search for genes involved in nocturnal visual adaptations and a better understanding of the processes of mutation that lead to such adaptations.

## Materials and Methods

### Genome Sample

We collected blood from a captive adult northern spotted owl (*S. o. caurina*) at WildCare rehabilitation facility in San Rafael, California. The captive owl, named Sequoia and referred to assuch hereafter, patient card No. 849, was admitted to WildCare on 5 June 2005 as an abandoned nestling found in Larkspur, Marin County, California (CAS:ORN:98821; table1). We chose to sequence the genome of this individual as *S. occidentalis* is known to hybridize with *S. varia* (Haig etal. 2004; Kelly and Forsman 2004; Funk etal. 2007) and we wanted to ensure that we were sequencing the genome of a nonhybrid, nonintrogressed individual. The first Marin County *S. varia* detections occurred in 2003 and researchers estimated a population size of only three individuals by 2005 (Jennings etal. 2011). First generation hybrid individuals are phenotypically diagnosable with intermediate plumage characteristics (Hamer etal. 1994). Thus, if Sequoia had any *S. varia* genetic material, it would likely have been a first generation hybrid and easily diagnosable as such. No plumage or behavioral features, such as vocalizations, suggested that it was a hybrid individual.

**Table 1:** Specimen Data

Specimen	County	State	Country	Date	Specimen Institution
CAS:ORN:98821	Marin County	CA	United States	26 Jun 2005	California Academy of Sciences
CNHM<USA-OH>:ORNITH	Hamilton County	OH	United States	29 Nov 2010	Cincinnati Museum Center

Note.—Information regarding the *S. o. caurina* and *S. varia* individuals from which we obtained genomic sequences for this study including the county, state, country, and date of collection for each specimen as well as the specimen code and institution where each specimen is archived.

### DNA Isolation

For genomic DNA libraries that required very high molecular weight DNA, we isolated DNA by using the precipitation method provided by the Gentra Puregene Kit (Qiagen, Netherlands) and following the manufacturer‘s protocol. We also isolated DNA using a column-based method, the DNeasy Blood & Tissue Kit (Qiagen, Netherlands), and used this DNA for those libraries where very high molecular weight was not essential. We assessed the quality and concentration of all isolated DNA using a Nanodrop 2000c spectrophotometer (Thermo Fisher Scientific, USA), 2100 BioAnalyzer (Agilent Technologies, USA), Qubit 2.0 Flurometer (Invitrogen, USA), and by running the DNA on a 1% agarose gel. We determined that the resulting DNA from both methods had high molecular weight with most of the DNA comprising fragments >50,000 nucleotides (nt) in length.

### Illumina Data

We obtained paired-end Illumina data from nine whole-genome libraries constructed using a variety of methods with a range of average insert lengths from 247 to 9,615 nt. In our library construction we utilized a range of DNA shearing methods including enzyme-based, ultrasonication, and hydrodynamic forces using a Hydroshear DNA Shearing Device (GeneMachines, USA). We amplified all but one of the libraries using polymerase chain reaction (PCR) and sequenced them with read lengths from 100 to 375 nt (see supplementary table S1, Supplementary Material online; supplementary section 1.1–1.8, Supplementary Material online).

### Trimming, Merging, Error-Correction

We trimmed the Nextera mate-pair data using the software NxTrim version 0.2.3-alpha (O’Connell 2014; O’Connell etal. 2015) (supplementary section 1.9.1, Supplementary Material online) in order to classify reads of mate pair libraries as true mate pair reads, paired-end reads, or singleton reads. We then removed adapters and low quality bases separately for the resulting mate-pair sequences, paired-end sequences, and singleton sequences using Trimmomatic version 0.32 (Bolger etal. 2014) (supplementary section 1.9.2, Supplementary Material online). We also used Trimmomatic to remove adapters from all non-mate-pair libraries (supplementary section 1.10.1, Supplementary Material online). In order to test how various trimming methods affected the assembly outcome, we trimmed to different thresholds for some of our preliminary assemblies by changing the Trimmomatic version 0.32 (Bolger etal. 2014) average quality score parameters. We did not apply the error-correction process to reads trimmed to a stringent quality threshold. For some preliminary assemblies, we performed adapter and quality trimming, but did not merge overlapping paired-end reads (supplementary section 1.13, Supplementary Material online). However, since substantial portions of the paired-end reads from all of the libraries, except the Nextera700 bp library, were overlapping, for the sequences that we used to generate our final assembly we joined overlapping paired reads using BBMap version 34.00 (Bushnell 2014) (supplementary section 1.10.2, Supplementary Material online). We then performed quality trimming on the non-mate-pair library data using Trimmomatic version 0.32 (Bolger etal. 2014) (supplementary section 1.10.3, Supplementary Material online). Since we trimmed using the relatively lenient threshold of trimming the read when the average quality over 4 bp dropped below quality score (Phred) 17, we next used the k-mer-based error corrector in the SOAPdenovo2 toolkit, SOAPec version 2.01 (Luo etal. 2012), to correct sequence errors (supplementary section 1.11, Supplementary Material online). For any read that became unpaired due to the loss of the paired read we separately subjected it to the same adapter, quality trimming, and error-correcting steps as the reads that remained paired (supplementary section 1.12, Supplementary Material online).

### Genome Size

In order to estimate the *S. occidentalis* nuclear genome size from our Illumina data, we ran Preqc (Simpson 2014) with the paired-end sequences from the Nextera700 bp data set (supplementary section 1.14, Supplementary Material online).

### Assembly

We assembled the *S. occidentalis* genome using SOAPdenovo2 version 2.04 (Luo etal. 2012). In order to determine the optimal assembly parameter options, we performed numerous trial runs experimenting with different k-mer values and parameters. We utilized the insert size estimated in the output of trial assemblies to refine our estimation of the insert sizes for our libraries and used these refined values as input into subsequent assembly configuration files (supplementary table S1, Supplementary Material online). After optimizing the SOAPdenovo2 assembly options, we generated fourteen further preliminary assemblies to test how using differently filtered versions and subsets of our Illumina sequence data affected the assembly outcome. We examined how the assembly was affected by trimming our data to multiple quality thresholds, using or not applying error correction, not merging or merging our overlapping paired-end data, assembling with different k-mers, using or not using singleton data, and dropping certain libraries (supplementary table S2, Supplementary Material online). We used dupchk (Henderson and Hanna 2016a) to check for sequence duplication in each sequenced library and found an elevated level of duplication in the Hydroshear library data, so we excluded all sequences from this library from several assemblies (supplementary section 1.15, Supplementary Material online).

### Preliminary Assembly Assessment

In order to compare our preliminary assemblies, we removed contiguous sequences (contigs) or scaffolds less than or equal to 300 nt with the intent of removing any unassembled reads from the assembly. We calculated the contig and scaffold N50 as well as the number of scaffolds in various length classes using scafN50 (Henderson and Hanna 2016d). We calculated the total length of the assembly, the percentage of “N” characters in the assembly that represent sequence gaps between contiguous sequences joined by paired-end or mate-pair data (% N’s), and the total number of scaffolds using scafSeqContigInfo (Henderson and Hanna 2016e). We were conservative in the calculation of these metrics and separated scaffolds into contigs at each N in the sequence. We then used CEGMA version 2.5 (Parra etal. 2007) to annotate a set of highly conserved eukaryotic genes (CEGs) in our assembly and thereby obtain an assessment of the quality and completeness of each assembly (supplementary section 1.16, Supplementary Material online).

We found it useful to assess the genome assembly‘s continuity and completeness at each stage of the assembly process. We searched for CEGs using CEGMA to evaluate our earlier assemblies. However, at this time, one of the CEGMA tool authors recommends that researchers use BUSCO in place of CEGMA (Bradnam 2015). Since we used CEGMA to evaluate our earliest assemblies, we continued to use CEGMA for continuity. We ran BUSCO on our final assembly and the results suggested similar completeness as those of CEGMA.

### Determination of Final Assembly

We examined multiple statistics in choosing our final assembly. We valued high contig and scaffold N50 values, low % N’s in the sequence, a low total number of scaffolds, larger numbers of scaffolds longer than 1 mega nucleotide (Mnt), and completeness as reflected in the number of conserved genes found by the CEGMA pipeline. We decided that the assembly that had the best statistics across these categories was assembly 4 (table 2) and proceeded forward with this assembly.

**Table 2:** Metrics of Preliminary Assemblies

Assembly	contig N50 (nt)	scaffold N50 (nt)	Total Length of Assembly (Gnt)	Ns (%)	Total Number of Scaffolds	Number Of Scaffolds > 1 Mnt In Length	Partial CEGs Found by CEGMA	Complete CEGs Found by CEGMA
1	9,499	3,869,235	1.275	4.77	51,843	292	231	205
2	12,096	3,522,724	1.274	4.40	48,264	295	233	205
3	10,425	4,007,375	1.272	4.88	47,075	0	226	200
4*	**13,983**	**3,919,460**	**1.275**	**4.26**	**47,900**	**303**	**235**	**221**
5	10,315	4,164,870	1.272	4.45	46,146	287	232	206
6	9,142	3,780,867	1.275	4.86	51,615	296	230	202
7	9,802	3,478,271	1.274	4.42	54,240	327	233	209
8	12,650	3,665,028	1.271	4.18	43,092	313	231	204
9	12,006	3,587,241	1.271	4.66	44,939	307	226	201
10	12,487	3,586,666	1.271	4.26	44,345	314	232	204
11	14,651	3,917,141	1.276	4.26	50,636	293	234	217
12	14,627	3,728,521	1.276	4.28	50,349	305	234	219
13	14,672	3,917,121	1.276	4.26	50,129	293	234	217
14	13,967	3,431,044	1.300	4.50	127,384	318	238	218

Note.—Various continuity and completeness summary statistics for our preliminary assemblies. We removed contigs/scaffolds <300 nt in order to remove unassembled reads from the assemblies before calculating these statistics. We defined contigs with the very restrictive parameter that each N split a scaffold into a separate contig. “Partial CEGs found by CEGMA” refers to the number of gene sequences found by CEGMA in the assembly in at least partial completeness out of 248 total CEGs. An asterisk and bolded font mark the preliminary assembly that we chose to use as the basis for the final assembly.

We filled gaps in the assembly using the gap closing tool in the SOAPdenovo2 toolkit, GapCloser version 1.12-r6 (Luo etal. 2012). The gap-closed assembly contained many sequences under 1,000 nt in length, a substantial portion of which appeared to be unassembled reads. We used ScaffSplitN50s (Henderson and Hanna 2016c) to compare statistics describing the continuity of the assembly after removing contigs/scaffolds of lengths 300, 500, and 1,000 nt as well as when using N blocks of lengths 1, 5, 10, 15, 20, and 25 to separate contigs within scaffolds. We decided to remove all contigs and scaffolds <1,000 nt for downstream analyses and will refer to the resulting assembly as “StrOccCau_0.2” hereafter (supplementary section 1.18, Supplementary Material online).

### Final Assembly Statistics

We calculated basic statistics on StrOccCau_0.2 using the “assemblathon_stats.pl” script, which was used for comparison of the Assemblathon 2 genome assemblies (Bradnam etal. 2013). We used both CEGMA version 2.5 (Parra etal. 2007) and BUSCO version 1.1b1 (Simão etal. 2015a, 2015b) to annotate sets of CEGs and thereby assess the assembly‘s completeness (supplementary section 1.19, Supplementary Material online). We also calculated basic statistics and ranCEGMA as described earlier for other available avian genomes, including the barn owl (*T. alba*) (Zhang, Li, Gilbert, et al. 2014a; Zhang, Li C, etal. 2014), downy woodpecker (*Picoides pubescens*) (Zhang, Li, Gilbert, etal. 2014b; Zhang, Li C, etal. 2014), zebra finch (*Taeniopygia guttata*) (GenBank assembly accession GCA_000151805.2; Warren etal. 2010), bald eagle (*Haliaeetus leucocephalus*) (Warren etal. 2014; Zhang, Li C, etal. 2014), golden eagle (*Aquila chrysaetos*) (GenBank assembly accession GCA_000766835.1; Wesley Warren etal. 2014), chimney swift (*Chaetura pelagica*) (Zhang, Li, Gilbert, et al. 2014c; Zhang, Li C, etal. 2014), and chicken (*Gallus gallus*) (GenBank assembly accession GCA_000002315.3; Warren etal. 2017).

### Contamination Assessment

To assess whether any assembled contigs were derived from contaminant nonvertebrate organisms, we performed a local alignment of all sequences in StrOccCau_0.2 to a copy of the NCBI nucleotide database “nt” (Clark etal. 2016; NCBI Resource Coordinators 2016) using NCBI’s BLAST+ version 2.3.0 tool BLASTN (Altschul etal. 1997; Camacho etal. 2009). We searched for nonvertebrate hits in the top aligned sequences using a local copy of the NCBI taxonomy database (ftp://ftp.ncbi.nlm.nih.gov/pub/taxonomy; Clark etal. 2016; NCBI Resource Coordinators 2016) and GItaxidIsVert (Henderson and Hanna 2016b). We re-examined those sequences where any of the five output alignments was an alignment to a nonvertebrate using the web version of NCBI’s BLAST+ version 2.4.0 tool BLASTN (Altschul etal. 1997; Camacho etal. 2009). We used bioawk version 1.0 (Li 2013b) to remove contaminant scaffolds from the assembly and will refer to the resulting assembly version hereafter as “StrOccCau_1.0.” We calculated basic statistics on StrOccCau_1.0 using the “assemblathon_stats.pl” script (Bradnam etal. 2013) (supplementary section 1.20, Supplementary Material online). We confirmed that no CEGs were present in the contaminant scaffolds.

### Mitochondrial Genome Identification

We searched for any contigs or scaffolds that were assemblies of the mitochondrial genome, rather than the nuclear genome by aligning a mitochondrial genome assembly of the brown wood owl (*Strix leptogrammica*) (GenBank Accession KC953095.1; Liu etal. 2014) to StrOccCau_1.0 using NCBI’s BLAST+ version 2.4.0 tool BLASTN (Altschul etal. 1997; Camacho etal. 2009). We searched for long alignments to scaffolds with lengths not greatly exceeding 16,500 nt, the approximate size of the mitochondrial genomes of other owl (Aves: Strigiformes) species (Harrison etal. 2004; Liu etal. 2014; Mahmood etal. 2014; Hengjiu etal. 2016). We extracted the scaffold corresponding to the mitochondrial genome assembly using bioawk version 1.0 (Li 2013b) and annotated it using the MITOS WebServer version 806 (Bernt etal. 2013) (supplementary section 1.21, Supplementary Material online). We will refer to the mitochondrial and nuclear genome assemblies hereafter as StrOccCau_1.0_mito and StrOccCau_1.0_nuc, respectively.

### Sex Identification

In order to determine the sex of the *S. o. caurina* individual that supplied the genetic sample for this genome assembly, we aligned nucleotide sequences of *S. varia* chromo-helicase-DNA binding protein-W (*CHD1W*) (GenBank Accession KF425687.1) and chromo-helicase-DNA binding protein-Z (*CHD1Z*) (GenBank Accession KF412792.1) to StrOccCau_1.0 using NCBI’s BLAST+ version 2.4.0 tool BLASTN (Altschul etal. 1997; Camacho etal. 2009). We extracted the scaffolds that aligned to the *CHD1W* and *CHD1Z* sequences using bioawk version 1.0 (Li 2013b) and then used Geneious version 9.1.4 (Kearse etal. 2012; Biomatters 2016a) to predict the length of a PCR product resulting from amplification of this region with primers 2550 F and 2718 R (Fridolfsson and Ellegren 1999) (supplementary section 1.22, Supplementary Material online).

### Repeat Annotation

We ran our genome through two separate series of repeat masking steps. The purpose of the first series was to produce a masked genome without masking of low complexity regions or simple repeats, which we could then use for downstream annotation steps. The purpose of the second series was to obtain an accurate assessment of the total repeat content of the genome, including low complexity regions and simple repeats. We first performed a homology-based repeat annotation of the genome assembly using RepeatMasker version 4.0.5 (Smit etal. 2013) and the repeat databases of the DFAM library version 1.3 (Wheeler etal. 2013) and the Repbase-derived RepeatMasker libraries version 20140131 (Jurka 1998, 2000; Jurka etal. 2005; Bao etal. 2015) without masking low complexity regions or simple repeats. We next performed a *de novo* modeling of the repeat elements in the genome using RepeatModeler version 1.0.8 (Smit and Hubley 2015) in order to create a database of repetitive regions in our genome assembly. We then further masked the genome by running RepeatMasker using the homology-based repeat-masked genome as input and the repeat database created by our RepeatModeler run and again not masking low complexity regions or simple repeats. The output was a twice-masked genome, hereafter “StrOccCau_1.0_masked.” Finally, we repeated the above steps to perform a separate homology-based and *de novo* masking of the genome with RepeatMasker runs that included masking of low complexity regions and simple repeats in order to obtain an accurate estimate of the total repeat content of the genome (supplementary section 1.23, Supplementary Material online).

### Gene Annotation

In order to annotate genes in the repeat-masked assembly, StrOccCau_1.0_masked, we followed the MAKER version 2.31.8 (Cantarel etal. 2008) pipeline as described in Campbell etal. (2014). As input for protein homology evidence, we provided MAKER the redundant protein set previously used to annotate 48 avian genomes (Zhang, Li C, etal. 2014). We used the genes found in our CEGMA run to train the gene prediction tool, Semi-HMM-based Nucleic Acid Parser or SNAP version 2006-07-28 (Korf 2004). As we independently performed repeat masking, we ran MAKER without further repeat masking. We combined all of the output gene annotations using the MAKER accessory scripts “fasta_merge” and “gff3_merge” (supplementary section 1.24, Supplementary Material online).

We assigned putative gene functions to the MAKER annotations by comparing the output MAKER protein fasta file to the Swiss-Prot UniProt release 2016_04 (Consortium 2015) database using NCBI’s BLAST 2.2.31+ tool “blastp” (Altschul etal. 1997; Camacho etal. 2009). In order to identify proteins with known functional domains, we ran InterProScan version 5.18-57.0 (Jones etal. 2014) on the protein sequences generated by MAKER. We then filtered transcripts with an Annotation Edit Distance (AED) < 1 and/or a match to a domain in Pfam, a database of protein families (Finn etal. 2016), using the script “quality_filter.pl” supplied in MAKER version 3.00.0 (Cantarel etal. 2008). We compared the unfiltered and filtered GFF3 files by analyzing the AED values for all annotations using the script “AED_cdf_generator.pl” supplied in MAKER version 3.00.0 (Cantarel etal. 2008) and graphed the distribution of values using Matplotlib pyplot (Hunter 2007) (supplementary fig. S1, Supplementary Material online). Finally, we used GenomeTools version 1.5.1 (Gremme etal. 2013) to calculate annotation summary statistics, including distributions of gene lengths, exon lengths, number of exons per gene, coding DNA sequence (CDS) lengths (measured in amino acids), and intron lengths (supplementary section 1.24, Supplementary Material online) and graphed these using Matplotlib pyplot (Hunter 2007) (supplementary figs. S2–S6, Supplementary Material online).

### Alignment

We aligned the filtered versions of all sequences from all libraries to StrOccCau_1.0_masked using the Burrows-Wheeler aligner, BWA-MEM version 0.7.12-r1044 (Li 2013a), and then merged, sorted, and marked duplicate reads using Picard version 1.104 (http://broadinstitute.github.io/picard; last accessed October 1, 2016). We then assessed the genome coverage, duplication level, and other statistics of each aligned sequence library using Picard version 1.141 (http://broadinstitute.github.io/picard; last accessed October 1, 2016) (supplementary section 1.25, Supplementary Material online). In order to obtain an estimate of the insert size of the mate pair libraries independent of the N-gaps in the scaffold sequences, we divided scaffolds into contigs at 25 or more N’s, aligned the mate pair libraries to this set of contigs using BWA-MEM version 0.7.12-r1044 (Li 2013a), and then calculated insert sizes from these alignments (supplementary section 1.25, Supplementary Material online).

### Microsatellite Analysis

We searched the repeat-masked and unmasked versions of our assembly for all microsatellite primers that have been designed from sequencing of the Mexican spotted owl (*S. o. lucida*) (Thode etal. 2002) as well as additional primers that were designed from sequences obtained from other strigid (Aves: Strigidae) species (Isaksson and Tegelström 2002; Hsu etal. 2003, 2006; Koopman etal. 2004; Proudfoot etal. 2005), but which have been used in population-level studies of *S. occidentalis* (Funk etal. 2008, 2010) and/or have been found to be useful in genetically determining F1 and F2 *S. occidentalis* × *S. varia* hybrids (Funk etal. 2007). We searched the assembly for 16 pairs of microsatellite primer sequences using NCBI’s BLAST+ version 2.4.0 tool BLASTN (Altschul etal. 1997; Camacho etal. 2009) (supplementary section 1.26, Supplementary Material online).

### Barred Owl Divergence

In order to assess the genome-wide divergence of *S. occidentalis* and *S. varia*, we extracted genomic DNA from preserved tissue of a *S. varia* collected in Hamilton County, Ohio ([CNHM<USA-OH>:ORNITH:B41533]; hereafter referred to as “CMCB41533”; table 1) using a DNeasy Blood & Tissue Kit (Qiagen). We prepared a whole-genome library with an average insert length of 466 nt using a Nextera DNA Sample Preparation Kit (Illumina) and obtained 150 nt paired-end sequence data. We performed adapter and quality trimming of the sequence data using Trimmomatic version 0.32 (Bolger etal. 2014). We aligned the trimmed sequences to StrOccCau_1.0_masked using BWA-MEM version 0.7.12-r1044 (Li 2013a) and then merged the alignments, sorted the alignments, and marked duplicate sequences using Picard version 1.104 (http://broadinstitute.github.io/picard; last accessed October 1, 2016). We then calculated alignment statistics using Picard version 1.141 (http://broadinstitute.github.io/picard; last accessed October 1, 2016). We used Genome Analysis Toolkit (GATK) version 3.4-46 UnifiedGenotyper (McKenna etal. 2010; DePristo etal. 2011; Van der Auwera etal. 2013) to call variants using the *S. occidentalis* (Sequoia) and *S. varia* (CMCB41533) BWA-MEM-aligned, sorted, duplicate-marked bam files as simultaneous inputs (supplementary section 1.27, Supplementary Material online).

We then filtered the variants to exclude indels, sites of low genotyping quality, sites where the reference individual had a homozygous alternative allele genotype, and sites with coverage greater than the mean coverage plus five times the standard deviation, as suggested by the GATK documentation (https://software.broadinstitute.org/gatk/guide/article? id=3225; last accessed October 1, 2016). We used GNU cut version 8.21 (Ihnat etal. 2013) and GNU Awk (GAWK) version 4.0.1 (Free Software Foundation 2012) to calculate *H*_w_, the mean number of nucleotide differences within *S. o. caurina* and *S. varia*, as well as *H*_b_, the number of nucleotide differences between the two species, and then used these to estimate the fixation index (*F*_ST_) (Hudson etal. 1992), a measure of population differentiation (supplementary section 1.27, Supplementary Material online). We then used an implementation of the pairwise sequentially Markovian coalescent model, PSMC version 0.6.5-r67 (Li and Durbin 2011; Li 2015), with 100 rounds of bootstrapping to estimate the effective population size (*N*_e_) through time for both *S. o. caurina* and *S. varia* (supplementary section 1.28, Supplementary Material online).

### Light-Associated Gene Analyses

We searched our *S. o. caurina* StrOccCau_1.0 assembly and the *T. alba* genome assembly (GenBank Accession GCA_000687205.1) for the presence of functional orthologs in nineteen genes that encode proteins with light-associated functions. These genes encode five visual pigment proteins (*LWS* [long wavelength-sensitive opsin], *SWS1* [short wavelength-sensitive 1 opsin], *SWS2* [short wavelength-sensitive 2 opsin], *Rh1* [rod opsin], *Rh2* [rod-like cone opsin]) (Davies etal. 2012); ten nonvisual photopigment proteins (*Opn3* [panopsin/encephalopsin], *Opn4m* [mammal-like melanopsin], *Opn4x* [*Xenopus*-like melanopsin], *Opn5* [neuropsin], *Opn5L1* [neuropsin-like 1], *Opn5L2* [neuropsin-like 2], *OpnP* [pinopsin], *RRH* [peropsin], *RGR* [retinal G protein-coupled receptor], *OpnVA* [vertebrate ancient opsin]) (Okano etal. 1994; Shen etal. 1994; Soni and Foster 1997; Sun etal. 1997; Blackshaw and Snyder 1999; Halford etal. 2001; Tarttelin etal. 2003; Bellingham etal. 2006; Tomonari etal. 2008); three enzymes involved in protection from UV radiation (*EEVS-*like, *MT-Ox*, *pOPC1* [photolyase]) (Kato etal. 1994; Osborn etal. 2015); and an enzyme involved in synthesizing red ketocarotenoid pigments (*CYP2J19* [carotenoid ketolase]) (Lopes etal. 2016; Mundy etal. 2016). We queried the genome assemblies of *S. o. caurina* and *T. alba* utilizing *in silico* probes that encompassed the exons, introns and 5′ and 3′ flanking sequences of the above genes (see supplementary table S3, Supplementary Material online for details on the probe sequences). We imported the *S. o. caurina* genome assembly into Geneious version 9.1.6 (Kearse etal. 2012; Biomatters 2016b) and used the included version of the NCBI BLAST+ BLASTn tool (Zhang etal. 2000) to search for the probes in our assembly. We used the web version of NCBI BLAST+ version 2.5.0 (Zhang etal. 2000) to align the probes against the *T. alba* genome assembly sequences in the NCBI Whole-Genome-Shotgun (WGS) contigs database. After recovering matches with our BLAST searches, we used the Geneious version 9.1.6 implementation of the MUSCLE aligner (Edgar 2004) to align the BLAST results to the probe sequences. We then used Geneious version 9.1.6 to manually adjust the alignments and examine the owl sequences for inactivating mutations, such as premature stop codons, frame shift indels (insertions/deletions), and splice site mutations. When BLAST searches were unsuccessful, we performed BLAST searches against the discarded < 1,000 nt contig set. In cases of further negative results, we used synteny data from Ensembl (version 86; Yates etal. 2016) to search for evidence of whole gene deletion (supplementary section 1.29, Supplementary Material online and supplementary table S3, Supplementary Material online). Specifically, we identified genes flanking the gene of interest in other vertebrate taxa with available contiguous genomic sequence through the relevant region, and used BLAST as noted earlier to align the reference sequences for these flanking genes to the genome assemblies of *S. o. caurina* and *T. alba*. If both flanking genes occurred on the same contig/scaffold and the intergenic sequence was not composed of missing data (N’s), this provided evidence that the gene of interest had been deleted from the genome. In order to provide further evidence of gene deletion, we used the web version of NCBI BLAST+ version 2.5.0 blastn tool (Zhang etal. 2000) to align the assembly sequence intervening the flanking genes to available sequence data in the NCBI nucleotide database “nt” (Clark etal. 2016; NCBI Resource Coordinators 2016) to search for remnant sequences of untranslated gene regions.

In instances where we discovered evidence of potentially inactivating mutations in light-associated genes of one or both owl species, we performed dN/dS ratio (ω) analyses to test whether the owl orthologs displayed evidence of relaxation of the strength of natural selection. We obtained additional ortholog sequences for the following nonowl avian species using the web version of the NCBI BLAST+ version 2.5.0 blastn tool (Zhang etal. 2000) with the discontiguous megablast option to search the NCBI nucleotide database “nt” (Clark etal. 2016; NCBI Resource Coordinators 2016): *A. chrysaetos*, turkey vulture (*Cathartes aura*), speckled mousebird (*Colius striatus*), cuckoo roller (*Leptosomus discolor*), bar-tailed trogon (*Apaloderma vittatum*), rhinoceros hornbill (*Buceros rhinoceros*), downy woodpecker (*P. pubescens*), and the northern carmine bee-eater (*Merops nubicus*) (see supplementary table S9, Supplementary Material online for sequence information). After aligning the owl gene sequences with the outgroup taxa using MUSCLE (Edgar 2004) in Geneious version 9.1.6, we adjusted the alignments manually and removed all stop codons as well as any codon positions with questionable homology. We then modeled the evolution of the genes of interest using the codeml program from the PAML version 4.8 package (Yang 2007) assuming the Prum etal. (2015) phylogeny and two separate codon frequency models (F1X4 and F3X4). We created nested models and tested for statistically significant differences in model fits using likelihood ratio tests (parameters included model = 0 [one ratio] or 2 [nested models], fix_omega = 0, NSsites = 0, see supplementary tables S10 and S11, Supplementary Material online for additional information). Most models implemented branch tests, which assumed that ω differed across branches on the phylogeny, but was equal across a gene. We estimated the foreground ω on the *Tyto* branch for *OpnP*, the *Strix* and *Tyto* branches for *CYP2J19* and *Rh2*, and the crown (*Strix*+ *Tyto*) and stem Strigiformes branches for *Opn4m*. The background ω for each gene consisted of the remaining branches. In a few instances, we implemented branch-sites tests, which assumed differences in ω across the phylogeny while allowing for different ω values across different portions of a gene (parameters included model = 2, fix_omega = 1 [null] or 0 [alternative], omega = 1, NSsites = 2).

We additionally used the NCBI BLAST+ version 2.5.0 blastn tool (Zhang etal. 2000) with the discontiguous megablast option to align a reference *Opn4m* sequence to fifteen avian retinal transcriptomes, which included six owl species (Wu etal. 2016) in NCBI’s Sequence Read Archive (SRA) (Leinonen etal. 2011; NCBI Resource Coordinators 2016) (see supplementary section 1.29, Supplementary Material online for additional transcriptome information). We imported the short reads that aligned into Geneious version 9.1.6 (Kearse etal. 2012; Biomatters 2016b) and mapped them to the reference sequence using the Geneious “map to reference” function and trying both the “medium sensitivity/fast” and “low sensitivity/fastest” settings.

## Results

### Contamination Assessment

Our search for nonvertebrate sequences in our assembly suggested that our assembly was only very minimally contaminated with nonvertebrate sequences. For only nine out of the 8,113 final assembly scaffolds, one of the five top alignments to the NCBI nucleotide database (Clark etal. 2016; NCBI Resource Coordinators 2016) was an alignment to a non-vertebrate sequence. Four of these scaffolds were short, ranging from 1,182 to 2,304 nt, and aligned to *Escherichia coli* sequence data. We removed these four scaffolds from the assembly. We kept the other five scaffolds in the assembly. The highest BLAST bit-score for scaffold-1085 was for an alignment to the telomere region of a human genome with 81% identity across 53% of the scaffold. The highest BLAST bit-scores for scaffold-1155 were for alignments to endogenous retrovirus regions of several vertebrate genomes. Three scaffolds (2014, 2160, and 3069) were longer scaffolds that aligned to vertebrate genome sequences with only small sequence portions that aligned to nonvertebrate sequence data; we did not feel this justified removing them from the assembly.

### Mitochondrial Genome Identification

We identified scaffold-3674 as an assembly of the mitochondrial genome as it had a 14,649 nt alignment with 89.1% similarity to the *S. leptogrammica* mitochondrial genome. This length was the majority of the 21,628 nt scaffold-3674. After subtracting a block of 3,984 N’s present in the scaffold, the length of scaffold-3674 is similar to that of other avian mitochondrial genomes (Mindell etal. 1997, 1998, 1999; Guan etal. 2016; Zhang etal. 2016). We were able to annotate all of the standard avian mitochondrial genes, except *ND6* and *tRNA^Pro^*, which suggests that this assembly of the mitochondrial genome could be improved.

### Genome Size

Our *k*-mer-based estimation with Preqc yielded an estimated genome length of 1.29 giga nucleotides (Gnt). This type of estimation generally underestimates the true genome size as it collapses *k*-mers from highly repetitive regions. The total length of all sequences in our gap-closed assembly was 1.88 Gnt, but this length included all singleton sequences (many of which were unassembled reads) and N-filled gaps. After removing all contigs and scaffolds <1,000 nt, the combined total length of all scaffolds was 1.26 Gnt.

### Assembly Statistics

Gap-closing improved the assembly continuity and completeness metrics (tables 3 and 4). Removing shorter length contigs/scaffolds improved the post gap closing assembly metrics at both the contig and the scaffold level. The unfiltered assembly had a scaffold N50 length of 1.836 Mnt and a contig N50 length of 81,400 nt. Removing contigs/scaffolds less than 300 nt increased the scaffold and contig N50 lengths over 2× to 3.916 Mnt and 168,721 nt, respectively, and generated the greatest relative increase in the other continuity metrics of any of the filtering options that we tried (supplementary table S4, Supplementary Material online). The highest scaffold and contig N50 lengths (3.983 Mnt and 171,882 nt, respectively) and the best other continuity metrics resulted from removing all contigs and scaffolds <1,000 nt, but this came at the slight expense of the completeness of the genome (supplementary table S4, Supplementary Material online; tables 3 and 4). Our gap-closed genome included complete sequences of 228 and at least partial sequences of 236 of the 248 CEGMA orthologs. We only lost one of these when we removed contigs and scaffolds <1,000 nt and retained 228 complete and 235 partial CEGMA orthologs in the filtered assembly (table 4). Except for the percentage of duplicated orthologs, which was notably higher as measured by the CEGMA analysis versus the BUSCO analysis, the results of the CEGMA and BUSCO analyses closely agreed. Both found at least partial sequences of over 90% of the conserved orthologs (235/248 = 94.76% CEGMA and 2,815/3,023 = 93.12% BUSCO orthologs) under scrutiny in the final assembly (table 4). Our final assembly contained 8,113 scaffolds and/or contigs with a scaffold N50 of 3.98 Mnt. The longest scaffold was 15.75 Mnt. The GC content was 41.31%. The N content was 1.10%.

**Table 3:** Final Assembly Metrics

Assembly Version	No Gap-Closing, no Scaffolds, or Contigs Removed	Gap-Closed, No Scaffolds or Contigs Removed	Gap-Closed, Scaffolds and Contigs <1,000 nt Removed
Number of scaffolds	3,754,960	3,754,960	8,108
Total size of scaffolds	1,884,397,264 nt	1,882,081,621 nt	1,255,541,132 nt
Longest scaffold	15,783,852 nt	15,750,186 nt	15,750,186 nt
Shortest scaffold	128 nt	128 nt	1,000 nt
Number of scaffolds > 1 K nt	8,112 (0.2%)	8,095 (0.2%)	8,095 (99.8%)
Number of scaffolds > 10 K nt	1,754 (0.0%)	1,746 (0.0%)	1,746 (21.5%)
Number of scaffolds > 100 K nt	661 (0.0%)	661 (0.0%)	661 (8.2%)
Number of scaffolds > 1 M nt	303 (0.0%)	303 (0.0%)	303 (3.7%)
Number of scaffolds > 10 M nt	9 (0.0%)	9 (0.0%)	9 (0.1%)
Mean scaffold size	502 nt	501 nt	154,852 nt
Median scaffold size	150 nt	150 nt	1,904 nt
N50 scaffold length (L50 scaffold count)	1,843,286 nt (209)	1,836,279 nt (209)	3,983,020 nt (92)
N60 scaffold length (L60 scaffold count)	622,124 nt (370)	619,581 nt (371)	3,012,707 nt (129)
N70 scaffold length (L70 scaffold count)	255 nt (216,251)	255 nt (218,976)	2,162,240 nt (178)
N80 scaffold length (L80 scaffold count)	174 nt (1,110,583)	174 nt (1,113,245)	1,545,070 nt (246)
N90 scaffold length (L90 scaffold count)	143 nt (2,336,958)	143 nt (2,338,577)	618,731 nt (372)
scaffold %GC	42.81%	43.82%	41.31%
scaffold %N	2.89%	0.74%	1.10%
Percentage of assembly in scaffolded contigs	66.4%	65.7%	98.5%
Percentage of assembly in unscaffolded contigs	33.6%	34.3%	1.5%
Average number of contigs per scaffold	1.0	1.0	3.4
Average length of break (>25 Ns) between contigs in scaffold	311	703	716
Number of contigs	3,929,029	3,774,552	27,252
Number of contigs in scaffolds	179,939	22,372	21,478
Number of contigs not in scaffolds	3,749,090	3,752,180	5,774
Total size of contigs	1,830,109,624 nt	1,868,296,631 nt	1,241,823,123 nt
Longest contig	186,255 nt	1,259,046 nt	1,259,046 nt
Shortest contig	5 nt	128 nt	130 nt
Number of contigs > 1 K nt	123,891 (3.2%)	23,915 (0.6%)	23,915 (87.8%)
Number of contigs > 10 K nt	37,347 (1.0%)	12,373 (0.3%)	12,373 (45.4%)
Number of contigs > 100 K nt	58 (0.0%)	3,909 (0.1%)	3,909 (14.3%)
Number of contigs > 1 M nt	0 (0.0%)	8 (0.0%)	8 (0.0%)
Mean contig size	466 nt	495 nt	45,568 nt
Median contig size	150 nt	150 nt	6,702 nt
N50 contig length (L50 contig count)	7,855 nt (46,856)	81,400 nt (4,678)	171,882 nt (2,057)
N60 contig length (L60 contig count)	3,275 nt (81,600)	33,521 nt (8,121)	134,419 nt (2,876)
N70 contig length (L70 contig count)	254 nt (448,715)	255 nt (254,729)	98,604 nt (3,955)
N80 contig length (L80 contig count)	170 nt (1,346,255)	173 nt (1,148,692)	66,668 nt (5,484)
N90 contig length (L90 contig count)	142 nt (2,548,877)	142 nt (2,367,845)	34,559 nt (8,023)

Note.—Assembly (contaminant and mitochondrial sequences removed) metrics before gap-closing,after gap-closing,and after both gap-closing and removal of all contigs and scaffolds <1,000 nt in length. Strings of 25 or more N’s broke scaffolds into contigs.

**Table 4:** Summary of Conserved Ortholog Searches

Assembly	Draft. No Gap-Closing, Contigs/Scaffolds < 300 nt Removed	Draft. Gap-Closed, No Removal of Small Contigs/Scaffolds	Final. Gap-Closed, Contigs/Scaffolds <1,000 nt Removed	Final. Gap-Closed, Contigs/Scaffolds <1,000 nt Removed
Method	**CEGMA**	**CEGMA**	**CEGMA**	**BUSCO**
Total conserved orthologs examined	248	248	248	3,023
Complete orthologs (% of total)	221 (89.11%)	228 (91.94%)	228 (91.94%)	2,605 (86.17%)
At least partial orthologs (% of total)	235 (94.76%)	236 (95.16%)	235 (94.76%)	2,815 (93.12%)
Duplicated orthologs (% of total)	92 (37.10%)	83 (33.47%)	99 (39.92%)	46 (1.52%)
Missing orthologs	13 (5.24%)	12 (4.84%)	13 (5.24)%	208 (6.88%)

Note.—Comparison of the number of conserved orthologous genes found in the final assembly (gap-closed,contigs/scaffolds <1,000 nt removed) using the CEGMA and BUSCO tools. In order to illustrate the effect of gap-closing and removal of small fragments on assembly completeness metrics,also included are the results of CEGMA gene searches conducted on two draft versions of the final assembly where we either did not perform gap-closing and removed contigs/scaffolds < 300 nt or performed gap-closing and did not remove any small contigs/scaffolds.

The contig-level continuity statistics improved substantially when we allowed for longer blocks of intervening N’s before demarcating separate contigs (supplementary table S4, Supplementary Material online). Relative to delineating contigs at every N (contig N50 of 51,301 nt), allowing up to 5 N’s before demarcating a separate contig yielded an over 3× increase in the contig N50 of 155,200 nt. This was the greatest relative increase that we saw in the contig N50 length out of all the intervening N lengths that we tried, (supplementary table S4, Supplementary Material online). Allowing up to 25 N’s before demarcating a separate contig resulted in the highest contig N50 (171.88 kilo nucleotides (knt); supplementary table S4, Supplementary Material online). In both continuity and completeness, our assembly compares favorably with those of the other avian genomes for which we calculated equivalent metrics (table 5).

**Table 5:** Comparative Statistics of Avian Genomes

Species	Common name	Scaffold N50 (nt)	No. Scaffolds/Contigs	Contig N50 (nt)	Length (Gnt)	Ns (%)	Complete CEGs (% of 248)	Partial CEGs (% of 248)
*S. o. caurina*	Northern Spotted Owl	3,983,020	8,108	171,882	1.26	1.10	228 (91.94%)	235 (94.76%)
*T. alba*	Barn Owl	51,873	166,092	19,113	1.14	1.02	144 (58.06%)	198 (79.84%)
*P. pubescens*	Downy Woodpecker	2,086,781	85,828	29,578	1.17	3.72	196 (79.03%)	216 (87.10%)
*T. guttata*	Zebra Finch	62,374,962	37,095	38,644	1.23	0.75	192 (77.42%)	214 (86.29%)
*H. leucocephalus*	Bald Eagle	669,725	346,419	10,218	1.26	3.97	217 (87.50%)	240 (96.77%)
*A. chrysaetos*	Golden Eagle	9,230,743	1,141	215,151	1.19	1.07	226 (91.13%)	238 (95.97%)
*C. pelagica*	Chimney swift	3,839,435	60,234	33,918	1.13	4.02	191 (77.02%)	222 (89.52%)
*G. gallus*	Chicken	82,310,166	23,474	2,905,620	1.23	0.96	226 (91.13%)	237 (95.56%)

Note.—Comparative statistics of our *S. o. caurina* assembly with those of a selection of other avian genome assemblies.

### Sex Identification

We determined from our assembly that the sequence came from the genome of a female *S. o. caurina*. The lengths of the *CHD1* markers on the sex chromosomes were 634 and 1,058 nt on scaffolds 806 and 4429, respectively. These lengths are in the size range of those amplified from *S. nebulosa* samples by previous researchers (600–650 and 1,200 nt for *CHD1Z* and *CHD1W*, respectively) (Fridolfsson and Ellegren 1999) and suggest that scaffolds 806 and 4429 are sequences from the Z and W chromosomes, respectively.

### Repeat Annotation

The repeat annotation and masking of the genome examined 3,754,965 individual sequences totaling 1,882,109,172 nt. The homology-based repeat annotation resulted in GC content estimation of 44.15% and masked 21.02% of the assembly as repetitive. Repeat masking using a *de novo* model of the repeat elements estimated that an additional 0.55% of the assembly was repetitive. Due to the fact that some of the annotated repetitive elements overlapped, the following repeat category percentage values do not exactly sum to the 21.57% total genome repeat content. Interspersed repeat elements including retroelements, DNA elements (DNA transposons with no RNA intermediate), and unclassified elements comprised 9.31% of the assembly; of these, retroelements were the most common, constituting 8.96% of the assembly (table 6). Non-interspersed repeat elements including small RNA elements, satellites, simple repeats, and low complexity repeats comprised 12.33% of the assembly; of these, satellites were the most common, constituting 9.88% of the assembly.

**Table 6:** Repetitive Element Summary

Type Level 1	Type Level 2	Type Level 3	Type Level 4	Number of Elements	Element Total Length (nt)	Assembly Portion (%)
**Total interspersed repeats**					175,287,790	9.31
	**Total retroelements**			727,006	168,672,903	8.96
	Retroelement	SINE		40,360	4,770,020	0.25
	Retroelement	SINE	ALU	53	6,194	0.00
	Retroelement	SINE	MIR	15,510	1,558,420	0.08
	Retroelement	Penelope		169	35,110	0.00
	Retroelement	Total LINEs		486,310	115,604,290	6.14
	Retroelement	LINE	LINE1	622	58,117	0.00
	Retroelement	LINE	LINE2	3,116	317,864	0.02
	Retroelement	LINE	L3/CR1	28,122	5,153,289	0.27
	Retroelement	LINE	CRE/SLACS	0	0	0.00
	Retroelement	LINE	L2/CR1/Rex	452,030	109,807,316	5.83
	Retroelement	LINE	R1/LOA/Jockey	0	0	0.00
	Retroelement	LINE	R2/R4/NeSL	131	44,590	0.00
	Retroelement	LINE	RTE/Bov-B	15	3,492	0.00
	Retroelement	LINE	L1/CIN4	98	23,441	0.00
	Retroelement	Total LTR elements		200,336	48,298,593	2.57
	Retroelement	LTR	BEL/Pao	0	0	0.00
	Retroelement	LTR	ERV_classI	983	122,219	0.01
	Retroelement	LTR	ERV_classII	400	54,854	0.00
	Retroelement	LTR	ERVL	436	91,660	0.00
	Retroelement	LTR	ERVL-MaLRs	51	4,838	0.00
	Retroelement	LTR	Gypsy/DIRS1	111	14,921	0.00
	Retroelement	LTR	Retroviral	197,967	47,947,799	2.55
	Retroelement	LTR	Ty1/Copia	0	0	0.00
	**Total DNA elements**			37,526	5,628,486	0.30
	DNA element		En-Spm	0	0	0.00
	DNA element		hAT-Charlie	418	28,220	0.00
	DNA element		hobo-Activator	4,235	719,417	0.04
	DNA element		MuDR-IS905	0	0	0.00
	DNA element		PiggyBac	0	0	0.00
	DNA element		Tc1-IS630-Pogo	806	141,663	0.01
	DNA element		TcMar-Tigger	528	39,074	0.00
	DNA element		Tourist/Harbinger	9,255	958,360	0.05
	DNA element		Other (Mirage, P-element,Transib)	0	0	0.00
	**Rolling-circles**			0	0	0.00
	**Unclassified interspersed repeats**			6,225	986,401	0.05
**Total noninterspersed repeats**				1,907,394	232,038,709	12.33
	Small RNA			12,051	1,645,166	0.09
	Satellites			1,261,021	185,995,538	9.88
	Simple repeats			564,508	40,568,395	2.16
	Low complexity repeats			69,814	3,829,610	0.20

Note.—Summary of the repeat elements found during two rounds of repeat masking (homology-based followed by denovo-model-based masking). Depending on the type of repeat element, we provide information at different category summary levels. We use the “Type level” column headings to organize these categories.

### Gene Annotation

The MAKER pipeline succeeded in annotating all contigs and scaffolds except one, scaffold-1363, which is 555,526 nt long and failed the annotation pipeline for an unknown reason. The MAKER pipeline‘s implementation of AUGUSTUS version 3.2.1 (Keller et al. 2011; Stanke 2015) predicted 19,692 proteins and transcripts *ab initio*. After quality filtering, we retained 16,718 annotated proteins and transcripts, 5,062 of which were nonoverlapping *ab initio* predictions of proteins and transcripts.

Annotated gene sequence lengths ranged from 51 to 282,544 nt with a median length of 9,187.50 nt (supplementary fig. S2, Supplementary Material online). Coding sequence lengths varied from 51 to 66,303 nt with a median length of 1,137 nt (supplementary fig. S3, Supplementary Material online). Exon lengths extended to a maximum of 14,832 nt with a median length of 130 nt (supplementary fig. S4, Supplementary Material online). Intron lengths ranged from 45 to 57,529 nt with a median length of 910 nt (supplementary fig. S5, Supplementary Material online). The number of exons per gene ranged from 1 exon to 142 exons with a median number of six exons per gene (supplementary fig.S6, Supplementary Material online).

### Alignment

The assembly contained 1,142, 612,682 nonN bases used in the calculation of the library alignment statistics. After all filters, the total mean coverage for the paired and unpaired data from all of the sequenced libraries aligned to the repeat-masked genome was 60.43×. The MP11 kb mate-pair library had the highest proportion of duplicate bases (60.1%) and the PCR-free library noPCR550 bp had the lowest (0.3%) (table 7).

**Table 7:** Library Alignment Statistics

Library	Mean Paired and Unpaired Read Genome Coverage Postfiltering (X)	SD of Paired and Unpaired Read Genome Coverage Postfiltering (X)	Fraction of Aligned Bases From Unpaired Reads	Total Fraction of Filtered Aligned Bases	Fraction Aligned Bases Filtered Due to Mapping Quality < 20	Fraction Aligned Bases Filtered as Duplicates	Fraction Aligned Bases Filtered as Low Quality With Q < 20	Fraction Aligned Bases Filtered as Second Observation From Overlapping Reads	Fraction Aligned Bases Filtered From Regions Already with > 1,000× coverage
Nextera350bp lane 1	4.369	5.484	0.048	0.533	0.060	0.444	0.004	0.023	1.52E-03
Nextera350bp lane 2	11.162	8.960	0.039	0.559	0.056	0.480	0.005	0.017	1.43E-03
Hydroshear	1.093	2.784	0.004	0.549	0.033	0.429	0.005	0.081	2.03E-03
Nextera550bp lane 1	2.741	3.708	0.393	0.096	0.034	0.038	0.011	0.011	1.05E-03
Nextera550bp lane 2	5.790	5.435	0.327	0.126	0.032	0.066	0.019	0.008	1.26E-03
Nextera700bp	23.357	14.710	0.041	0.216	0.046	0.126	0.009	0.032	3.64E-03
noPCR550bp	3.244	2.661	0.241	0.059	0.013	0.003	0.014	0.029	4.32E-04
PCR900bp	1.978	1.894	0.073	0.052	0.012	0.024	0.014	0.001	3.34E-04
MP4kb	2.528	2.745	0.300	0.361	0.048	0.306	0.002	0.004	5.36E-04
MP7kb	2.528	2.734	0.256	0.449	0.045	0.397	0.002	0.004	4.53E-04
MP11kb	1.641	2.205	0.168	0.652	0.046	0.601	0.001	0.004	2.56E-04
CMCB41533	15.552	12.253	0.030	0.341	0.299	0.037	2.37E-04	2.59E-03	2.50E-03

Note.—Alignment statistics for all Sequoia (*Strix occidentalis caurina*) libraries and the CMCB41533 (*Strix varia*) library calculated using Picard’s CollectWgsMetrics.

Insert sizes of mate pair libraries determined by mapping quality-filtered reads back to the genome assembly gave lower inserts than were expected based on bioanalyzer traces. Whereas the bioanalyzer traces gave evidence that the MP4, MP7, and MP11 kb libraries had insert lengths of ∼4.2, 7.1, and 10.7 knt, respectively, the results from mapping to the whole genome assembly suggested that the insert lengths were instead 3.3, 5.9, and 9.6 knt, respectively. We hypothesized that this difference may have been due to the number of N’s added during scaffolding, we also mapped the sequences from these libraries to the assembly with all scaffolds decomposed into their constituent contigs. This yielded average insert sizes of 3.3, 6.0, and 10.0 knt, which suggest some potential for improving N gap lengths, but that the N stretches in the scaffolds are good approximations of the lengths of missing, intervening sequences.

### Microsatellite Analysis

We found 15 out of the 16 pairs of microsatellite primers for which we searched in the genome assembly (table 8). We found loci 4E10, 4E10.2, and Oe149 on scaffold-11. The distance from the forward 4E10.2 primer to the forward 4E10 primer is 12,172 nt in our assembly, which confirms the characterization of the loci 4E10 and 4E10.2 as linked within 40 kb by the original authors who described these loci using sequences obtained from the same cosmid (Thode etal. 2002). The reverse 4E10 primer is 717,153 nt distant from the forward Oe149 primer. The remaining primer pairs aligned to separate assembly scaffolds (table 8).

**Table 8:** Genomic Locations of Selected Microsatellite Loci

Locus	Primer	References	Usage Comments	Length Primer	Length Alignment	Mismatches	Genome Scaffold	Genome Start	Genome End	Microsatellite Length (nt)
13D8	F	(Thode etal. 2002)	population genetics (Funk etal. 2008, 2010)	22	22	0	scaffold88	4,241,040	4,241,019	187
13D8	R			21	21	0	scaffold88	4,240,854	4,240,874	
15A6	F	(Thode etal. 2002)	population genetics (Funk etal. 2008, 2010)	21	21	0	scaffold233	2,208,703	2,208,723	148
15A6	R			19	16	0	scaffold233	2,208,847	2,208,832	
1C6	F	(Thode etal. 2002)	None	20	20	0	scaffold178	2,550,734	2,550,753	110
1C6	R			20	20	0	scaffold178	2,550,843	2,550,824	
4E10	F	(Thode etal. 2002)	None	22	22	0	scaffold11	768,391	768,371	230
4E10	R			22	22	0	scaffold11	768,162	768,183	
4E10.2	F	(Thode etal. 2002)	population genetics (Funk etal. 2008, 2010)	18	18	0	scaffold11	780,562	780,579	226
4E10.2	R			18	18	0	scaffold11	780,787	780,770	
6H8	F	(Thode etal. 2002)	population genetics (Funk etal. 2008, 2010)	21	21	0	scaffold103	3,773,885	3,773,865	93
6H8	R			16	16	0	scaffold103	3,773,793	3,773,808	
8G11	F	(Thode etal. 2002)	None	18	—	—	—	—	—	—
8G11	R			17	—	—	—	—	—	
Bb126	F	(Isaksson & Tegelström 2002)	hybrid diagnostic (Funk etal. 2007)	20	20	0	scaffold219	2,548,147	2,548,166	185
Bb126	R			24	24	0	scaffold219	2,548,331	2,548,308	
BOOW18	F	(Koopman etal. 2004)	hybrid diagnostic (Funk etal. 2007)	19	19	1	scaffold244	648,444	648,426	205
BOOW18	R			20	20	1	scaffold244	648,240	648,259	
FEPO5	F	(Proudfoot etal. 2005)	population genetics (Funk etal. 2008, 2010)	22	22	0	scaffold138	720,315	720,336	270
FEPO5	R			25	25	2	scaffold138	720,584	720,560	
Oe045	F	(Hsu etal. 2003)	hybrid diagnostic (Funk etal. 2007)	23	23	2	scaffold173	3,777,655	3,777,677	127
Oe045	R			19	19	0	scaffold173	3,777,781	3,777,763	
Oe053	F	(Hsu etal. 2003)	population genetics (Funk etal. 2008, 2010)	23	23	1	scaffold136	299,240	299,262	218
Oe053	R			22	22	1	scaffold136	299,457	299,436	
Oe128	F	(Hsu etal. 2003)	hybrid diagnostic (Funk etal. 2007),population genetics (Funk etal. 2008, 2010)	27	27	0	scaffold722	802,232	802,206	319
Oe128	R			24	24	0	scaffold722	801,914	801,937	
Oe129	F	(Hsu etal. 2006)	population genetics (Funk etal. 2008, 2010)	24	21	2	scaffold529	3,066,759	3,066,739	266
Oe129	R			24	24	1	scaffold529	3,066,497	3,066,520	
Oe149	F	(Hsu etal. 2006)	population genetics (Funk etal. 2008, 2010)	21	21	1	scaffold11	51,010	50,990	258
Oe149	R			20	20	0	scaffold11	50,753	50,772	
Oe3-7	F	(Hsu etal. 2003)	population genetics (Funk etal. 2008, 2010)	20	19	1	scaffold35	572,329	572,347	129
Oe3-7	R			23	23	0	scaffold35	572,456	572,434	

Note.—Locations of commonly used microsatellite loci in our draft genome assembly. We searched for all of the primer pairs used in several *S. occidentalis* population genetics studies as well all of those designed for use in *S. o. lucida* (Thode etal. 2002). The “Primer” column designates the forward or reverse primer with “F” or “R,” respectively. The “Reference” column gives the citation of the publication that originally described each primer pair. The “Comment” column gives the citation(s) of the publication(s) in which a primer pair has been used for population-level study of *S. occidentalis* or and/or study of *S. occidentalis* x *S. varia* hybrids. “Length alignment” refers to the length of the BLASTN (Altschul etal. 1997; Camacho etal. 2009) alignment. The “Microsatellite length” refers to the inferred length of the microsatellite PCR product based on the length of the primers and their mapping positions in the genome assembly.

### Barred Owl Divergence

We estimated the nuclear genome-wide nucleotide diversity (*H*_w_) of *S. o. caurina* as 2.008 × 10^−4^ and that of *S. varia* as 2.352 × 10^−3^. We estimated the genome-wide nucleotide diversity between *S. o. caurina* and *S. varia* (*H*_b_) as 7.042 × 10^−3^ and calculated an *F*_ST_ of 0.819.

### PSMC Analysis

**Fig. 1:**
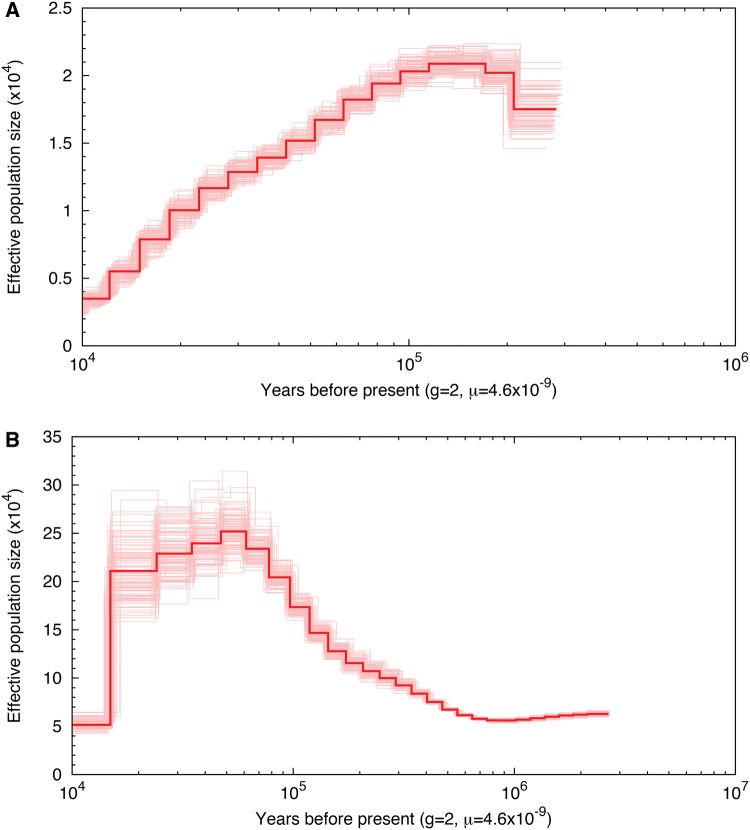
—Demographic history of *Strix occidentalis caurina* and *Strix varia* with bootstrap replicates. (Panel A) depicts the demographic history estimated for *S. o. caurina*. (Panel B) depicts the demographic history estimated for *Strix varia*.

Our pairwise sequentially Markovian coalescent (PSMC) model analyses suggested that the *N_e_* of both *S. o. caurina* and *S. varia* was substantially higher in the past and has been in decline since ∼100,000 or 80,000 years before present, respectively (fig. 1). The estimated peak *N_e_* of *S. o. caurina* was more than an order of magnitude lower than that of *S. varia* (∼20,000 and 250,000 for *S. o. caurina* and *S. varia*, respectively). The most recent estimate that the PSMC analysis provided for the *N*_e_ of *S. o. caurina* was also more than an order of magnitude lower than that of *S. varia* (∼4,000 and 50,000 for *S. o. caurina* and *S. varia*, respectively).

### Light-Associated Gene Analyses

Seven of the nineteen genes encoding proteins with light-associated functions that we examined displayed evidence of inactivation or whole gene deletion in one or both owl species (supplementary table S3, Supplementary Material online; Hanna etal. 2017). We found no BLAST alignments of *SWS1* to either the *S. o. caurina* or the *T. alba* assembly. However, the genes flanking *SWS1* in zebra finch (*T. guttata*) and human (*Homo sapiens*), *FLNC* (Filamin-C) and *CALU* (Calumenin) (Ensembl version 86; Yates etal. 2016), are both present in the *S. o. caurina* genome assembly, but they are located on different scaffolds. Without increased genomic continuity, it is difficult to discern whether chromosomal rearrangement has occurred or whether this is a case of simple gene deletion. Recent searches in crocodilian (Crocodilia) genomes similarly found *FLNC* and *CALU* on separate contigs with *SWS1* missing from the assemblies (Emerling 2017a), which suggests that this may be a problematic region to assemble. NCBI’s Eukaryotic Genome Annotation (EGA) pipeline did not find *FLNC* and *CALU* in the *T. alba* genome assembly (NCBI *T. alba* Annotation Release 100; NCBI Accession GCF_000687205.1), but the absence of these genes in the assembly may be due to low assembly quality (Zhang, Li B, Li C, etal. 2014).

*SWS2* and *LWS* are adjacent on the same chromosome in the Carolina anole (*Anolis carolinensis*) and African clawed frog (*Xenopus laevis*) genome assemblies and are flanked by *MECP2* (methyl-CpG binding protein 2) in *A. carolinensis* and *X. laevis*, *AVPR2* (arginine vasopressin receptor 2) in *X. laevis*, and *TEX28* (testis expressed 28) in *A. carolinensis* (Ensembl version 86; Yates etal. 2016). We did not obtain BLAST alignments to *SWS2* or *LWS* for the *T. alba* assembly and NCBI’s EGA pipeline did not find *MECP2*, *AVPR2*, or *TEX28* (NCBI *T. alba* Annotation Release 100; NCBI Accession GCF_000687205.1), which suggests that this portion of the genome, like the *SWS1* region, may be challenging to assemble. Although we found *SWS2* and *LWS* in our *S. o. caurina* assembly, we only obtained partial coding sequences with elevated GC content of 66.9% and 68.0%, respectively. Our *S. o. caurina* assembly contained a partial *SWS2* exon 1 sequence as well as complete exon 2 and 3 sequences with all three exons found on two separate scaffolds (scaffolds 4153 and 7110). The sequences of these exons on the two scaffolds were 100% identical except for one difference in exon 3. Given the high sequence similarity and the recovery of the same portions of the *SWS2* coding region, these duplicate sequences are likely an artifact of the assembly process and do not indicate gene duplication.

*SWS2*, *LWS*, *Rh1*, and *Rh2* in *S. o. caurina* and *Rh1* in *T. alba* showed no evidence of potentially inactivating mutations. However, *Rh2* in *T. alba* displayed a 29 nt deletion in exon 1, single premature stop codons in both exons 2 and 3, and a 2 nt deletion in exon 4. Our modeling of the sequence evolution of *Rh2* in *S. o. caurina* and *T. alba* yielded evidence that selection has become relaxed in *T. alba* (ω = 0.22–0.37; *P* < 0.00001) relative to other avian taxa (ω = 0.03–0.06), which is consistent with pseudogenization of this gene. A branch test of *S. o. caurina* also displayed evidence of relaxed selection on *Rh2* with an elevated ω (0.16–0.21; *P* < 0.05) relative to the background. Our branch-sites test evaluated whether there was indication of positive selection across a subset of sites, but it did not yield any evidence that the elevated ω was due to adaptive evolution. We did find nine missense mutations in *S. o. caurina* that were not found in any of the non-owl avian species, but none of these were at known conserved sites (Carleton etal. 2005), which suggests that they have not resulted in a loss of function.

We were unable to recover *OpnP* in our *S. o. caurina* assembly, but together on the same scaffold we found the genes that flank *OpnP* in the chicken (*G. gallus*) and the collared flycatcher (*Ficedula albicollis*) genome assemblies, *TEX14* (testis expressed sequence 14) in *G. gallus* and *DOC2B* (double C2 domain beta) in *G. gallus* and *F. albicollis* (Ensembl version 86; Yates etal. 2016). Our BLAST of the sequence intervening *TEX14* and *DOC2B* in our *S. o. caurina* assembly revealed similarity (8% query coverage, 82% identity) with the 5′ untranslated region of *G. gallus OpnP*. Together, these provide strong evidence of whole gene deletion of *OpnP* in *S. o. caurina*. *OpnP* in *T. alba* is a pseudogene with numerous inactivating mutations, including the following: a start codon mutation (ACA), 13 nt deletion, 2 nt insertion, and 1 nt deletion in exon 1, a 1 nt deletion in exon 2, a 21 nt deletion of the intron 3-exon 4 boundary, a 7 nt deletion and 2 nt deletion in exon 4, and a 1 nt deletion in exon 5. We assembled sequences from outgroup taxa and confirmed that these mutations are unique to *T. alba*. Our dN/dS ratio analyses strongly suggested relaxed selection on the *T. alba* branch (ω = 0.51–0.7; *P* < 0.00001) compared with purifying selection on the background branches (ω = 0.11–0.18).

*Opn4m* displays evidence of inactivation in both *S. o. caurina* and *T. alba*, with both species sharing a 4 nt deletion in exon 8. Additionally, *S. o. caurina* has a premature stop codon in exon 8 and *T. alba* possesses a splice donor mutation (GT to AT) in intron 11. Comparisons with outgroup taxa confirmed that these mutations were unique to owls, but also demonstrated that other bird species have putative inactivating mutations in this gene, including the golden eagle (*A. chrysaetos*) with a premature stop codon in exon 9; speckled mousebird (*C. striatus*) with a 1 nt deletion in exon 9, splice donor mutation in intron 9 (GT to TT), and premature stop codon exon 11; cuckoo roller (*L. discolor*) with a splice donor mutation in intron 10 (GT to GA); and rhinoceros hornbill (*B. rhinoceros*) with a start codon mutation (ATG to CTG). We performed dN/dS ratio analyses after removing all exons that contained putative inactivating mutations. The results indicated that the average ω for the crown owl branches is elevated (ω = 0.45; *P* < 0.01) relative to the background (ω = 0.19), which does not meet the expectation of neutral evolution predicted if the shared 4 nt deletion led to a loss of function of *Opn4m*. Branch-sites tests yielded evidence of positive selection on some portions of the gene for both owl branches, but this signal was not a significantly better fit than the null. Our BLAST of an *Opn4m* sequence to fifteen bird retinal mRNA short read databases, which included data from six owl species, yielded alignments to all fifteen transcriptomes. Further investigation of these sequences in Geneious revealed evidence of different isoforms of *Opn4m*. When we used lower sensitivity alignment settings, the assemblies of mapped sequences generally terminated after exon 8 (the exon with the 4 nt deletion), suggesting that this is an abundant transcript isoform. However, using higher sensitivity alignment settings generated assemblies of multiple transcripts with distinct sequences at some of the exon–intron boundaries.

Finally, *CYP2J19* displays evidence of inactivation in both owl species. *S. o. caurina* has a 1 nt insertion and 2 nt deletion in exon 9. As Emerling (2017c) described, the *T. alba* assembly contains a premature stop codon in each of exons 1, 5, and 6 as well as a 5 nt deletion in exon 3. Both the *S. o. caurina* (ω = 0.33–0.34; *P* < 0.05) and *T. alba* (ω = 0.68–0.72; *P* < 0.0001) branches have elevated dN/dS ratios compared with the background (0.15–0.16), which is consistent with the hypothesis that these mutations have led to a loss of function of *CYP2J19*.

## Discussion

### Genome Characterization

Direct comparison of assembly metrics between our *S. o. caurina* assembly and seven other avian genome assemblies, including the avian model organisms chicken (*G. gallus*) and zebra finch (*T. guttata*), revealed that the *S. o. caurina* assembly is in the top tier of genomes in both continuity and completeness (table 5). Only the golden eagle (*A. chrysaetos*), zebra finch, and chicken genomes had better continuity statistics as measured by scaffold and contig N50s. We compared the relative completeness of the assemblies by searching for a set of 248 CEGs using CEGMA. Of the assemblies that we compared, we found the highest number of complete conserved gene sequences in our *S. o. caurina* assembly (228 complete CEGs), surprisingly surpassing even the chicken genome (226 complete CEGs). In terms of at least partially complete sequences of conserved genes, our *S. o. caurina* assembly contained only two fewer than the chicken genome (235 vs. 237 partial CEGs). Our assembly is both more complete and more contiguous than that of *T. alba*, the only other owl assembly currently available (*S. o. caurina* vs. *T. alba* assembly statistics include 235 vs. 198 CEGs at least partially present, scaffold N50 of ∼4.0 × 10^6^ nucleotides vs. ∼5.2 × 10^4^ nucleotides, and contig N50 of ∼1.7 × 10^5^ nucleotides vs. ∼1.9 × 10^4^ nucleotides).

The number of annotated genes and the percentage of interspersed repeat elements in our *S. o. caurina* assembly are similar to those seen in other avian genomes (Zhang, Li B, Li C, etal. 2014). The number of annotated genes in our assembly (16,718 genes) was very similar to the number in the high-quality chicken and zebra finch genomes (16,516 and 17,471 genes, respectively) (Zhang, Li B, Li C, etal. 2014). These values were at the upper end of the range seen in the analysis of the gene annotations of 48 avian genomes (13,454–17,471 genes) (Zhang, Li B, Li C, etal. 2014). Similar to the number of annotated genes, the percentage of interspersed repeat elements in our *S. o. caurina* assembly (9.31%) closely matched the percentage found in the chicken and zebra finch genomes (9.82% and 9.68%, respectively) (Zhang, Li B, Li C, etal. 2014). These values fell at the higher end of the range seen in the analysis of 48 avian genomes (4.11–9.82%) if one excludes the downy woodpecker (*P. pubescens*) outlier (22.15%) (Zhang, Li B, Li C, etal. 2014).

Our searches for CEGs with both our CEGMA and BUSCO analyses revealed that our *S. o. caurina* assembly lacks only 5–7% of conserved orthologs, which is similar to the 4.4% we observed to be absent in the assembly of the chicken genome. Genome size data estimated from flow cytometry measurement of red blood cells exist for two *S*. *occidentalis* congeners. The nuclear genome lengths of the tawny owl (*Strix aluco*) and the great gray owl (*S. nebulosa*) are ∼1.56 Gnt (De Vita etal. 1994; Doležel etal. 2003) and 1.61 Gnt (Doležel etal. 2003; Vinogradov 2005), respectively, which average to 1.59 Gnt. As compared with this average, the shorter total length of our scaffolded *S. o. caurina* assembly (∼1.26 Gnt) suggests that 21% of the full genome sequence length of *S. o. caurina* remains unrepresented in this assembly. This is similar to the ∼17.8% unrepresented sequence in the 1.19 Gnt golden eagle genome, assuming a genome size of ∼1.45 Gnt (Doležel etal. 2003; Nakamura etal. 1990). The unrepresented sequence may consist largely of difficult-to-assemble repetitive content (Wicker etal. 2005; Yamada etal. 2004). These data illustrate that the *S. o caurina* assembly is comparable to the top tier of avian genomes assembled to date, but, as with all avian genomes, there is still improvement to be made.

Previous work on *Strix* karyotypes suggests that *S. occidentalis* likely has a typical avian karyotype of 2n = 80–82 (Renzoni and Vegni-Talluri 1966; Hammar 1970; Belterman and Boer 1984; Rebholz etal. 1993;). Assuming 1n = 41 chromosomes, the 8,100 scaffolds in our assembly yield ∼198 scaffolds per chromosome. However, this number may not be a very meaningful estimate of the number of sequence blocks per chromosome as *Strix* shares with other birds the feature of possessing chromosomes in a wide range of sizes with the majority of the karyotype (∼35 of the 41 chromosomes) comprised of microchromosomes and just 6 macrochromosomes (Rebholz etal. 1993).

The SOAPdenovo2 version 2.04 (Luo etal. 2012) assembler does not remove short sequences, which were mostly unincorporated reads. We removed all contigs and scaffolds <1,000 nt for our final assembly and used the resulting assembly in downstream analyses. We felt that removal of these small sequences was warranted as sequences shorter than 1,000 nt are unlikely to be useful in assessing synteny or gene structure. Some commonly used assemblers, such as ALLPATHS-LG, do not output contigs/scaffolds <1,000 nt (Gnerre etal. 2011). Indeed, the authors of the ALLPATHS-LG description removed contigs/scaffolds <1,000 nt in the comparisons of their assembler‘s functionality with other genome assemblers (Gnerre etal. 2011). Removal of these short sequences post assembly allowed us to better compare across assemblies and to effectively analyze what was actually assembled.

Our CEGMA results suggest that we lost minimal genome information (only 1 out of 248 conserved orthologs examined) by removing assembly contigs/scaffolds <1,000 nt. This validated our decision to remove these short sequences and confirmed that it was likely not worth the increase in processing time to retain these small genome fragments in downstream analyses. Additionally, larger genome assembly fragments have greater structural information.

In order to calculate the contig N50 statistic, scaffolds must be decomposed into constituent contigs. We explored how the criteria for splitting scaffolds into contigs affected assembly statistics. As one might expect, allowing longer blocks of N’s before breaking a scaffold into contigs resulted in better continuity statistic values. We chose to allow up to 25 N’s before separating contigs in our final assembly metric calculations as this was the default used in the “assemblathon_stats.pl” script used for calculating assembly statistics of the Assemblathon 2 genome assemblies (Bradnam etal. 2013). Indeed, even though the “assemblathon_stats.pl” script allowed the user to set a flag to change the number of N’s that would separate contigs, our examination of the code revealed that the 25 N’s was actually hard-coded into the script and overrode any value set by the user.

We found that our assemblies had better continuity metrics when we did not include all of our available short read data in the assembly. Of particular benefit was the exclusion of the Hydroshear data set, which displayed a high level of sequence duplication. This suggests that checking libraries for evidence of elevated levels of duplication prior to an assembly could be beneficial.

We found that all of the microsatellite primer pairs previously used for *S. occidentalis* genetic studies (Funk etal. 2007, 2008, 2010) mapped at reasonable distances from each other and predicted PCR products in normal microsatellite size ranges. We found no evidence of linkage except for three primer pairs that mapped to the same scaffold. The other 11 primer sets that we were able to align to the assembly mapped to separate scaffolds. A chromosome-level genomic sequence assembly would help further evaluate the independence of these loci.

### Genome-Wide Divergence of Spotted Owl and Barred Owl

As *S. o. caurina* and *S. varia* are separate species, we expected a high genome-wide *F*_ST_ estimate, but our estimate is elevated even relative to values calculated for other congeneric bird species pairs (Toews etal. 2016). It is difficult to interpret this value; however, as the genome-wide nucleotide diversity within *S. varia* is ∼10-fold greater than that of *S. o. caurina*. We hypothesize that a difference in *N*_e_ for the two species is likely the largest contributor to this difference in nucleotide diversity, especially as the Marin *S. o. caurina* population of which our *S. o. caurina* genome is a sample is known to be an isolated population of this extinction-threatened species (Barrowclough etal. 2005). Following from the 10-fold difference in nucleotide diversity of the two species’ genomes, our PSMC analyses suggested that the *N_e_* of *S. varia* was consistently approximately an order of magnitude greater than that of *S. o. caurina* over the past 100,000 years. The PSMC analyses also suggested that the *N*_e_ of both *S. o. caurina* and *S. varia* has been in decline over the past tens of millennia, but we caution that precise timing of the past maximum *N*_e_ for both species and its subsequent decline is highly dependent on the values chosen for the substitution rate and generation time, which likely require further optimization for these *Strix* species and for owls in general.

### Light-Associated Gene Analyses

We have provided genomic evidence of inactivation and deletion of genes with light-associated functions in two species of predominantly nocturnal owls. Ancestral birds likely possessed tetrachromatic color vision (Borges etal. 2015) characterized by four cone photoreceptor opsin pigments with distinct spectral sensitivities, but it appears that owls have a reduced capacity to discriminate colors. Our genomic data for the color vision system in owls are largely consistent with the results of a retinal microspectrophotometry study (Bowmaker and Martin 1978), retinal transcriptome analyses (Wu etal. 2016), and a recent genomic study of avian visual opsins (Borges etal. 2015). Specifically, the absence of *SWS1*, which absorbs light in the violet/ultraviolet (Davies etal. 2012), in both *S. o. caurina* and *T. alba* is corroborated by the absence of a violet/ultraviolet-sensitive photopigment in *S. aluco* (Bowmaker and Martin 1978), the lack of *SWS1* retinal mRNA transcripts in a tytonid and species from all three of the strigid subfamilies (Wu etal. 2016), and a genomic analysis of *T. alba* that also failed to find *SWS1* in the genome assembly (Borges etal. 2015). In our *S. o. caurina* assembly we were able to locate, albeit on separate scaffolds, the genes that flank *SWS1* in other avian taxa, but not *SWS1* itself. More data is needed to confirm whether there are *SWS1* remnants in the *S. o. caurina* and *T. alba* genomes and their absence in the current assemblies is simply due to assembly incompleteness or errors. However, together the data accumulated to date strongly indicate that owls lack *SWS1*, potentially since their most recent common ancestor, leading to a reduced capacity for color discrimination. The loss of *SWS1* is highly unusual in Aves (Borges etal. 2015). Other than in owls, it has only been inferred to have been lost in the nocturnal North Island brown kiwi (*Apteryx mantelli*) (Le Duc etal. 2015). In contrast, it has occurred repeatedly in nocturnal, subterranean, and marine mammals (Jacobs 2013; Emerling etal. 2015) as well as in the crocodilians, a lineage believed to have undergone an extensive period of nocturnal adaptation (Walls 1942; Emerling 2017a).

The inactivation of *Rh2* in *T. alba* was previously suggested (Borges etal. 2015) and we confirmed this result with the two premature stop codons and two frameshift indels we found in the gene sequence. Additionally, there is evidence that the retinal transcriptome of a congener, *T. longimembris*, does not include *Rh2* transcripts (Wu etal. 2016). The intact copy of *Rh2* in our *S. o. caurina* genome, the transcription of this gene in multiple strigid species (Wu etal. 2016), and the expression of a cone pigment consistent with the Rh2 protein in *S. aluco* (Bowmaker and Martin 1978) all support the hypothesis that *Rh2* was lost uniquely in the tytonid lineage and not across Strigiformes (Wu etal. 2016). Among avian species, *Rh2* is also inactivated in the kiwi *A. mantelli* (Le Duc etal. 2015) as well as in the Adélie (*Pygoscelis adeliae*) and emperor penguins (*Aptenodytes forsteri*) (Li etal. 2014; Borges etal. 2015), two marine predators that frequently feed at great depths under dim-light conditions. A third penguin species, the Humboldt penguin (*Spheniscus humboldti*) lacks cones with a peak absorbance typical of *Rh2* (Bowmaker and Martin 1985). The loss of *Rh2* occurred in several other vertebrate groups that are thought to have experienced long periods of inhabiting dim-light environments, including stem Mammalia (Walls 1942; Davies etal. 2007; Gerkema etal. 2013), Crocodilia (Emerling 2017a), and snakes (Reptilia: Serpentes) (Castoe etal. 2013; Vonk etal. 2013; Simões etal. 2015; Emerling 2017b).

The apparent absence of *SWS2* and *LWS* in *T. alba* is likely due to the assembly being incomplete. These genes are in tandem in *A. carolinensis* and *X. laevis*, but the avian assemblies in Ensembl version 86 (Yates etal. 2016) contain *SWS2* and *LWS* on separate small contigs and not adjacent to other genes. This is consistent with our recovery of only partial *SWS2* and *LWS* in *S. o. caurina* and previous difficulties in assembling full *SWS2* and *LWS* sequences in dozens of other avian genomes (Borges etal. 2015; Le Duc etal. 2015), which may be attributable to the high GC content of these genes (Borges etal. 2015). Researchers recovered intact *SWS2* and *LWS* mRNAs in the retinal transcriptomes of five strigid and one tytonid species (Wu etal. 2016) and have demonstrated that the tawny owl (*S. aluco*) expresses photoreceptor pigments with peak absorptions consistent with *SWS2* and *LWS* (Bowmaker and Martin 1978), suggesting that *SWS2* and *LWS* are likely retained in owls.

Together, the confluence of data from genomics, transcriptomics, and retinal microspectrophotometry suggests that *SWS1* was likely lost in stem Strigiformes, which resulted in a reduction in the degree of color vision from tetrachromacy to trichomacy by the time of the last common ancestor of owls. *Rh2* became subsequently inactivated in Tytonidae, resulting in further reduced capacity for color discrimination (dichromacy) in this family. Owls, kiwis, and penguins represent the few known avian taxa that deviated from the ancestral avian state of tetrachromatic color vision, likely as a result of an increased dependence on highly sensitive rod photoreceptors for foraging in low-light conditions.

The inactivation (*T. alba*) or deletion (*S. o. caurina*) of the gene encoding pinopsin (*OpnP*) may have resulted in the loss of direct photosensitivity of the pineal gland in owls. Pinopsin is expressed in the pineal gland of birds (Okano etal. 1994) and likely regulates the daily rhythms of melatonin synthesis. Owls have a relatively small and simple pineal with little response to differences in luminance (Taniguchi etal. 1993), which suggests that, similar to mammals, the gland may receive photic input indirectly from the eyes (Falcón etal. 2009). *OpnP* is also inactivated in the penguins *P. adeliae* and *A. forsteri* (Li etal. 2014), but it otherwise appears intact across Aves (Borges etal. 2015). Notably, the loss of pinopsin has also occurred in the historically dim-light-environment-inhabiting Mammalia, Crocodilia, and Serpentes (Walls 1942; Gerkema etal. 2013; Emerling 2017a, 2017b). Crocodilians appear to lack a pineal gland entirely (Roth etal. 1980), whereas mammals have a pineal gland that has moved from a more superficial to a deeper position in the brain (Falcón etal. 2009), presumably resulting in a loss of photosensitivity. Together these data suggest that the loss of direct photosensitivity of the pineal gland is a common theme in amniotes (Tetrapoda: Amniota) that experience minimal exposure to light.

Although we found several putative inactivating mutations in *Opn4m*, these are unlikely to have led to complete loss of function. The shared 4 nt mutation in *T. alba* and *S. o. caurina* suggests that it was inherited from the common ancestor of Strigiformes. If this mutation disrupted the function of *Opn4m* in the common ancestor of Strigiformes, then this gene sequence should have been evolving neutrally in all of the descendant lineages. However, Strigidae and Tytonidae split ∼45 million years ago (Prum etal. 2015) yet each ortholog has only accumulated a single additional putative inactivating mutation, both of which are downstream of exon 8. Our dN/dS ratio analyses of crown owl branches yielded an ω < 1 (ω = 0.45), which is consistent with the hypothesis that *Opn4m* remains functional in owls. Furthermore, we were able to assemble *Opn4m* from the retinal mRNA sequences from six additional owls (five strigid and one tytonid), which indicates that *Opn4m* is still being transcribed in the eyes of those species. We found evidence of multiple *Opn4m* isoforms in the avian retinal transcriptome sequences and the genomic sequences of several other avian taxa possessed putative inactivating mutations. These potentially inactivating mutations were almost all distributed on or after exon 8. Notably, when we used the lowest sensitivity setting of the Geneious aligner to map *Opn4m* BLAST hits from the avian retinal transcriptomes, we primarily obtained assembled sequences that terminated after exon 8. Previous work has found multiple *Opn4m* isoforms in vertebrates (Verra etal. 2011; Hughes etal. 2012). Our results suggest loss of some of these isoforms in owls and other birds. *Opn4m* is involved in entraining circadian rhythms in mammals via the pineal gland, in part, as well as in regulating pupil diameter (Hankins etal. 2008). Given the diminished importance of the pineal gland in owls, alteration of the circadian function of *Opn4m* is a possibility.

*CYP2J19* has recently been implicated as the carotenoid ketolase responsible for synthesizing red carotenoids in birds (Lopes etal. 2016; Mundy etal. 2016; Emerling 2017c). Carotenoids, in addition to being involved in pigmentation of avian skin and feathers, are located in oil droplets anterior to the photosensitive outer segments of cone photoreceptors. These oil droplets fine-tune color vision by absorbing shorter wavelengths and reducing spectral overlap between cone visual pigments (Vorobyev 2003). However, these droplets also reduce the number of photons that reach cone photoreceptors and, therefore, may be less beneficial under dim-light conditions. Among owls, *S. aluco*, *Athene noctua* (little owl), and *Asio flammeus* (short-eared owl) are known to possess red cone oil droplets, whereas *Strix uralensis* (Ural owl), *Bubo scandiacus* (snowy owl), and *T. alba* lack them (Erhard 1924; Yew etal. 1977; Bowmaker and Martin 1978; Gondo and Ando 1995). In *S. aluco*, the red oil droplets are limited to <1% of the cone photoreceptor population (Bowmaker and Martin 1978), which is an extremely low proportion compared with other avian species (Bowmaker 1980; Partridge 1989). Additionally, there is recent evidence that *CYP2J19* is inactivated in *T. alba*, is transcribed as a pseudogene in the retinal transcriptome of *Asio otus* (long-eared owl), and is transcribed at low levels in five other owl species as compared with the level observed in diurnal outgroup avian taxa (Emerling 2017c). Among non-owl Aves, the absence of red cone oil droplets has only been reported in two penguin species, *S. humboldti* (Bowmaker and Martin 1985) and *Aptenodytes patagonicus* (Gondo and Ando 1995). Among nonowls, *CYP2J19* is inactivated in the penguins *P. adeliae* and *A. forsteri* as wells as in the kiwi *A. mantelli* (Emerling 2017c), which all forage under dim-light conditions. The *CYP2J19* pseudogene reported here for *S. occidentalis caurina* provides further evidence that owls have repeatedly been losing red carotenoid oil droplets in parallel, potentially to maximize retinal sensitivity in their predominantly nocturnal niche.

Perhaps what is most notable about the loss of light-associated genes in Strigiformes is not the fact that it has occurred, but that it has not ensued to the same extent as in other historically dim-light-adapted vertebrates. Of the nineteen genes we examined, all but one (*CYP2J19*) were likely present in the common ancestor of amniotes (Gerkema etal. 2013; Osborn etal. 2015; Twyman etal. 2016). Excluding *CYP2J19*, mammals lost nine (Mammalia: Marsupialia and Monotremata) to ten of these genes (Mammalia: Placentalia) during a hypothesized nocturnal or mesopic bottleneck (Walls 1942; Heesy and Hall 2010; Davies etal. 2012; Gerkema etal. 2013) and crocodilians lost seven during a similarly hypothesized period of dim-light adaptation (Walls 1942; Emerling 2017a). Among squamates (Reptilia: Squamata), snakes lost seven of these genes during a putative nocturnal and/or fossorial period early in their history, whereas the largely nocturnal geckos lost six (Walls 1942; Emerling 2017b). As for owls, tytonids have lost three of the light-associated genes we examined (*SWS1*, *Rh2*, *OpnP*), whereas strigids have lost only two (*SWS1*, *OpnP*).

## Conclusions

We report the first genome of a member of Strigidae, the largest family of owls. We anticipate that this draft whole genome assembly will be useful to those studying the genetics, demography, and conservation of the spotted owl and related taxa. It will be of particular use in genetic identification of hybrid spotted/barred owls (*S. occcidentalis* × *varia*) and in ascertaining the frequency of hybridization between these two species in the forests of western North America. The phylogenetic position of owls within Neoaves is at the base of a large clade containing mousebirds (Coliiformes), cuckoo-rollers (Leptosomiformes), trogons (Trongoniformes), hornbills (Bucerotiformes), woodpeckers (Piciformes), and kingfishers (Coraciiformes) (Jarvis etal. 2014; Prum etal. 2015). This placement of owls suggests that our spotted owl genome assembly will be useful in genomic studies that span a substantial component of avian morphologic diversity and life history strategies.

Despite potentially more than 45 million years of dim-light specialization in Strigiformes, owls have retained a diverse array of nonvisual opsin pigments and mechanisms to protect against ultraviolet photo-oxidative damage. Although tytonids have a reduced color vision capacity that is similar to ancestral mammals, crocodilians, and snakes, strigids have retained trichromatic color vision akin to that of humans. Many light-associated gene functions have been maintained in owls, perhaps enabling activities during daylight, a time when most owls are presumed to be generally inactive. It appears that what many consider the quintessential nocturnal birds are not as independent of light as are other nocturnal or crepuscular amniote lineages.

## Supplementary Material

Supplementary data are available at *Genome Biology and Evolution* online.

## Supplementary Material

Supplementary Data
