# Supplementary material for: Northern Spotted Owl (Strix occidentalis caurina) Genome: Divergence with the Barred Owl (Strix varia) and Characterization of Light-Associated Genes
Source: Genome Biol Evol. 2017 Aug 23;9(10):2522–45. doi: 10.1093/gbe/evx158 (PMC5629816; doi:10.1093/gbe/evx158)
Supplement: Supplementary Data [file evx158_supp.pdf]

Supplementary Article for:

Northern spotted owl (*Strix occidentalis caurina*) genome: divergence with the barred owl (*Strix varia*) and characterization of light-associated genes

Zachary R. Hanna,[1,2,3,4,*] James B. Henderson,[3,4] Jeffrey D. Wall,[1,3,4,5] Christopher A. Emerling,[1,2] Jérôme Fuchs,[3,6] Charles Runckel,[7,8,9] David P. Mindell,[1] Rauri C. K. Bowie,[1,2] Joseph L. DeRisi,[7,8] John P. Dumbacher[3,4]

[1] Museum of Vertebrate Zoology, University of California, Berkeley, Berkeley, California, United States of America

[2] Department of Integrative Biology, University of California, Berkeley, Berkeley, California, United States of America

[3] Department of Ornithology & Mammalogy, California Academy of Sciences, San Francisco, California, United States of America

[4] Center for Comparative Genomics, California Academy of Sciences, San Francisco, California, United States of America

[5] Institute for Human Genetics, University of California San Francisco, San Francisco, California, United States of America

[6] UMR 7205 Institut de Systématique, Evolution, Biodiversité, CNRS, MNHN, UPMC, EPHE, Sorbonne Universités, Muséum National d'Histoire Naturelle, Paris, France

[7] Department of Biochemistry and Biophysics, University of California San Francisco, San Francisco, California, United States of America

[8] Howard Hughes Medical Institute, Bethesda, Maryland, United States of America

[9] Runckel & Associates, Portland, Oregon, United States of America.

*Author for Correspondence: Zachary R. Hanna, Museum of Vertebrate Zoology, University of California, Berkeley, Berkeley, California, United States of America, zachanna@berkeley.edu

1 Supplementary Material and Methods

1.1 Nextera350nt library

1.1.1 We intended this library to be a Nextera-sheared library with a small insert size. We isolated DNA using a Gentra Puregene Kit (Qiagen) following the protocol entitled "Protocol: DNA Purification from Tissue Using the Gentra Puregene Tissue Kit" (Qiagen). We used 50 ng of the DNA to prepare a genomic library using a Nextera DNA Sample Prep Kit (Illumina-compatible) (Epicentre). After tagmentation, we cleaned the reaction with a DNA Clean & Concentrator -5 kit (Zymo Research). We amplified the reaction for 5 cycles of PCR using a Nextera DNA Sample Prep Kit (Illumina-compatible) (Epicentre) and the Nextera PCR Enzyme (Epicentre). We then cleaned the reaction with a DNA Clean & Concentrator -5 kit (Zymo Research). We used a LabChip XT DNA 750 Assay Kit on a LabChip XT (PerkinElmer) automated nucleic acid fractionation system to select library fragments in the size range of 375-600 nt, which, after subtracting the 141 nt of adapters, corresponds to an average fragment size of 346.5 nt. We performed a final PCR using 5 μL Klentaq LA 10X Buffer with MgCl (Sigma-Aldrich), 1 μL 12.5 μM dNTPs, 1 μL each of two Illumina-adapter-compatible primers at 10 μM, 1 μL KlenTaq LA DNA Polymerase Mix (Sigma-Aldrich), 5 μL library off of LabChip, and water to make a 50 μL reaction volume. We ran the PCR at 94°C for 2 min; then 5 cycles of denaturation at 94°C for 30 s, annealing at 58°C for 30 s, and extension at 72°C for 3 min; and we performed a final extension at 72°C for 5 min. We removed the PCR products after the final extension and and then cleaned them using a DNA Clean & Concentrator -5 kit (Zymo Research). We obtained one lane of 100 nt paired-end data using a TruSeq PE Cluster Kit v2-cBot-HS kit and a TruSeq SBS v2-HS

kit on a HiSeq 2000 (Illumina) and a second lane of 100 nt paired-end data using a TruSeq PE Cluster Kit v3-cBot-HS kit and a TruSeq SBS v3-HS kit on a HiSeq 2000 (Illumina).

1.2 Nextera700nt library

1.2.1 We attempted to construct a Nextera-sheared library with a moderate insert size. We isolated DNA using a Gentra Puregene Kit (Qiagen) and used 50 ng to prepare a genomic library using a Nextera DNA Sample Preparation Kit (Illumina). After tagmentation, we cleaned the reaction with a DNA Clean & Concentrator -5 kit (Zymo Research). We amplified the reaction for 5 cycles of PCR using a KAPA Library Amplification kit (KAPA Biosystems) and then cleaned the reaction with a DNA Clean & Concentrator -5 kit (Zymo Research). We used a BluePippin (Sage Science) to select library fragments in the size range of 734-934 nt, which, after subtracting the 134 nt of adapters, corresponded to selecting an average insert size of 700 nt. We performed a real-time PCR (rtPCR) using a KAPA Real-Time Library Amplification Kit (KAPA Biosystems) on a CFX96 Touch Real-Time PCR Detection System (Bio-Rad) to amplify the library. We amplified the library with 6 cycles PCR and then cleaned the PCR products with a DNA Clean & Concentrator -5 kit (Zymo Research). We lastly assessed the library fragment size distribution with a 2100 BioAnalyzer (Agilent Technologies) and the concentration of double-stranded DNA material with a Qubit 2.0 Flurometer (Invitrogen). We obtained one lane of 150 nt paired-end data sequenced on a HiSeq 2500 (Illumina) in rapid mode.

1.3 Nextera550nt library

1.3.1 We aimed to construct a Nextera-sheared library with overlapping reads, which could be merged into long fragments. We isolated DNA using a Gentra Puregene Kit (Qiagen) and

used 50 ng to prepare a genomic library using a Nextera DNA Sample Preparation Kit (Illumina). After tagmentation, we cleaned the reaction with a DNA Clean & Concentrator -5 kit (Zymo Research). We amplified the reaction for 5 cycles of PCR using a KAPA Library Amplification kit (KAPA Biosystems) and then cleaned the reaction with a DNA Clean & Concentrator -5 kit (Zymo Research). We then used a BluePippin (Sage Science) to select library fragments in the size range of 634-709 nt, which, after subtracting the 134 nt of adapters, corresponded to selecting an average insert size of 537.5 nt. We assessed the library fragment size distribution with a 2100 BioAnalyzer (Agilent Technologies). We cleaned the size-selected product with 0.6X Agencourt AMPure XP (Beckman Coulter) magnetic beads to remove adapter dimer of approximately 250 nt in size. We then performed a real-time PCR (rtPCR) using a KAPA Real-Time Library Amplification Kit (KAPA Biosystems) on a CFX96 Touch Real-Time PCR Detection System (Bio-Rad) to amplify the library. We amplified the library with 8 cycles PCR and then cleaned the PCR products with a DNA Clean & Concentrator -5 kit (Zymo Research). We lastly assessed the library fragment size distribution with a 2100 BioAnalyzer (Agilent Technologies) and the concentration of double-stranded DNA material with a Qubit 2.0 Flurometer (Invitrogen). We obtained one lane of 300 nt paired-end data sequenced using a MiSeq Reagent Kit v3 on a MiSeq (Illumina). We obtained a second lane of 375 nt read 1 and 225 nt read 2 for a total of 600 nt of paired-end read data sequenced using a MiSeq Reagent Kit v3 on a MiSeq (Illumina).

1.4 noPCR550nt library

1.4.1 We extracted genomic DNA from blood using a DNeasy Blood & Tissue Kit (Qiagen). We sheared 4,460 ng genomic DNA in 130 µL in a microTUBE AFA Fiber Pre-Slit

Snap-Cap tube (Covaris) using a M220 focused-ultrasonicator (Covaris) targeting 550 nt as the center of the fragment distribution. We used peak incident power 50 W, 20% duty factor, 200 cycles per burst, and 45 s treatment time at 20°C. We then removed small fragments and concentrated the sheared material using a DNA Clean & Concentrator -5 kit (Zymo Research). We next constructed a genomic library by using a TruSeq DNA PCR-Free Library kit (Illumina) and following the manufacturer's protocol, including the use of bead-based size selection to remove large and small DNA fragments in succession to target a mean fragment size of 550 nt. We assessed the concentration of double-stranded DNA material in the final library with a Qubit 2.0 Flurometer (Invitrogen).

1.5 900ntPCR library

1.5.1 We extracted genomic DNA from blood using a DNeasy Blood & Tissue Kit (Qiagen). We sheared 4,580 ng genomic DNA in 130 μL in a microTUBE AFA Fiber Pre-Slit Snap-Cap tube (Covaris) using a M220 focused-ultrasonicator (Covaris) targeting 900 nt as the center of the fragment distribution. We used peak incident power 50 W, 5% duty factor, 200 cycles per burst, and 70 s treatment time at 20°C. We then removed small fragments and concentrated the sheared material using a DNA Clean & Concentrator -5 kit (Zymo Research). We next constructed a genomic library by using a TruSeq DNA PCR-Free Library kit (Illumina) and following the manufacturer's protocol, except that we only performed a bead-based size selection to remove small fragments and not large fragments. We used a 0.45X bead to sample ratio in order to eliminate fragments smaller than approximately 700 nt. Following A-tailing and prior to adapter ligation, we took 10% of the sample (by volume) and separated it from the noPCR aliquot for use in a PCR-amplified library. We ligated adapters to these two aliquots separately and cleaned

the finished ligations with a DNA Clean & Concentrator -5 kit (Zymo Research). We then only went forward with the aliquot for use in a PCR-amplified library. We used a BluePippin (Sage Science) to select library fragments in the size range of 800-1100 nt, which, after subtracting the 121 nt of adapters, corresponded to selecting an average insert size of 829 nt. We next cleaned the eluted material with a DNA Clean & Concentrator -5 kit (Zymo Research) and then performed real-time PCR (rtPCR) using a KAPA Real-Time Library Amplification Kit (KAPA Biosystems) on a CFX96 Touch Real-Time PCR Detection System (Bio-Rad) to amplify the library. We amplified the library with 11 cycles PCR and then cleaned the PCR products with 1X Agencourt AMPure XP (Beckman Coulter) magnetic beads. We lastly assessed the library fragment size distribution with a 2100 BioAnalyzer (Agilent Technologies) and the concentration of double-stranded DNA material with a Qubit 2.0 Flurometer (Invitrogen).

1.6 Hydroshear library

1.6.1 We isolated DNA using a Gentra Puregene Kit (Qiagen) and used a Hydroshear DNA Shearing Device (GeneMachines) to shear 25 µg in DNA in 100 µL volume with 30 cycles of shearing using speed code 3. We checked the sheared DNA on a 1% agarose gel and saw that fragments had been sheared between 400-1000 nt. We additionally mechanically sheared the DNA by performing 15 passes through a 28 gauge x 1/2 inch needle attached to a 1 cc U-100 Insulin Syringe (Becton, Dickinson and Company). We performed end-repair using 4266 ng sheared DNA in an End-It DNA End-Repair Kit (Epicentre). We incubated the reaction at room temperature for 45 minutes and then inactivated the enzyme by heating to 72°C for 10 minutes followed by cleaning with a DNA Clean & Concentrator -5 kit (Zymo Research). We then added 3’ A tails in a

reaction with 2 μL 10X NEBuffer 2, 0.5 μL 100 mM dATP (Invitrogen), 1 μL Klenow Fragment (3'→5' exo-) (NEB), and 16.5 μL cleaned end-repaired product. We incubated for 45 min at 37°C and then 20 min at 75°C to inactivate the enzyme. We cleaned the reaction with a DNA Clean & Concentrator -5 kit (Zymo Research). We then ligated Illumina-compatible adapters using 1 μL 10X Fast-Link Ligation Buffer (Epicentre), 1 μL 10 mM ATP (Epicentre), 5 μL of end-repaired DNA (0.7835 μg), 2 μL of annealed Illumina-compatible adapters at 10 μM (Integrated DNA Technologies), and 1 μL Fast-Link DNA Ligase (Epicentre) for 10 μL total reaction volume. We incubated the ligation reaction overnight at 16°C and then used 1.5X Agencourt AMPure XP (Beckman Coulter) magnetic beads to clean the ligase reaction and remove any extra adapters. We performed a PCR using 10 μL Klentaq LA 10X Buffer with MgCl (Sigma-Aldrich), 2 μL 12.5 μM dNTPs, 2 μL each of two Illumina-adapter-compatible primers at 10 μM, 2 μL KlenTaq LA DNA Polymerase Mix (Sigma-Aldrich), half of the cleaned ligase reaction in 10 μL, and water to make a 100 μL reaction volume. We ran the PCR in two 50 μL aliquots at 94°C for 5 min; then 2 cycles of denaturation at 94°C for 30 s, annealing at 55°C for 30 s, and extension at 68°C for 3 min; and we performed a final extension at 68°C for 5 min. We removed the PCR products after the final extension and and then cleaned them using a DNA Clean & Concentrator -5 kit (Zymo Research). We used a LabChip XT DNA 750 Assay Kit on a LabChip XT (PerkinElmer) automated nucleic acid fractionation system to select library fragments in the size range of 600-700 nt. We performed a final PCR using 5 μL Klentaq LA 10X Buffer with MgCl (Sigma-Aldrich), 1 μL 12.5 μM dNTPs, 1 μL each of two Illumina-adapter-compatible primers at 10 μM, 1 μL KlenTaq LA DNA Polymerase Mix (Sigma-Aldrich), 5 μL library off of LabChip,

and water to make a 50 μL reaction volume. We ran the PCR at 94°C for 2 min; then 17 cycles of denaturation at 94°C for 30 s, annealing at 58°C for 30 s, and extension at 72°C for 3 min; and we performed a final extension at 72°C for 5 min. We removed the PCR products after the final extension and and then cleaned them using a DNA Clean & Concentrator -5 kit (Zymo Research). We next assessed the library fragment size distribution with a 2100 BioAnalyzer (Agilent Technologies) and the concentration of double-stranded DNA material with a Qubit 2.0 Flurometer (Invitrogen).

1.7 noPCR550nt, 900ntPCR, and Hydroshear libraries

1.7.1 We pooled the barcoded noPCR550nt, 900ntPCR, and Hydroshear libraries equimolarly and we obtained 350 nt read 1 and 250 nt read 2 for a total of 600 nt of paired-end read data from one lane (approximately ⅓ of one lane per library) using a 600-cycle MiSeq Reagent Kit v3 on a MiSeq (Illumina).

1.8 MP4kb, MP7kb, and MP11kb libraries

1.8.1 We constructed and sequenced three large-insert mate-pair libraries. We isolated DNA using a Gentra Puregene Kit (Qiagen) and sent 41.3 μg to GENEWIZ (www.genewiz.com). We requested barcoded mate-pair libraries with insert sizes of 4 kb, 6 kb, and 11 kb constructed using the Nextera Mate Pair Sample Preparation Kit (Illumina). GENEWIZ followed the procedure detailed in the Nextera Mate Pair Sample Preparation Guide (Illumina, Part # 15035209 Rev. C, January 2013). Traces obtained using a 2100 BioAnalyzer (Agilent Technologies) showed the centers of the distributions of the sheared fragmentsthat went into the circularization step of the three mate-pair libraries as 4.2 kb, 7.1 kb, and 10.7 kb. GENEWIZ pooled the three libraries equimolarly

and we obtained one lane (approximately ⅓ of one lane per library) of 100 nt paired-end data sequenced on a HiSeq 2000 (Illumina).

1.9 Trimming - long-insert mate-pair data

1.9.1 We trimmed the Nextera mate-pair data using NxTrim version 0.2.3-alpha (O'Connell 2014; O'Connell et al. 2015), which required BOOST version 1.57.0 (http://www.boost.org). When running NxTrim, we used the "--preserve-mp" flag to prefer mate pair reads as output even if paired-end reads would be longer. NxTrim utilizes the position of the junction identifier sequence in Nextera mate-pair data to classify reads of mate pair libraries as true mate pair reads, paired-end reads, or singleton reads.

1.9.2 We trimmed adapters and low quality bases separately for the resulting mate-pair data, paired-end reads, and singleton reads using Trimmomatic version 0.32 (Bolger et al. 2014). We trimmed adapters using options "ILLUMINACLIP:<fasta of Illumina adapter sequences >:2:30:10". We removed low quality bases from the beginning and end of the reads using the following options: LEADING:3 TRAILING:3 to remove bases below Phred 3. We trimmed off low quality sequence portions using: SLIDINGWINDOW:4:17, which trimmed the read when the average quality over 4 basepairs dropped below Phred 17. Finally, we trimmed reads less than 36 basepairs in length using "MINLEN:36".

1.10 Trimming - short-insert paired-end data

1.10.1 We first trimmed adapters from all non-mate-pair libraries using Trimmomatic version 0.32 (Bolger et al. 2014). We used the ILLUMINACLIP function with the following options: <fasta of Illumina adapter sequences >:2:30:10.

1.10.2 Since substantial portions of the paired-end reads from all of the libraries, except the Nextera700nt library were overlapping, we joined overlapping paired reads using the BBMerge tool in the BBMap tool suite version 34.00 (Bushnell 2014). We merged overlapping reads using the options "minoverlapinsert=110 mininsert=110 strict=t" for the datasets Nextera350nt lane 1 and Nextera350nt lane 2, We used the options "minoverlapinsert=400 mininsert=400 strict=t" for the datasets Nextera550nt lane 1, Nextera550nt lane 2, noPCR550nt, and PCR900nt, which had longer read lengths.

1.10.3 We then performed quality trimming using Trimmomatic version 0.32 (Bolger et al. 2014). We removed low quality bases from the beginning and end of the reads using the options “LEADING:3 TRAILING:3” to remove bases below Phred 3. We trimmed off low quality sequence portions using “SLIDINGWINDOW:4:17”, which trimmed the read when the average quality over 4 basepairs dropped below Phred 17. Finally, we trimmed reads less than 36 basepairs in length using “MINLEN:36”.

1.11 Error-correction

1.11.1 Since we trimmed using a moderately low quality threshold, we used the k-mer-based error corrector in the SOAPdenovo2 toolkit, SOAPec version 2.01 (Luo et al. 2012), to correct sequence errors. We first used the KmerFreq_HA tool to create a k-mer frequency spectrum with default options except “-k 27 -L 600”, which indicate that we used a k-mer size of 27 for creating the frequency spectrum and the maximum read length was 600 nt. We then used the Corrector_HA tool along with the k-mer frequency spectrum that we created to correct all of our trimmed reads using default options except “-k 27 -r 36”, which indicate that we used a k-mer size of 27 for the error correction and kept trimmed reads as short as 36 nt.

1.12 Single-end data

1.12.1 In each stage of the trimming, merging, and error-correction process, some reads previously paired became unpaired due to the loss of their paired read in a trimming step. We handled the single-end reads separate from the paired reads and subjected them to the same adapter, quality trimming, and error-correcting steps as the reads that remained paired. We used all of these single read sets in the final assembly.

1.13 Read processing variation for some preliminary assemblies

1.13.1 For a trim level of an average Phred 7 or 28, the only difference from the methodology described above was that we trimmed off low quality sequence portions using Trimmomatic with the parameter "SLIDINGWINDOW:4:7" or "SLIDINGWINDOW:4:28", respectively.

1.13.2 We did not apply the error-correction process to reads trimmed to an average Phred 28.

1.13.3 For some preliminary assemblies, we did not merge overlapping paired-end reads. This entailed leaving out the BBMerge step described above, but still performing adapter and quality trimming as noted.

1.14 Genome size

1.14.1 Genome size data estimated from flow cytometry measurement of red blood cells exist for two *S. occidentalis* congeners of, *S. aluco* and *S. nebulosa*. *Strix aluco* has a C-value of 1.59 pg (De Vita et al. 1994), which is approximately 1.56 Gnt (Doležel et al. 2003). *Strix nebulosa* has a C-value of 1.65 pg (Vinogradov 2005), which is approximately 1.61 Gnt (Doležel et al. 2003).

1.14.2 We ran Preqc (Simpson 2014), a module within SGA version 0.10.14 (Simpson & Durbin 2010, 2016), which used Google SparseHash library version 2.0.2 (google-

sparsehash@googlegroups.com 2012), zlib version 1.2.8 (Gailly & Adler 2013) and BamTools version 2.4.0 (Barnett et al. 2011, 2015) requiring CMake version 3.2.3 (Hoffman & Martin 2003; Kitware 2015), and on the 150 nt paired-end reads from the Nextera700nt dataset to estimate the genome size. Preqc estimated the genome size by sampling 20,000 reads and counting the frequency of *k*-mers of length 31 nt while applying a correction for sequencing errors.

1.15 Assembly

1.15.1 We used SOAPdenovo2 version 2.04 (Luo et al. 2012) to assemble the genome. We performed numerous trial runs experimenting with different k-mer values and parameters. We utilized the insert size estimated in the output of initial, trial assemblies to refine our estimation of the insert sizes for our libraries and used these refined values as input into subsequent assembly configuration files (Table S1). We settled on using the default parameters other than the options "SOAPdenovo-127mer all -N 1500000000 -K 23 -m 127 -k 65 -d 1 -R -F". These options indicate that we used the 127 k-mer version of the assembler and ran the assembly using multiple k-mer sizes starting at 23 and ending with a maximum of 127, we gave an estimated genome size of 1.5 Gnt, we allowed reads as small as 65 nt to map to contigs during scaffolding, we ignored singleton k-mers, we tried to resolve repeats with reads, and we attempted to fill gaps in scaffolds.

1.15.2 In our configuration files for all of the preliminary assemblies, we used the default minimum alignment lengths between a read and contig (32 for paired-end reads, 35 for mate-pair reads) and the default minimum pair number cutoffs (3 for paired-end reads, 5 for mate-pair reads).

1.15.3 We used dupchk (Henderson & Hanna 2016a), which utilized the first and last 21 nt of each read as a read fingerprint, to check for sequence duplication in each sequenced library.

1.16 Preliminary assembly assessment

1.16.1 In order to compare our preliminary assemblies, we removed contigs / scaffolds <= 300 nt in order to remove any unassembled reads from the assembly. We calculated the contig and scaffold N50 as well as the number of scaffolds in various length classes using scafN50 (Henderson & Hanna 2016d). We calculated the total length of the assembly, the % Ns, and the total number of scaffolds using scafSeqContigInfo (Henderson & Hanna 2016e). We were conservative and separated scaffolds into contigs at each N in the sequence, which is the default option for scafSeqContigInfo (Henderson & Hanna 2016e).

1.16.2 We then used CEGMA version 2.5 (Parra et al. 2007), which required GeneWise from the Wise2 version 2.2.3-rc7 package (Birney; Birney et al. 2004), HMMER version 3.0 (http://hmmer.org), geneid version 1.4.4 (Guigó 1998; Blanco et al. 2011), and NCBI's BLAST+ version 2.2.25 (Altschul et al. 1997; Camacho et al. 2009), to annotate a set of highly conserved eukaryotic genes in our assembly and thereby obtain an assessment of the quality and completeness of each assembly. In order to install CEGMA's GeneWise dependency, we followed the source code modification recommendations documented by Markus Grohme (http://korflab.ucdavis.edu/datasets/cegma/ubuntu_instructions_1.txt) and the Homebrew Science GeneWise formula (https://github.com/Homebrew/homebrew-science/blob/master/genewise.rb).

1.17 Determination of final assembly

1.17.1 We examined multiple statistics in choosing our final assembly. We valued high contig and scaffold N50 values, low % Ns in the sequence, a low total number of scaffolds, larger numbers of long scaffolds, and completeness as reflected in the number of conserved genes found by the CEGMA pipeline. We decided that the assembly that had the best statistics across these categories was assembly 4 (Table 2) and we went forward with this assembly as our final assembly.

1.18 Gap closing

1.18.1 We found that using the "-F" flag to fill gaps using the SOAPdenovo2 version 2.04 (Luo et al. 2012) *de novo* assembler was ineffective at gap filling during the assembly. We then filled gaps using the gap closing tool in the SOAPdenovo2 toolkit, GapCloser version 1.12-r6 (Luo et al. 2012), with the default options other than "-l 600" to specify that our longest read length was 600 nt. The program output a warning stating that the maximum supported read length was 155 nt and that it would use that setting for the analysis. We assumed that the program just used the first 155 nt of reads with a total length exceeding 155 nt.

1.18.2 The gap-closed assembly contained many contigs and/or scaffolds under 1000 nt in length, a substantial portion of which appeared to be unassembled reads. We used ScaffSplitN50s (Henderson & Hanna 2016c) to compare the continuity statistics resulting after removing contigs / scaffolds of lengths 300, 500, and 1,000 nt as well as when using N blocks of lengths 1, 5, 10, 15, 20, and 25 to separate contigs within scaffolds. Based on these results, we removed all contigs and scaffolds less than 1000 nt for downstream analyses.

1.19 Final assembly stats

1.19.1 We used CEGMA version 2.5 (Parra et al. 2007), which required GeneWise from the Wise2 version 2.2.3-rc7 package (Birney; Birney et al. 2004), HMMER version 3.0 (http://hmmer.org), geneid version 1.4.4 (Guigó 1998; Blanco et al. 2011), and NCBI's BLAST+ version 2.3.0 (Altschul et al. 1997; Camacho et al. 2009), to annotate a set of highly conserved eukaryotic genes in our assembly and thereby obtain an assessment of the quality and completeness of the assembly. We ran CEGMA with default parameters other than specifying "--vrt" to optimize the searches for a vertebrate genome.

1.19.2 We used BUSCO version 1.1b1 (Simão et al. 2015a; Simão et al. 2015b), which used NCBI's BLAST+ version 2.2.28 (Altschul et al. 1997; Camacho et al. 2009), HMMER version 3.1b2 (http://hmmer.org), and AUGUSTUS version 3.2.1 (Keller et al. 2011; Stanke 2015) to assess the assembly quality by searching for conserved orthologs. We ran BUSCO with default genome mode parameters other than specifying "vertebrata" as the evolutionary lineage with the option "-l" and using "-sp chicken" to employ the AUGUSTUS parameters optimized for the chicken genome.

1.20 Contamination assessment

1.20.1 We performed a local alignment of all scaffolds in NSO-wgs-v0 to a copy the NCBI nucleotide database (nt) that we downloaded on 24 June 2016 (Clark et al. 2016; NCBI Resource Coordinators 2016) using NCBI's BLAST+ version 2.3.0 tool BLASTN (Altschul et al. 1997; Camacho et al. 2009) with default parameters other than "-outfmt 10 -num_alignments 5 -max_hsps 1". We used these parameters to limit to 5 the maximum number of alignments to unique subjects output and to limit to 1 the number of outputted alignments per subject. This allowed us to examine the top 5 alignments to

different subject sequences and ascertain whether those subject sequences were obtained from vertebrate or non-vertebrate organisms.

1.20.2 In order to parse the taxonomy of the subject sequences in the alignment output, we obtained the a local copy of the NCBI taxonomy database using NCBI's BLAST+ version 2.3.0 script, update_blastdb.pl with the parameters "--passive --timeout 300 --force --verbose taxdb". We also downloaded the files taxdump.tar.gz and gi_taxid_nucl.dmp.gz from NCBI (ftp://ftp.ncbi.nlm.nih.gov/pub/taxonomy) (Clark et al. 2016; NCBI Resource Coordinators 2016). We then used GItaxidIsVert (Henderson & Hanna 2016b) with default options other than using the parameter "-n" to filter the alignment output for non-vertebrate alignments.

1.20.3 We used the web version of NCBI's BLAST+ version 2.4.0 tool BLASTN (Altschul et al. 1997; Camacho et al. 2009) with default parameters.

1.21 Mitochondrial genome identification

1.21.1 We searched NSO-wgs-v1 (not repeat-masked, all contigs / scaffolds < 1,000 nt removed, contaminant scaffolds removed) for any of the contigs / scaffolds that were assemblies of the mitochondrial genome, rather than the nuclear genome using NCBI's BLAST+ version 2.4.0 tool BLASTN (Altschul et al. 1997; Camacho et al. 2009) with default parameters other than "-outfmt 6".

1.21.2 We annotated the scaffold using the MITOS WebServer version 806 (Bernt et al. 2013) and specifying "genetic code = 02 - Vertebrate" with default settings otherwise.

1.22 Sex identification

1.22.1 We searched NSO-wgs-v1 for matches to *S. varia CHD1W* and *CHD1Z* nucelotide sequences using NCBI's BLAST+ version 2.4.0 tool BLASTN (Altschul et al. 1997; Camacho et al. 2009) with default parameters other than "-outfmt 6".

1.22.2 We used the Geneious version 9.1.4 aligner through the "map to reference" function (Kearse et al. 2012; Biomatters 2016a) with default options to align primers 2550F and 2718R (Fridolfsson & Ellegren 1999) to the scaffolds and then extract the region bounded by the aligned primers.

1.23 Repeat annotation

1.23.1 We performed a homology-based repeat annotation of the genome assembly using RepeatMasker version 4.0.5 (Smit et al. 2013), which employs the repeat databases of the DFAM library version 1.3 (Wheeler et al. 2013) and the Repbase-derived RepeatMasker libraries version 20140131 (Jurka 1998, 2000; Jurka et al. 2005; Bao et al. 2015). Our installation of the RepeatMasker tool utilized NCBI's BLAST+ version 2.2.30 (Altschul et al. 1997; Camacho et al. 2009) and RMBlast version 2.2.28 (Smit et al. 2015) sequence search engines as well as the tandem repeats finder (TRF) version 4.0.7b (Benson 1999, 2012). We ran RepeatMasker with default options other than parameters "-gccalc -nolow -species aves". The purpose of this run was to produce a masked genome without masking of low complexity regions or simple repeats, which we could then use for downstream annotation steps.

1.23.2 We performed a *de novo* modeling of the repeat elements in the genome using RepeatModeler version 1.0.8 (Smit & Hubley 2015), which uses two *de novo* repeat finders, RECON version 1.08 (Bao & Eddy 2002) and RepeatScout version 1.0.5 (Price et al. 2005), as well as the tandem repeats finder (TRF) version 4.0.7b (Benson 1999,

2012), the RMBlast version 2.2.28 (Smit et al. 2015) sequence search engine, and RepeatMasker version 4.0.5 {Smit et al., 2015} with Repbase-derived RepeatMasker libraries version 20140131 (Jurka 1998, 2000; Jurka et al. 2005; Bao et al. 2015). We built a sequence database from our genome and ran RepeatModeler with default options.

1.23.3 We further masked the genome by running RepeatMasker again with the masked genome as input, using the repeat database created by our RepeatModeler run, and with default options other than parameters "-gccalc -nolow”.

1.23.4 We performed homology-based repeat masking using RepeatMasker as above with default options other than parameters "-gccalc -species aves”. We then performed a second run of RepeatMasker using the repeat database created by our RepeatModeler run with the masked genome as input and using default options other than parameters "-gccalc -nolow”. Our output was a second twice-masked genome with masked low complexity regions and simple repeats.

1.24 Gene annotation

1.24.1 We used the MAKER accessory script, cegma2zff, to convert the GFF file output from our CEGMA run on the GapClosed assembly into ZFF format to use in training of the gene prediction tool Semi-HMM-based Nucleic Acid Parser (SNAP) version 2006-07-28 (Korf 2004). We used the fathom tool of the SNAP package with the parameters “-categorize 1000”, followed by fathom with the parameters “-export 1000”, then the forge element of the SNAP package, then the hmm-assembler.pl script from the SNAP package to convert the ZFF files to an HMM file, which was then the newly trained gene finder that we provided SNAP in the MAKER configuration file (Campbell et al. 2014).

1.24.2 We ran MAKER using NCBI's BLAST version 2.2.31+ (Altschul et al. 1997; Camacho et al. 2009); the sequence comparison tool, exonerate version 2.2.0 (Slater & Birney 2005) with glib version 2.46.2; and the gene prediction tool, AUGUSTUS version 3.2.1 (Keller et al. 2011) for which we specified "chicken" for the gene prediction species model. We employed default parameters for all BLAST and exonerate statistics thresholds and default parameters for all other MAKER configuration options. We used Open MPI version 1.10.2 (Gabriel et al. 2004) to run MAKER on 62 cores for 50.62 hours.

1.24.3 We combined the annotations for all of the genes using the MAKER accessory scripts "fasta_merge" and "gff3_merge" with default options.

1.24.4 We assigned putative gene functions to the MAKER annotations by first obtaining the Uniprot manually annotated and non-redundant protein sequence database Swiss-Prot UniProt release 2016_04 (Consortium 2015) on 2016 April 25 and indexing it using NCBI's BLAST version 2.2.31+ (Altschul et al. 1997; Camacho et al. 2009) tool "makeblastdb" with default parameters other than the options "-input_type fasta -dbtype prot". We then compared the combined MAKER protein fasta file to the Swiss-Prot UniProt database using the BLAST 2.2.31+ tool "blastp" with default parameters other than the options "-evalue .000001 -outfmt 6 -num_alignments 1 -seg yes -soft_masking true -lcase_masking -max_hsps 1". We then used the MAKER accessory script "maker_functional_gff" to add the protein homology data to the combined MAKER GFF3 file and the MAKER accessory script "maker_functional_fasta" to add the protein homology data to the combined MAKER protein and transcript fasta files.

1.24.5 In order to identify proteins with known functional domains, we ran InterProScan version 5.18-57.0 (Jones et al. 2014) with options “-appl PfamA -iprlookup -goterms -f tsv”, which limited searches to Pfam, a database of protein family domains, on the protein sequences generated by MAKER. We then used the MAKER accessory script “ipr_update_gff” to update the MAKER-generated GFF3 file with the results of the InterProScan run and add information on protein family domain matches.

1.24.6 We then filtered transcripts with an Annotation Edit Distance (AED) less than 1 and/or a match to a Pfam domain using the option “-s” in the script “quality_filter.pl” supplied in MAKER version 3.00.0 (Cantarel et al. 2008).

1.24.7 We used the “stat” tool of GenomeTools version 1.5.1 (Gremme et al. 2013) to calculate annotation summary statistics, including distributions of gene lengths, exon lengths, number of exons per gene, and coding DNA sequence (CDS) lengths (measured in amino acids). We also used the “stat” tool of GenomeTools with the options “-addintrons” and “-intronlengthdistri” to infer intron lengths within the annotated gene boundaries and calculate the distribution of intron lengths.

1.25 Alignment

1.25.1 We aligned each set of reads to NSO-wgs-v1-masked using bwa version 0.7.12-r1044 (Li 2013a) with default options other than parameters "bwa mem -M". We separately aligned paired-end and unpaired reads. For alignment of the paired-end data, we set the insert size to be equal to our estimates from our initial assemblies. We set the parameter "-w" to be equal to twice the standard deviation of the insert size we estimated from our initial assemblies.

1.25.2 We merged the paired-end and unpaired read alignments using the Picard version 1.104 function MergeSamFiles (http://broadinstitute.github.io/picard) and sorted them using the Picard version 1.104 function SortSam (http://broadinstitute.github.io/picard), employing default settings for both tools. We next marked duplicate reads (both PCR and optical) using the Picard version 1.104 function MarkDuplicates (http://broadinstitute.github.io/picard), employing default settings.

1.25.3 We assessed the genome coverage, duplication level, and other statistics of each read set based on the read alignments. We used the Picard version 1.141 function CollectWgsMetrics (http://broadinstitute.github.io/picard) with the bam file output by MarkDuplicates as the input file, employing default settings, except setting COUNT_UNPAIRED=True to include coverage contributed by unpaired reads when calculating the alignment statistics. The default CollectWgsMetrics settings included setting the minimum mapping quality for a read to contribute coverage as 20 and the minimum base quality for a base to contribute coverage as 20. We also ran CollectWgsMetrics with the default settings and COUNT_UNPAIRED=False to obtain the portion of the total aligned reads contributed by unpaired reads.

1.25.4 In order to obtain an estimate of the insert size of the mate pair libraries independent of the N-gaps in the scaffold sequences, we divided the scaffolds into contigs at 25 or more N's using make-contig-ref.sh from NSO-genome-scripts version 1.0.0 (Hanna & Henderson 2017) with bioawk version 1.0 (Li 2013b), GNU Awk (GAWK) version 4.0.1 (Free Software Foundation 2012), and GNU fold version 8.21 (MacKenzie 2013). We then aligned the mate pair libraries to this set of contigs using bwa version 0.7.10-r789 (Li 2013a) with default options other than parameters "bwa mem -M". For alignment of

the paired-end data, we set the insert size to be equal to our estimates from our initial assemblies. We set the parameter "-w" to be equal to twice the standard deviation of the insert size we estimated from our initial assemblies. We calculated the insert sizes for each of the three mate pair libraries from these alignments using calcInsertLen.sh from NSO-genome-scripts version 1.0.0 (Hanna & Henderson 2017) with bioawk version 1.0 (Li 2013b).

1.26 Microsatellite analysis

1.26.1 We searched the assembly for 16 pairs of microsatellite primer sequences using NCBI's BLAST+ version 2.4.0 tool BLASTN (Altschul et al. 1997; Camacho et al. 2009) with default parameters other than "-outfmt 6 –word_size 7".

1.27 Barred owl divergence

1.27.1 We used 50 ng genomic DNA to prepare a whole-genome library using a Nextera DNA Sample Preparation Kit (Illumina). After tagmentation, we cleaned the reaction with a DNA Clean & Concentrator -5 kit (Zymo Research). We amplified the reaction with 5 cycles of PCR using a KAPA Library Amplification kit (KAPA Biosystems) and then cleaned the reaction with a DNA Clean & Concentrator -5 kit (Zymo Research). We used a BluePippin (Sage Science) to select library fragments in the size range of 500-700 nt, which, after subtracting the 134 nt of adapters, corresponded to selecting an average insert size of 466 nt. We cleaned the BluePippin products with 0.6X Agencourt AMPure XP (Beckman Coulter) magnetic beads and then performed a real-time PCR (rtPCR) using a KAPA Real-Time Library Amplification Kit (KAPA Biosystems) on a CFX96 Touch Real-Time PCR Detection System (Bio-Rad) to amplify the library with 8 cycles PCR. We then cleaned the PCR products with a DNA Clean & Concentrator -5 kit (Zymo

Research). We lastly assessed the library fragment size distribution with a 2100 BioAnalyzer (Agilent Technologies) and the concentration of double-stranded DNA material with a Qubit 2.0 Flurometer (Invitrogen). We combined this library with others and sequenced it on two successive runs of 150 nt paired-end sequencing using a 2-lane flow cell on a HiSeq 2500 (Illumina) in rapid mode. On the first run, we obtained sequencing data from a portion of each of the two flow cell lanes. On the second run, we obtained data from a portion of one of the two flow cell lanes. We combined all of the data from the two runs for the downstream steps.

1.27.2 We performed adapter and quality trimming of the sequence data using Trimmomatic version 0.32 (Bolger et al. 2014). We used the following options: "ILLUMINACLIP:<fasta of Illumina adapter sequences>:2:30:10 LEADING:3 TRAILING:3 SLIDINGWINDOW:4:28 MINLEN:36".

1.27.3 We aligned trimmed paired and unpaired reads to NSO-wgs-v1-masked using bwa mem version 0.7.12-r1044 (Li 2013a) with default options other than parameters "bwa mem -M". We separately aligned paired-end and unpaired reads. For alignment of the paired-end reads, we set the insert size to be equal to the size estimate of the final library given by the 2100 BioAnalyzer (Agilent Technologies) minus the length of the adapters, which gave an insert size of 466 nt. Additionally, for the alignment of the paired-end reads we set the parameter "-w", the maximum insert size, equal to 1000.

1.27.4 We merged the paired-end and unpaired sequence alignments using the Picard version 1.104 function MergeSamFiles (http://broadinstitute.github.io/picard) and sorted them using the Picard version 1.104 function SortSam (http://broadinstitute.github.io/picard), employing default settings for both tools. We next marked duplicate sequences (both

PCR and optical) using the Picard version 1.104 function MarkDuplicates (http://broadinstitute.github.io/picard), employing default settings.

1.27.5 We calculated various alignment statistics using the Picard version 1.141 function CollectWgsMetrics (http://broadinstitute.github.io/picard) with the bam file output by MarkDuplicates as input and employing default settings except setting COUNT_UNPAIRED=True in order to include coverage contributed by unpaired reads in the calculation of the statistics on the aligned reads. The default CollectWgsMetrics settings include setting the minimum mapping quality for a read to contribute coverage as 20 and the minimum base quality for a base to contribute coverage as 20. We also ran CollectWgsMetrics with the default settings and COUNT_UNPAIRED=False to obtain the portion of the total aligned reads contributed by unpaired reads.

1.27.6 We used Genome Analysis Toolkit (GATK) version 3.4-46 UnifiedGenotyper (McKenna et al. 2010; DePristo et al. 2011; Van der Auwera et al. 2013) to call SNPs using the *S. occidentalis* (Sequoia) and *S. varia* (CNHM<USA-OH>:ORNITH:B41533) bwa-aligned, sorted, duplicate-marked bam files as simultaneous inputs and employing default options other than setting "--output_mode EMIT_ALL_SITES".

1.27.7 We first filtered the variant file using the following GNU Awk (GAWK) version 4.0.1 (Free Software Foundation 2012) command: “awk 'NF==11 && substr($1, 1, 2) != "##" && $6>=50 && $1 != "#CHROM" && $1 != "C7961234" && $1 != "C7963448" && $1 != "C7970814" && $1 != "C8091874" && $1 != "scaffold3674"' | awk '$4=="A" || $4=="C" || $4=="G" || $4=="T"' | awk '$5=="A" || $5=="C" || $5=="G" || $5=="T"' > filtered1.vcf”. This removed lines without 11 fields, header lines, variant sites where the

Phred-scaled probability that a polymorphism exists was < 50, contaminant scaffolds, the mitochondrial genome scaffold, indels, and non-polymorphic sites.

1.27.8 We then calculated the unfiltered allele depth (the number of reads that supported an allele) summed across all of the alleles at each of the remaining variant sites using the following GNU cut version 8.21 (Ihnat et al. 2013) and GNU Awk (GAWK) version 4.0.1 (Free Software Foundation 2012) command: "cat filtered1.vcf | cut -f10,11 | awk 'BEGIN {cov} {split($1,a,":"); split(a[2],acov,","); split($2,b,":"); split(b[2],bcov,","); totcov = acov[1]+acov[2]+bcov[1]+bcov[2]; print totcov}' > vcf-coverage.out". We then graphed these depths and calculated the mean and standard deviation (σ) of the distribution using vcf-coverage-calc.py from NSO-genome-scripts version 1.0.0 (Hanna & Henderson 2017) with Python version 2.7.12 (Python Software Foundation 2016), matplotlib version 1.5.1 (Hunter 2007; Matplotlib Development Team 2016), and NumPy version 1.11.1 (NumPy Developers 2016).

1.27.9 When calculating the nucleotide diversity both within and between samples (H_w and H_b), we removed variants where the unfiltered allele depth summed across all of the alleles was greater than 5σ greater than the mean depth, variants without information for both samples, and variants where the *S. o. caurina* genotype was homozygous for the non-reference allele. We used calc-pi-exclude-onlySPOW.sh and calc-pi-exclude-onlyBADO.sh from NSO-genome-scripts version 1.0.0 (Hanna & Henderson 2017) with GNU cut version 8.21 (Ihnat et al. 2013) and GNU Awk (GAWK) version 4.0.1 (Free Software Foundation 2012) to calculate the H_w for *S. o. caurina* and *S. varia*, respectively. We used calc-pi-exclude.sh from NSO-genome-scripts version 1.0.0 (Hanna & Henderson 2017) with GNU Awk (GAWK) version 4.0.1 (Free Software Foundation

2012) to calculate H_b for *S. o. caurina* and *S. varia*. In order to report H_w and H_b in terms of the number of nucleotide differences per site within the sample, we divided the output from the scripts above by the number of ACGT characters in NSO-wgs-v1-nuc (the whole-genome assembly without the contaminant or mitochondrial scaffolds), which we obtained using "assemblathon-stats-ex.pl" from NSO-genome-scripts (Bradnam et al. 2013; Hanna & Henderson 2017).

1.27.10 We averaged the values of H_w for *S. o. caurina* and *S. varia* and then used this average along with H_b in equation 3 from a study by Hudson, Slatkin & Maddison (1992) in order to estimate F_{ST} between *S. o. caurina* and *S. varia*.

1.28 PSMC analysis

1.28.1 In order to prepare our data for input into an analysis using an implementation of the pairwise sequentially Markovian coalescent model, PSMC version 0.6.5-r67 (Li 2015; Li & Durbin 2011), we used Samtools version 1.3.1 with HTSlib 1.3.1 (Li, Handsaker, Marshall, et al. 2016; Li et al. 2009), bcftools version 1.3.1 (Li, Handsaker, Danecek, et al. 2016), and the vcfutils.pl script from bcftools to call variants with the command "samtools mpileup -C50 –uf reference-genome.fa alignment-file.bam | bcftools call -c - | vcfutils.pl vcf2fq -d minimum-read-depth -D maximum-read-depth | gzip >variants.fq.gz". As per the recommendation of the PSMC documentation (https://github.com/lh3/psmc), we used a third of the average read depth as the minimum read depth (-d) and twice the average read depth as the maximum read depth (-D) (-d 20 -D 126 and -d5 -D 33 for *S. o. caurina* and *S. varia*, respectively). We determined the average read depth using Samtools version 1.3.1 with HTSlib 1.3.1 (Li, Handsaker, Marshall, et al. 2016; Li et al. 2009) and GNU Awk (GAWK) version 4.0.1 (Free

Software Foundation 2012) with the command “samtools depth alignment-file.bam | awk '{sum += $3} END {print sum / NR}'”.

1.28.2 After variant calling, we used the PSMC script “fq2psmcfa” next with the command “fq2psmcfa -q20 variants.fq.gz >variants.psmcfa”. We then ran PSMC with the command “psmc -N25 -t15 -r5 -p "4+25*2+4+6" -o variants.psmc variants.psmcfa”. We next ran the PSMC scripts “psmc2history.pl” and “history2ms.pl” with the command “psmc2history.pl variants.psmc | history2ms.pl > variants.psmc_ms-cmd.sh”.

1.28.3 We ran 100 rounds of bootstraping by first splitting long reference sequences into shorter lengths in the variants.psmcfa file using the PSMC script “splitfa” with the command “splitfa variants.psmcfa >variants-split.psmcfa” and then running PSMC with the command “parallel -j25 ‘psmc -N25 -t15 -r5 -b -p "4+25*2+4+6" -o variants-split-round-{}.psmc variants-split.psmcfa’ :::: <(seq 100)”.

1.28.4 We graphed the output of our PSMC run and rounds of bootstrapping by first combining using GNU cat version 8.21 (Granlund & Stallman 2013) with the command “cat variants.psmc variants-split-round-*.psmc >variants-combined.psmc”. We then plotted the output using the PSMC script “psmc_plot.pl” with the command “psmc_plot.pl -u 4.6e-09 -g 2 variants-combined-plot variants-combined.psmc”. We used 2 years as the generation time (-g option for psmc_plot.pl) for both *S. o. caurina* and *S. varia* (Gutiérrez et al. 1995; Mazur & James 2000) although *S. o. caurina* may breed in its first year (Hamer et al. 1994) and some researchers have estimated the generation time *S. o. caurina* as 10 years (Noon & Biles 1990; U.S. Forest Service 1992). We used 4.6×10^{-9} mutations per site per generation (Smeds et al. 2016) as the mutation rate (-u option for psmc_plot.pl).

1.29 Light-associated gene analyses

1.29.1 We searched in NSO-wgs-v1 for regions orthologous to probes for 19 genes that encode proteins with light-associated functions using Geneious version 9.1.6 (Biomatters 2016b; Kearse et al. 2012) and the included version of the NCBI BLAST+ BLASTn tool (Zhang et al. 2000) with default options. On 1-10 November, 2016, we used the web version of NCBI BLAST+ version 2.5.0 (Zhang et al. 2000) with discontiguous megablast options to align the probes against sequences in the NCBI Whole-Genome-Shotgun (WGS) contigs database limited by specifying the organism *T. alba* (taxid:56313).

1.29.2 When BLAST searches were unsuccessful, we used synteny data from Ensembl (version 86; (Yates et al. 2016) to search for evidence of whole gene deletion. We identified genes flanking the gene of interest in related taxa, and subsequently used BLAST to align the reference sequences for these genes against the *S. o. caurina* and *T. alba* genome assemblies. We imported the *S. o. caurina* genome assembly into Geneious version 9.1.6 (Biomatters 2016b; Kearse et al. 2012) and used the included version of the NCBI BLAST+ BLASTn tool (Zhang et al. 2000) to search for the flanking genes in our assembly. We used the web version of NCBI BLAST+ version 2.5.0 (Zhang et al. 2000) to align the flanking genes against *T. alba* sequences in the NCBI Whole-Genome-Shotgun (WGS) contigs database.

1.29.3 We used the NCBI BLAST+ version 2.5.0 blastn tool (Zhang et al. 2000) with the discontiguous megablast option to align a reference *Opn4m* sequence to fifteen avian retinal transcriptomes in NCBI's Sequence Read Archive (SRA) (Leinonen et al. 2011; NCBI Resource Coordinators 2016) including the pied harrier (*Circus melanoleucos*) (SRA accession SRR3203217), long-eared owl (*Asio otus*) (SRA accession

SRR3203220), eastern grass owl (*Tyto longimembris*) (SRA accession SRR3203222), hoopoe (*Upupa epops*) (SRA accession SRR3203224), Eurasian eagle-owl (*Bubo bubo*) (SRA accession SRR3203225), black-winged kite (*Elanus caeruleus*) (SRA accession SRR3203227), Eurasian scops owl (*Otus scops*) (SRA accession SRR3203230), common kestrel (*Falco tinnunculus*) (SRA accession SRR3203231), grey-faced buzzard (*Butastur indicus*) (SRA accession SRR3203233), besra (*Accipiter virgatus*) (SRA accession SRR3203234), cinereous vulture (*Aegypius monachus*) (SRA accession SRR3203236), Eurasian hobby (*Falco subbuteo*) (SRA accession SRR3203238), grey-headed woodpecker (*Picus canus*) (SRA accession SRR3203240), little owl (*Athene noctua*) (SRA accession SRR3203242), Indian scops owl (*Otus bakkamoena*) (SRA accession SRR3203243) (Wu et al. 2016).

2 Supplementary Results and Discussion

2.1 Scaffold numbering

2.1.1 When referring to specific scaffolds in the results and discussion sections, we have inserted a dash ("-") between the word "scaffold" and the scaffold number for legibility. These dashes are not present in any of the assembly data files. Thus, "scaffold-1085" referenced in the manuscript will appear as "scaffold1085" in the assembly and other associated files.

3 Supplementary Tables

Table S1. Sequence data collected for use in genome assembly. We here provide information on the insert size, fragmentation method, amplification, sequencing length, and raw data quantity for all libraries sequenced for this genome assembly. We have numbered the libraries and refer to these numbers in other sections of this manuscript.

Library number	Library name	Average insert size (nt)	Insert size standard deviation (nt)	Library Fragmentation method	PCR amplification used (Yes / No)	Paired-end read lengths forward / reverse (nt)	Raw reads passing onboard Illumina quality filter coverage of 1.5 Gnt genome (1X-fold coverage)
1	Nextera350nt lane 1	247	118	Nextera	Yes	100 / 100	9.80
2	Nextera350nt lane 2	247	118	Nextera	Yes	100 / 100	26.44
3	Hydroshear	500	52	Hydroshear	Yes	350 / 250	2.55
4	Nextera550nt lane 1	560	25	Nextera	Yes	300 / 300	3.65
5	Nextera550nt lane 2	560	25	Nextera	Yes	375 / 225	8.90
6	Nextera700nt	566	194	Nextera	Yes	150 / 150	31.14
7	noPCR550nt	619	132	Covaris	No	350 / 250	3.50
8	PCR900nt	687	58	Covaris	Yes	350 / 250	2.04
9	MP4kb	3,316	213	Nextera Mate Pair	Yes	100 / 100	7.84
10	MP7kb	5,904	537	Nextera Mate Pair	Yes	100 / 100	8.48
11	MP11kb	9,615	1930	Nextera Mate Pair	Yes	100 / 100	8.19

Table S2. Preliminary assembly parameters. We here report the parameters used in our preliminary assemblies using SOAPdenovo2. "Trim level" indicates the average Phred score to which we trimmed using Trimmomatic. A higher Phred score indicates a more restrictive trimming. “Error correction” refers to whether we performed error correction on the input reads for the assembly. We provide information on how we specified that the assembler use the paired-end and unpaired data for each assembly. For a given assembly, we note which libraries provided data and in which portions of the assembly process that data was used. For a given portion of the assembly process, we give the numbers of the utilized libraries followed, in parentheses, by the rank given to each library in the assembly configuration file. Please refer to Table S1 for information about the libraries to which the numbers refer. An asterisk is next to the preliminary assembly that we chose to use as the basis for the final assembly.

Assembly	Trim level	Error correction	Assembly notes	Unpaired data - only contig	Paired-end data - only scaffold	Paired-end data - both contig and scaffold	Unpaired data - only gap closure
1	28	No	N/A	1-11 (6)	9 (3), 10 (4), 11 (5)	1-3 (1), 6 (2), 7 (1), 8 (2)	None
2	28	No	N/A	1-2 (6), 4-11 (6)	9 (3), 10 (4), 11 (5)	1-2 (1), 6 (2), 7 (1), 8 (2)	None
3	28		Only reads merged with BBMerge used as unpaired data	1-5 (6), 7-8 (6)	9 (3), 10 (4), 11 (5)	1-2 (1), 6 (2), 7 (1), 8 (2)	None
4*	17	Yes	N/A	1-2 (6), 4-11 (6)	9 (3), 10 (4), 11 (5)	1-2 (1), 6 (2), 7 (1), 8 (2)	None
5	28	No	No merging of paired-end reads performed		9 (3), 10 (4), 11 (5)	1-5 (1), 6 (2), 7 (1), 8 (2)	None
6	28	No	N/A	1-11 (6)	9 (3), 10 (4), 11 (5)	1-3 (1), 6 (2), 7 (1)	None
7	28	No	N/A	1-11 (6)	9 (3), 10 (4), 11 (5)	1 (1), 2 (1), 3 (1), 4 (1), 5 (1), 6 (2), 7 (1), 8 (2)	None
8	28	No	No merging of paired-end reads performed.		9 (3), 10 (4), 11 (5)	1 (1), 2 (1), 4 (1), 5 (1), 6 (2), 7 (1), 8 (2)	None
9	28	No	Only reads merged with BBMerge used as unpaired data	1-2 (6), 4-5 (6), 7-8 (6)	9 (3), 10 (4), 11 (5)	1 (1), 2 (1), 6 (2), 7 (1), 8 (2)	None
10	28	No	Only reads merged with BBMerge used as unpaired data	1-2 (6), 4-5 (6), 7-8 (6)	9 (3), 10 (4), 11 (5)	1-2 (1), 4-5 (1), 6 (2), 7 (1), 8 (2)	None
11	17	Yes	Only reads merged with BBMerge used as unpaired data, library 3 excluded.	1-2 (6), 4-5 (6), 7-8 (6)	9 (3), 10 (4), 11 (5)	1-2 (1), 4-5 (1), 6 (2), 7 (1), 8 (2)	None
12	17	Yes	All unpaired reads used, library 3 excluded.	1-2 (6) 4-5 (6), 6-11 (6)	9 (3), 10 (4), 11 (5)	1-2 (1), 4-5 (1), 6 (2), 7 (1), 8 (2)	None
13	17	Yes	Reads merged with BBMerged used for contig assembly, other unpaired reads used only for gap closure.	1-2 (6), 4-5 (6), 7-8 (6)	9 (3), 10 (4), 11 (5)	1-2 (1), 2 (1), 4-5 (1), 6 (2), 7 (1), 8 (2)	1-2 (7), 4-11 (7)
14	7	Yes	N/A	1-2 (6), 4-11 (6)	9 (3), 10 (4), 11 (5)	1-2 (1), 4-5 (1), 6 (2), 7 (1), 8 (2)	None

Table S3. Light-associated gene searches information. This table provides details on the reference sequences used for and the results of our searches for light-associated genes in the genome assemblies of *Strix occidentalis caurina* and *Tyto alba*. "Stop" indicates the presence of a premature stop codon. "Del" indicates a frameshift deletion. "Ins" indicates a frameshift insertion.

Gene	**Reference Sequence**	***Strix occidentalis* Sequence**	***Tyto alba* Sequence**
SWS1	GenBank: AH007798 *Columba livia*	**No BLAST results**	**No BLAST results**
SWS1 notes	**Synteny:** *Taeniopygia guttata* and *Homo sapiens,* 5' end *FLNC (*REV), 3' end *CALU* (REV); *Anolis carolinensis*, 3' end *CALU* (REV)	*FLNC:* scaffold-4221<br>*CALU:* scaffold-15	No gene predictions for *FLNC* or *CALU* in *Tyto*
SWS2	GenBank: AH007799 *Columba livia*	scaffold-4153 & scaffold-7110: Functional	**No BLAST results**
SWS2 notes	**Synteny:** *Anolis carolinensis* and *Xenopus laevis* 5' end *MECP2* (REV), 3' end *LWS;* avian contigs in Ensembl are very short and do not include flanking genes	Only exons 1 (partial), 2 and 3 recovered; partial exon 1 flanked by N's, and exon 3 is towards the end of the scaffold; 2 different scaffolds; 100% identical except 1-nt diff in exon 3, nonsynonymous	*MECP2* and *LWS* not predicted in *Tyto*
Rh1	GenBank: AH007730 *Columba livia*	scaffold-133: Functional	JJRD01003728, JJRD01003729: Functional
Rh2	GenBank: AH007731 *Columba livia*	scaffold-1932: Functional	**JJRD01131248, JJRD01131249: Pseudogene (exon 1: 29-nt del; exon 2: stop; exon 3: stop; exon 4: 2-nt del)**
LWS	GenBank: AH007800 *Columba livia*	scaffold-6263: Functional	**No BLAST results**
LWS notes	**Synteny:** *Anolis carolinensis* 5' end *SWS2,* 3' end TEX28 (REV); *Xenopus laevis* 5' end *SWS2*, 3' end *AVPR2*; avian contigs in Ensembl are very short and do not include flanking genes	Only exons 2, partial 5 and 6; 3-5 are N's, no hits for exon 1	No gene predictions for *SWS2, AVPR2* or *TEX28*
OpnP	GenBank: U15762, WGS: AADN03007691 *Gallus gallus*	**No BLAST results; After BLASTing intergenic region, has hit with *Gallus gallus* genomic pinopsin, non-coding region 5' of cds is retained**	**JJRD01162372, JJRD01162373: Pseudogene (exon 1: start codon mutation ACA, 13-nt del, 2-nt ins, 1-nt del, exon 2: 1-nt del; intron 3-exon 4 boundary: 21 nt-del; exon 4: 7-nt del, 2-nt del; exon 5: 1-nt del)**
OpnP notes	**Synteny:** *Gallus gallus DOC2B,* 5' end, 3' end *TEX14* (REV); *Ficedula albicollis DOC2B*, 5' end	DOC2B: scaffold-86<br>TEX14: scaffold-86	
OpnVA	GenBank: EF055883, WGS: AADN03005037 *Gallus gallus*	Scaffold205: Functional	JJRD01088850, JJRD01088852, JJRD01106859, JJRD01168068: Functional

Gene	**Reference Sequence**	***Strix occidentalis* Sequence**	***Tyto alba* Sequence**
Opn4x	GenBank: NM_204625, WGS: AADN03004364 *Gallus gallus*	scaffold-147: Functional	JJRD01038044: Functional
Opn4m	GenBank: AY882944, WGS: AADN04000143 *Gallus gallus*	**scaffold-219: Pseudogene? (exon 8: stop, 4-nt del)**	**JJRD01098086, JJRD01098087: Pseudogene? (exon 8: 4-nt del; intron 11: splice donor mutation GT to AT)**
Opn3	GenBank: XM_426139, WGS: AADN04000318 *Gallus gallus*	scaffold-728: Functional	JJRD01072701: Functional (No BLAST results for exon 1)
Opn5	GenBank: NM_001130743 WGS: AADN04000287 *Gallus gallus*	scaffold-546: Functional	JJRD01001581, JJRD01133804: Functional
Opn5L1	GenBank: NM_001310056, WGS: AADN04000228 *Gallus gallus*	scaffold-6: Functional	JJRD01004196: Functional
Opn5L2	GenBank: NM_001162892, WGS: AADN04000287 *Gallus gallus*	scaffold-722: Functional	JJRD01082691: Functional
RRH	GenBank: NM_001079759, WGS: AADN04000018 *Gallus gallus*	scaffold-22: Functional	JJRD01123735: Functional
RGR	GenBank: NM_001031216, WGS: AADN04000143 *Gallus gallus*	scaffold-219: Functional	JJRD01065549: Functional
EEVS-like	GenBank: XM_013180282, WGS: AOGC01018216 *Anser cygnoides*	scaffold-133: Functional	JJRD01160345: Functional
MT-Ox	GenBank: XM_015293238, WGS: AADN04000009 *Gallus gallus*	scaffold-133: Functional	JJRD01160345, JJRD01160346, JJRD01160347, JJRD01160348: Functional
Photolyase	GenBank: XM_422729, WGS: AADN04000078 *Gallus gallus*	scaffold-742: Functional	JJRD01136093, JJRD01136094: Functional
CYP2J19	GenBank: XM_422553, WGS: AADN04000032 *Gallus gallus*	**scaffold-313: Pseudogene? (exon 9: 1-nt ins, 2-nt del)**	**JJRD01034859: Pseudogene (exon 1: stop; exon 3: 5-nt del; exon 5: stop; exon 6: stop)**

Table S4. Assembly metrics with a range of cutoffs. These are statistics on the final (post gap closing) assembly that display the consequence of choosing various cutoffs for minimum scaffold length and the number of N's that separate a contig. We have marked the line with the cutoffs and statistics that correspond to the final chosen assembly version with an asterisk.

Scaffold minimum length (nt)	Scaffold N50 (nt)	Scaffold L50	Number of Scaffolds	Total sequence length (nt)	Number of N's to split contigs	Contig N50	Contig L50	Number of contigs	Total sequence length (nt)
1000*	3,983,020	92	8,113	1,255,568,683	25	171,882	2,057	27,258	1,241,846,690
1000	-	-	-	-	20	167,327	2,112	27,729	1,241,836,309
1000	-	-	-	-	15	163,476	2,166	28,200	1,241,828,287
1000	-	-	-	-	10	159,062	2,233	28,719	1,241,822,133
1000	-	-	-	-	5	155,200	2,286	29,229	1,241,818,593
1000	-	-	-	-	1	51,301	7,054	65,092	1,241,782,051
500	3,937,821	93	17,952	1,262,291,236	25	170,589	2,076	37,544	1,248,502,317
500	-	-	-	-	20	166,062	2,132	38,023	1,248,491,764
500	-	-	-	-	15	162,595	2,186	38,504	1,248,483,572
500	-	-	-	-	10	158,193	2,254	39,038	1,248,477,239
500	-	-	-	-	5	153,747	2,308	39,562	1,248,473,599
500	-	-	-	-	1	50,930	7,119	76,379	1,248,436,081
300	3,915,799	95	48356	1,273,290,518	25	168,721	2,109	67,949	1,259,501,544
300	-	-	-	-	20	164,817	2,166	68,428	1,259,490,991
300	-	-	-	-	15	161,269	2,220	68,909	1,259,482,799
300	-	-	-	-	10	156,434	2,289	69,443	1,259,476,466
300	-	-	-	-	5	152,072	2,344	69,967	1,259,472,826
300	-	-	-	-	1	50,425	7,228	106,823	1,259,435,266
None	1,836,279	209	3,754,965	1,882,109,172	25	81,400	4,678	3,774,558	1,868,320,198
None	-	-	-	-	20	79,089	4,800	3,775,037	1,868,309,645
None	-	-	-	-	15	77,624	4,921	3,775,518	1,868,301,453
None	-	-	-	-	10	76,045	5,061	3,776,052	1,868,295,120
None	-	-	-	-	5	73,935	5,180	3,776,576	1,868,291,480
None	-	-	-	-	1	25,761	15,609	3,813,432	1,868,253,920

Table S5. Final SOAPdenovo2 parameters. This table lists the SOAPdenovo2 parameters that we specified for each library to generate the final assembly.

Library	Paired or unpaired reads	Configuration file insert size (nt)	Used in contig or scaffold building	Assembly usage rank	Pair number cutoff	Mapping length (nt)
Nextera350nt lane 1	paired	247	both	1	3	32
Nextera350nt lane 2	paired	247	both	1	3	32
Nextera700nt	paired	566	both	2	3	32
noPCR550nt	paired	619	both	1	3	32
PCR900nt	paired	687	both	2	3	32
MP4kb	paired	3,316	scaffold	3	5	35
MP7kb	paired	5,904	scaffold	4	5	35
MP11kb	paired	9,615	scaffold	5	5	35
Nextera350nt lane 1	unpaired	N/A	contig	6	3	32
Nextera350nt lane 2	unpaired	N/A	contig	6	3	32
Nextera550nt lane 1	unpaired	N/A	contig	6	3	32
Nextera550nt lane 2	unpaired	N/A	contig	6	3	32
Nextera700nt	unpaired	N/A	contig	6	3	32
noPCR550nt	unpaired	N/A	contig	6	3	32
PCR900nt	unpaired	N/A	contig	6	3	32
MP4kb	unpaired	N/A	contig	6	3	32
MP7kb	unpaired	N/A	contig	6	3	32
MP11kb	unpaired	N/A	contig	6	3	32

Table S6. Full assembly metrics. Listed here are metrics on the full assembly (no contaminate or mitochondrial sequences removed) before gap-closing, after gap-closing, and after gap-closing and removal of all contigs and scaffolds less than 1000 nt in length. Strings of 25 or more N's broke scaffolds into contigs.

Assembly version	No gap-closing, scaffolds and contigs <1000 nt removed	Gap-closed, no scaffolds or contigs removed	Gap-closed, scaffolds and contigs <1000 nt removed
Number of scaffolds	3,754,965	3,754,965	8,113
Total size of scaffolds	1,884,424,465 nt	1,882,109,172 nt	1,255,568,683 nt
Longest scaffold	15,783,852 nt	15,750,186 nt	15,750,186 nt
Shortest scaffold	128 nt	128 nt	1,000 nt
Number of scaffolds > 1K nt	8,117 (0.2%)	8,100 (0.2%)	8,100 (99.8%)
Number of scaffolds > 10K nt	1,755 (0.0%)	1,747 (0.0%)	1,747 (21.5%)
Number of scaffolds > 100K nt	661 (0.0%)	661 (0.0%)	661 (8.1%)
Number of scaffolds > 1M nt	303 (0.0%)	303 (0.0%)	303 (3.7%)
Number of scaffolds > 10M nt	9 (0.0%)	9 (0.0%)	9 (0.1%)
Mean scaffold size	502 nt	501 nt	154,760 nt
Median scaffold size	150 nt	150 nt	1,903 nt
N50 scaffold length (L50 scaffold count)	1,843,286 nt (209)	1,836,279 nt (209)	3,983,020 nt (92)
N60 scaffold length (L60 scaffold count)	622,124 nt (370)	619,581 nt (371)	3,012,707 nt (129)
N70 scaffold length (L70 scaffold count)	255 nt (216,224)	255 nt (218,948)	2,142,451 nt (178)
N80 scaffold length (L80 scaffold count)	174 nt (1,110,557)	174 nt (1,113,218)	1,545,070 nt (246)
N90 scaffold length (L90 scaffold count)	143 nt (2,336,944)	143 nt (2,338,563)	618,731 nt (372)
scaffold %GC	42.81%	43.82%	41.31%
scaffold %N	2.89%	0.74%	1.10%
Percentage of assembly in scaffolded contigs	66.4%	65.7%	98.5%
Percentage of assembly in unscaffolded contigs	33.6%	34.3%	1.5%
Average number of contigs per scaffold	1.0	1.0	3.4
Average length of break (>25 Ns) between contigs in scaffold	311	703	716
Number of contigs	3,929,051	3,774,558	27,258
Number of contigs in scaffolds	179,957	22,374	21,480
Number of contigs not in scaffolds	3,749,094	3,752,184	5,778
Total size of contigs	1,830,129,061 nt	1,868,320,198 nt	1,241,846,690 nt
Longest contig	186,255 nt	1,259,046 nt	1,259,046 nt
Shortest contig	5 nt	128 nt	130 nt
Number of contigs > 1K nt	123,899 (3.2%)	23,921 (0.6%)	23,921 (87.8%)
Number of contigs > 10K nt	37,347 (1.0%)	12,374 (0.3%)	12,374 (45.4%)
Number of contigs > 100K nt	58 (0.0%)	3,909 (0.1%)	3,909 (14.3%)
Number of contigs > 1M nt	0 (0.0%)	8 (0.0%)	8 (0.0%)
Mean contig size	466 nt	495 nt	45,559 nt
Median contig size	150 nt	150 nt	6,696 nt
N50 contig length (L50 contig count)	7,855 nt (46,857)	81,400 nt (4,678)	171,882 nt (2,057)
N60 contig length (L60 contig count)	3,275 nt (81,604)	33521 nt (8,121)	134,419 nt (2,876)
N70 contig length (L70 contig count)	254 nt (448,713)	255 nt (254,707)	98,599 nt (3,956)
N80 contig length (L80 contig count)	170 nt (1,346,253)	173 nt (1,148,670)	66,629 nt (5,485)
N90 contig length (L90 contig count)	142 nt (2,548,885)	142 nt (2,367,834)	34,559 nt (8,023)

Table S7. Statistics from after quality-filtering MAKER annotations. This is a table of annotation summary statistics resulting from quality-filtering our MAKER pipeline annotation output.

	Values post -s filter
parsed genome node DAGs	745,622
sequence regions	8,112 (total length: 1,255,013,157 nt)
multi-features	15,712
genes	16,718
protein-coding genes	16,718
mRNAs	16,718
protein-coding mRNAs	16,718
exons	146,689
CDSs	146,217

Table S8. Mitochondrial genome assembly gene annotations. This is a table of the gene annotations of the assembly of a partial mitochondrial genome represented by scaffold-3674. The coordinates are 1-based.

Gene	Scaffold	Start position	End position	Direction
tRNA^{Thr}	scaffold3674	231	299	-
Cytb	scaffold3674	307	1431	-
ND5	scaffold3674	1463	3268	-
tRNA^{Leu1}	scaffold3674	3269	3339	-
tRNA^{Ser1}	scaffold3674	3342	3407	-
tRNA^{His}	scaffold3674	3410	3479	-
ND4L	scaffold3674	3490	4857	-
ND4L	scaffold3674	4854	5147	-
tRNA^{Arg}	scaffold3674	5149	5218	-
ND3_b	scaffold3674	5224	5397	-
ND3_a	scaffold3674	5399	5572	-
tRNA^{Gly}	scaffold3674	5573	5641	-
COIII	scaffold3674	5643	6425	-
ATP6	scaffold3674	6431	7108	-
ATP8	scaffold3674	7105	7266	-
tRNA^{Lys}	scaffold3674	7268	7338	-
COII	scaffold3674	7357	8031	-
tRNA^{Asp}	scaffold3674	8034	8102	-
tRNA^{Ser2}	scaffold3674	8106	8177	+
COI	scaffold3674	8178	9710	-
tRNA^{Tyr}	scaffold3674	9721	9791	+
tRNA^{Cys}	scaffold3674	9792	9860	+
tRNA^{Asn}	scaffold3674	9863	9936	+
tRNA^{Ala}	scaffold3674	9938	10006	+
tRNA^{Trp}	scaffold3674	10008	10083	-
ND2	scaffold3674	10094	11122	-
tRNA^{Met}	scaffold3674	11123	11191	-
tRNA^{Gln}	scaffold3674	11191	11261	+
tRNA^{Ile}	scaffold3674	11273	11344	-
ND1	scaffold3674	11352	12299	-
tRNA^{Leu2}	scaffold3674	12314	12387	-
16S	scaffold3674	12387	13982	-
tRNA^{Val}	scaffold3674	13983	14054	-
12S	scaffold3674	14054	15041	-
tRNA^{Phe}	scaffold3674	15041	15108	-
tRNA^{Glu}	scaffold3674	21542	21614	+

Table S9. Information on searches for light-associated genes in non-owl genome assemblies. This table provides information on the results of our searches for a subset of the light-associated genes in several non-owl avian genome assemblies. "Stop" indicates the presence of a premature stop codon. "Del" indicates a frameshift deletion. For these searches we employed the same reference sequences used in the owl genome searches, detailed in Table S3.

	Rh2	***OpnP***	***Opn4m***	***CYP2J19***
**Reference Sequence**	GenBank: AH007731 *Columba livia*	GenBank: U15762, WGS: AADN03007691 *Gallus gallus*	GenBank: AY882944, WGS: AADN04000143 *Gallus gallus*	GenBank: XM_422553, WGS: AADN04000032 *Gallus gallus*
***Aquila chrysaetos* Sequence**	JRUM01011001	JRUM01006324	**JRUM01004396: Pseudogene? (exon 9: stop)**	JRUM01002169
***Cathartes aura* Sequence**	JMFT01083953	JMFT01020150, JMFT01020151, JMFT01020152, JMFT01020153	JMFT01012857, JMFT01012858, JMFT01012859	JMFT01168756
***Colius striatus* Sequence**	JJRP01038063, JJRP01092220	JJRP01068983	**JJRP01099016, JJRP01099018, JJRP01099019: Pseudogene? (exon 9: 1-bp del; intron 9: splice donor mutation GT to TT; exon 11: stop)**	JJRP01092926
***Leptosomus discolor* Sequence**	JJRK01095962, JJRK01095963	JJRK01016598, JJRK01016599	**JJRK01001211, JJRK01001212, JJRK01001213: Pseudogene? (intron 10: splice donor mutation GT to GA)**	JJRK01096026
***Apaloderma vittatum* Sequence**	JMFV01047445, JMFV01047446	JMFV01046166, JMFV01046167	JMFV01094831	JMFV01067118, JMFV01102670, JMFV01104326, JMFV01105382
***Buceros rhinoceros* Sequence**	JMFK01024225	JMFK01144445, JMFK01144446, JMFK01144447, JMFK01144448,	**JMFK01158949, JMFK01158950, JMFK01158951, JMFK01158952: Pseudogene? (exon 1: start codon mutation CTG)**	JMFK01006414, JMFK01073748
***Picoides pubescens* Sequence**	JJRU01080411, JJRU01080413	JJRU01064065	JJRU01054812	JJRU01010544, JJRU01010545
***Merops nubicus* Sequence**	JJRJ01051189	JJRJ01058175	JJRJ01007844	JJRJ01011917, JJRJ01033855

Table S10. Details of branch tests. This table gives the details of the branch tests performed to test for evidence of changes in selection pressure on the owl branches. "BG" indicates the background branches, "lnL" denotes the log likelihood of the model, "LRT" denotes the value of the likelihood ratio test (given by 2 times the difference in the likelihoods of the models), and "cf" denotes the codon frequency model used to calculate the equilibrium codon frequencies with "cf 1" indicating that we used the average nucleotide frequencies and "cf 2" indicating that we used the average nucleotide frequencies at each of the 3 codon positions. "Model" corresponds to the number of ω values employed among branches with one ω value assumed for all branches under model "0", two ω values used under model "1", and 3 ω values used with model "2". "*Tyto*" and "*Strix*" indicate whether the value pertains to sequence in the *Tyto alba* or *Strix occidentalis caurina* genome assembly, respectively. For model comparisons, bold font indicates significant difference ($p < 0.05$) between models.

Gene	Model	BG ω	*Tyto* ω	*Strix* ω	Stem Owl ω	lnL	Models compared	LRT
***CYP2J19* (cf 1)**	0	0.206				-5045.714		
	1	0.173	0.719			-5029.495	**1 vs. 0**	32.437
	2	0.164	0.719	0.336		-5027.178	**2 vs. 1**	4.633
***CYP2J19* (cf 2)**	0	0.194				-5050.277		
	1	0.163	0.681			-5034.027	**1 vs. 0**	32.499
	2	0.154	0.680	0.333		-5031.418	**2 vs. 1**	5.219
***OPN4M* (cf 1)**	0	0.214				-3345.378		
	1	0.192	0.448	0.448	0.895	-3341.951	1 vs. 0	6.854
***OPN4M* (cf 2)**	0	0.213				-3350.019		
	1	0.190	0.452	0.452	0.864	-3346.487	**1 vs. 0**	7.066
***OPNP* (cf 1)**	0	0.234				-3937.560		
	1	0.180	0.695			-3918.377	**1 vs. 0**	38.446
***OPNP* (cf 2)**	0	0.152				-3892.939		
	1	0.114	0.508			-3870.379	**1 vs. 0**	45.121
***RH2* (cf 1)**	0	0.079				-3155.354		
	1	0.057	0.367			-3139.086	**1 vs. 0**	32.536
	2	0.052	0.358	0.208		-3136.501	**2 vs. 1**	5.170
***RH2* (cf 2)**	0	0.043				-3054.733		
	1	0.031	0.219			-3037.200	**1 vs. 0**	35.065
	2	0.028	0.205	0.158		-3033.836	**2 vs. 1**	6.728

Table S11. Details of branch-site tests. This table provides details of the tests performed using branch-site models implemented in the phylogenetic analysis by maximum likelihood (PAML) package to detect positive selection affecting certain sites on the owl lineages. "*Tyto*" and "*Strix*" indicate whether the values pertain to sequence in the *Tyto alba* or *Strix occidentalis caurina* genome assembly, respectively. "BG" indicates the background branches, "FG" denotes the foreground branch, "lnL" denotes the log likelihood of the model, "LRT" denotes the value of the likelihood ratio test (given by 2 times the difference in the likelihoods of the models), and "cf" denotes the codon frequency model used to calculate the equilibrium codon frequencies with "cf 1" indicating that we used the average nucleotide frequencies and "cf 2" indicating that we used the average nucleotide frequencies at each of the 3 codon positions. "Site class" indicates the ω category with "0" indicating sites under purifying selection, "1" sites under relaxed selection, "2a" sites that are under positive selection on the foreground branch and under purifying selection on the background branches, and "2b" indicating positive selection on the foreground branch and relaxed selection on the background branches. "Proportion" indicates the proportion of sites in a given class. "Model" denotes either the positive selection model ("Positive") or the null model ("Null").

Gene	Taxon	Site class	Proportion	BG ω	FG ω	Model	lnL	LRT
***OPN4M* (cf 1)**	*Strix*	0	0.778	0.047	0.047			
		1	0.184	1	1			
		2a	0.031	0.047	4.291			
		2b	0.007	1	4.291			
						Positive	-3305.681	
						Null	-3305.984	-0.605
	Tyto	0	0.773	0.046	0.046			
		1	0.190	1	1			
		2a	0.030	0.046	1.660			
		2b	0.007	1	1.660			
						Positive	-3306.308	
						Null	-3306.325	-0.033
***OPN4M* (cf 2)**	*Strix*	0	0.773	0.047	0.047			
		1	0.182	1	1			
		2a	0.036	0.047	4.051			
		2b	0.009	1	4.051			
						Positive	-3310.564	
						Null	-3310.887	-0.646
	Tyto	0	0.788	0.050	0.050			
		1	0.189	1	1			
		2a	0.019	0.050	2.072			
		2b	0.004	1	2.072			
						Positive	-3311.582	
						Null	-3311.605	-0.047

4 Supplementary Figures

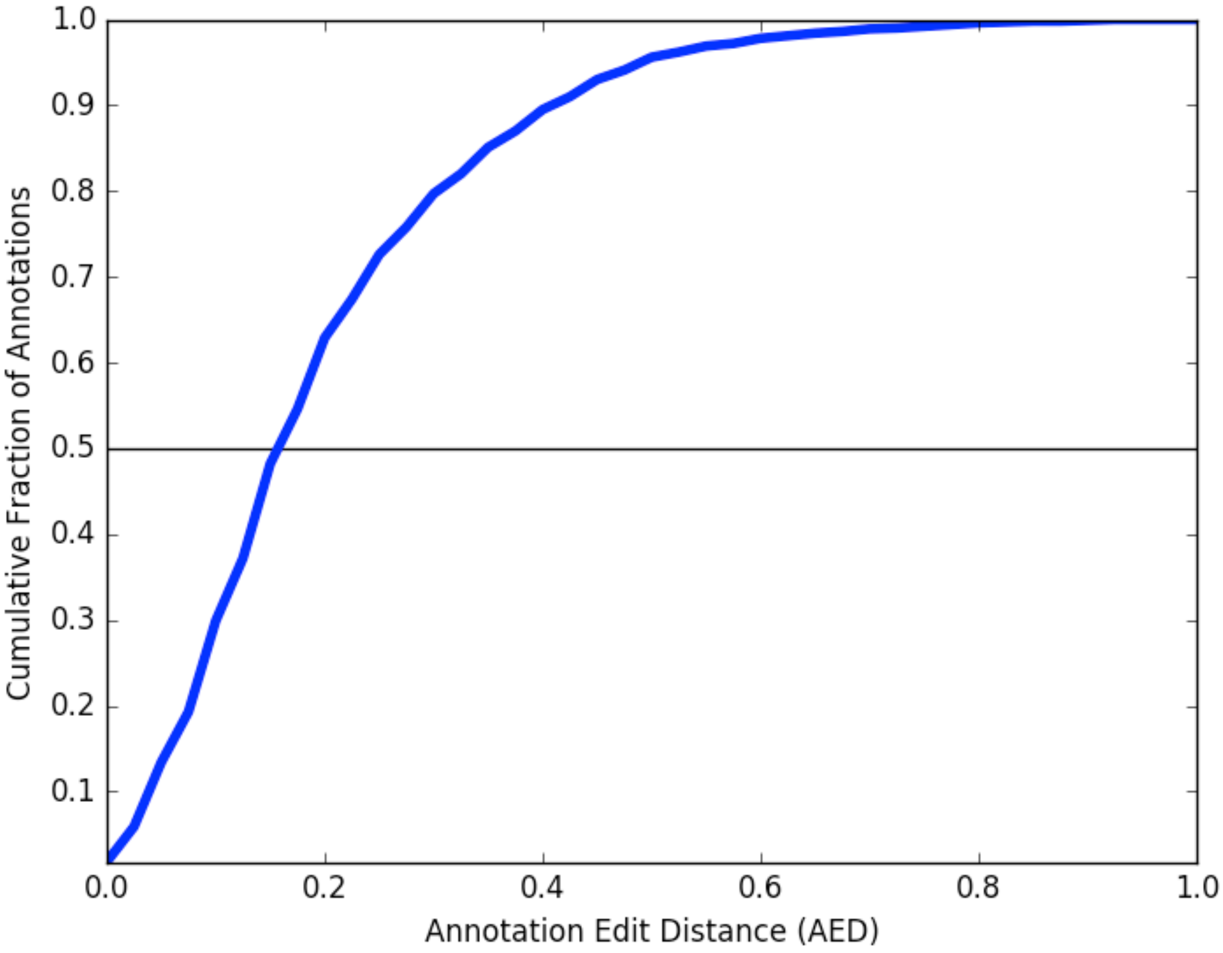

Figure S1. Cumulative distribution of annotation edit distances of MAKER-generated annotations. This is a graph of the cumulative distribution of annotation edit distances (AED) of the annotations generated by MAKER. Included here are all of the annotations in the MAKER final output. We have drawn a horizontal line denoting 50% of the annotations. After quality filtering, the cumulative distribution appeared identical.

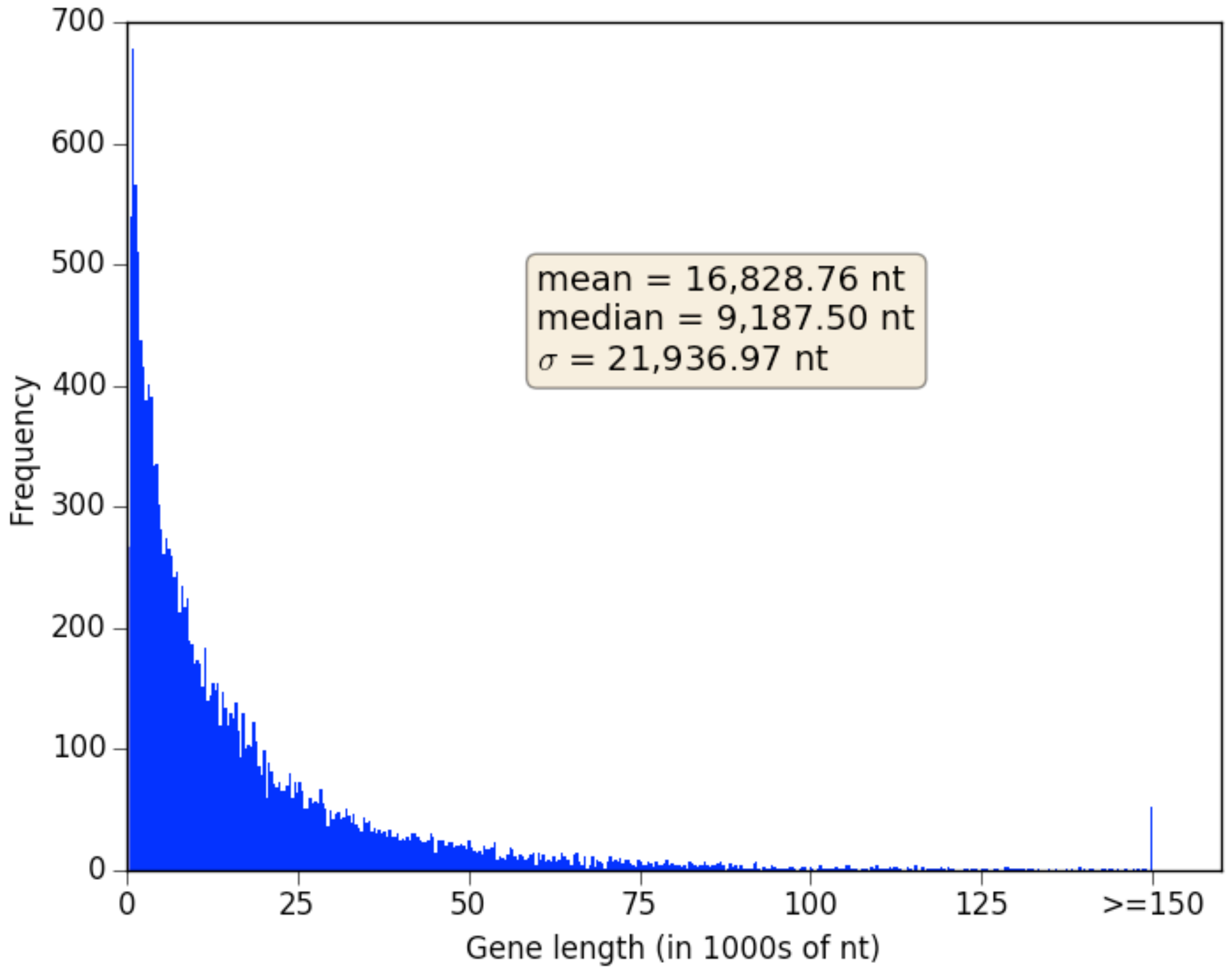

Figure S2. Histogram of the lengths of genes annotated by MAKER. This is a histogram of the distribution of the lengths of genes annotated by MAKER. We included all of the gene annotations in the MAKER final output. We grouped the values into 400 frequency bins, one of these including all genes greater than or equal to 150,000 nt in length. We have provided the mean, median, and standard deviation of the gene lengths in a text box.

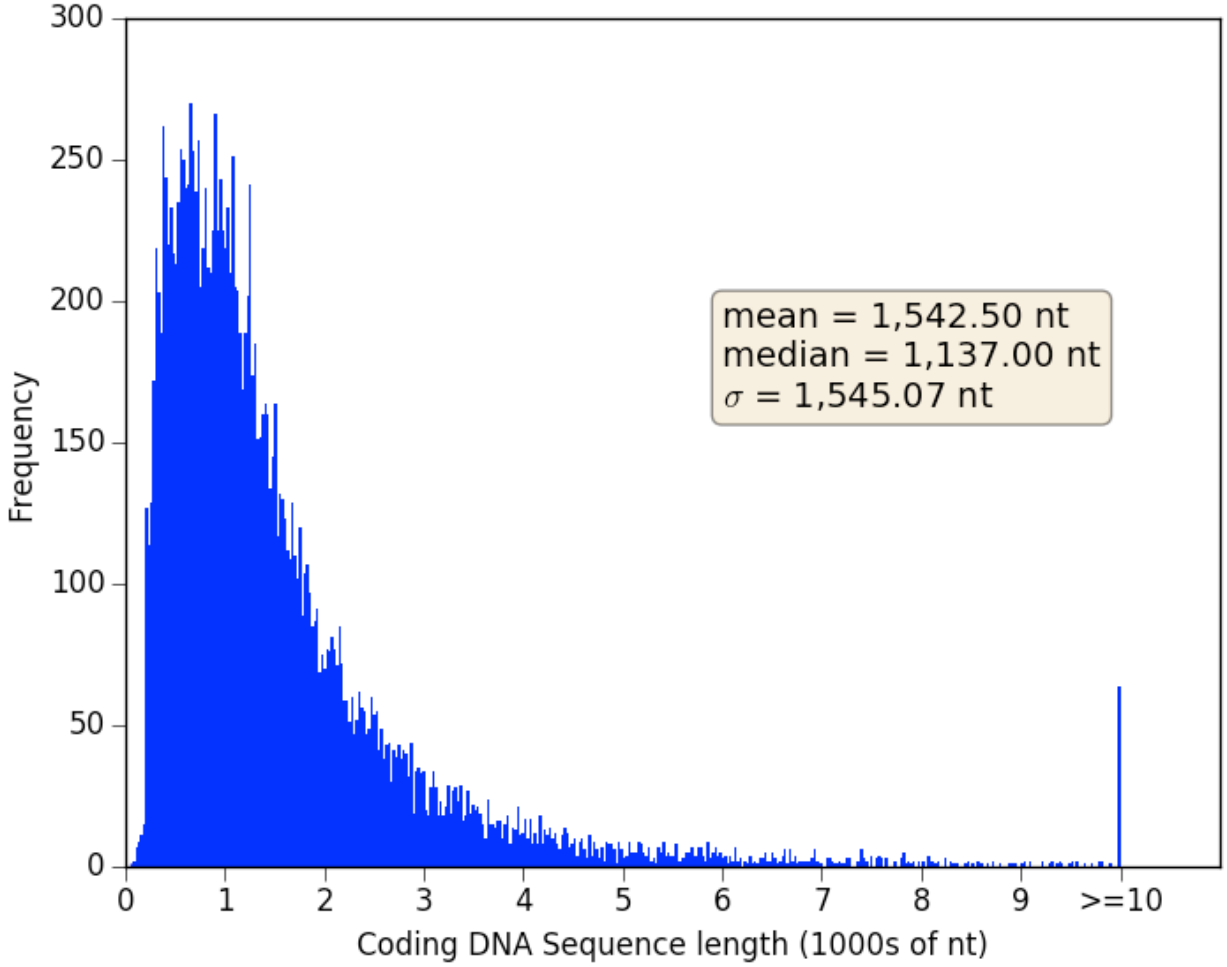

Figure S3. Histogram of the coding DNA sequence length in genes annotated by MAKER. This is a histogram of the lengths of coding DNA sequences in genes annotated by MAKER. We included all of the gene annotations in the MAKER final output. We grouped the values into 400 frequency bins, one of these including all coding DNA sequences greater than or equal to 10,000 nt in length. We have provided the mean, median, and standard deviation of the lengths in a text box.

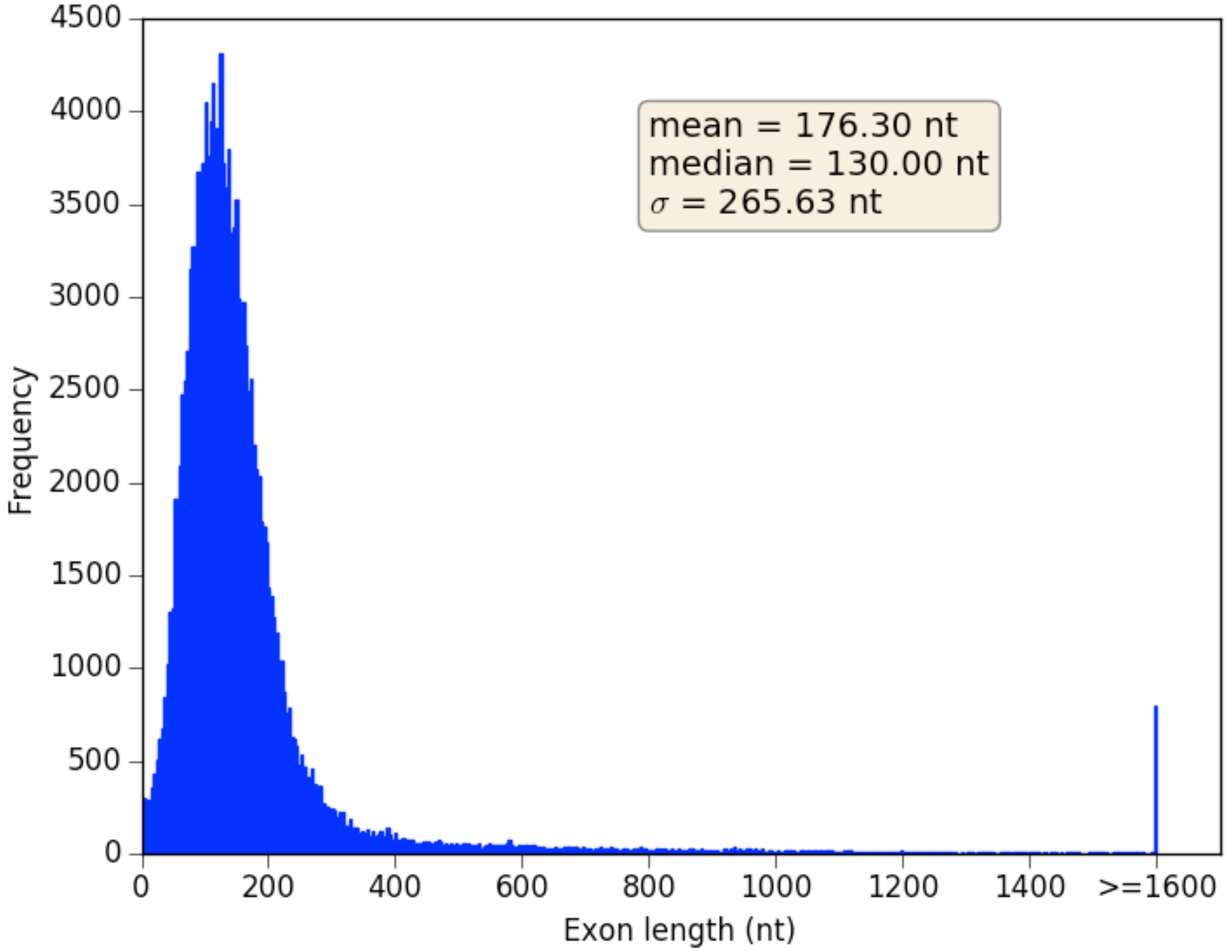

Figure S4. Histogram of the lengths of exons in genes annotated by MAKER. This is a histogram of the lengths of exons in genes annotated by MAKER. We included the exons from all of the gene annotations in the MAKER final output. We grouped the values into 400 frequency bins, one of these including all exons greater than or equal to 1,600 nt in length. We have provided the mean, median, and standard deviation of the exon lengths in a text box.

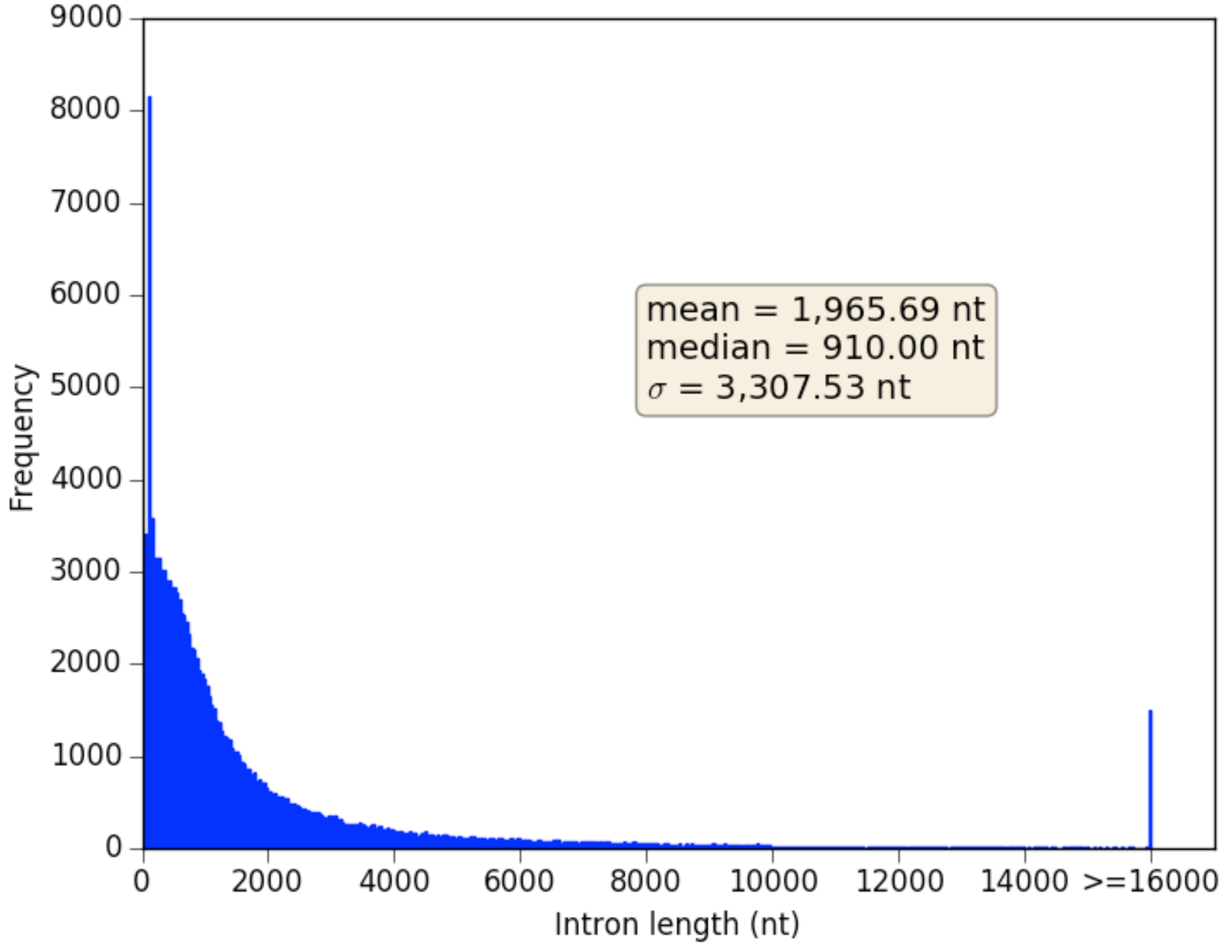

Figure S5. Histogram of the lengths of introns in genes annotated by MAKER. This is a histogram of the lengths of introns in genes annotated by MAKER. We included the introns from all of the gene annotations in the MAKER final output. We grouped the values into 400 frequency bins, one of these including all introns greater than or equal to 16,000 nt in length. We have provided the mean, median, and standard deviation of the intron lengths in a text box.

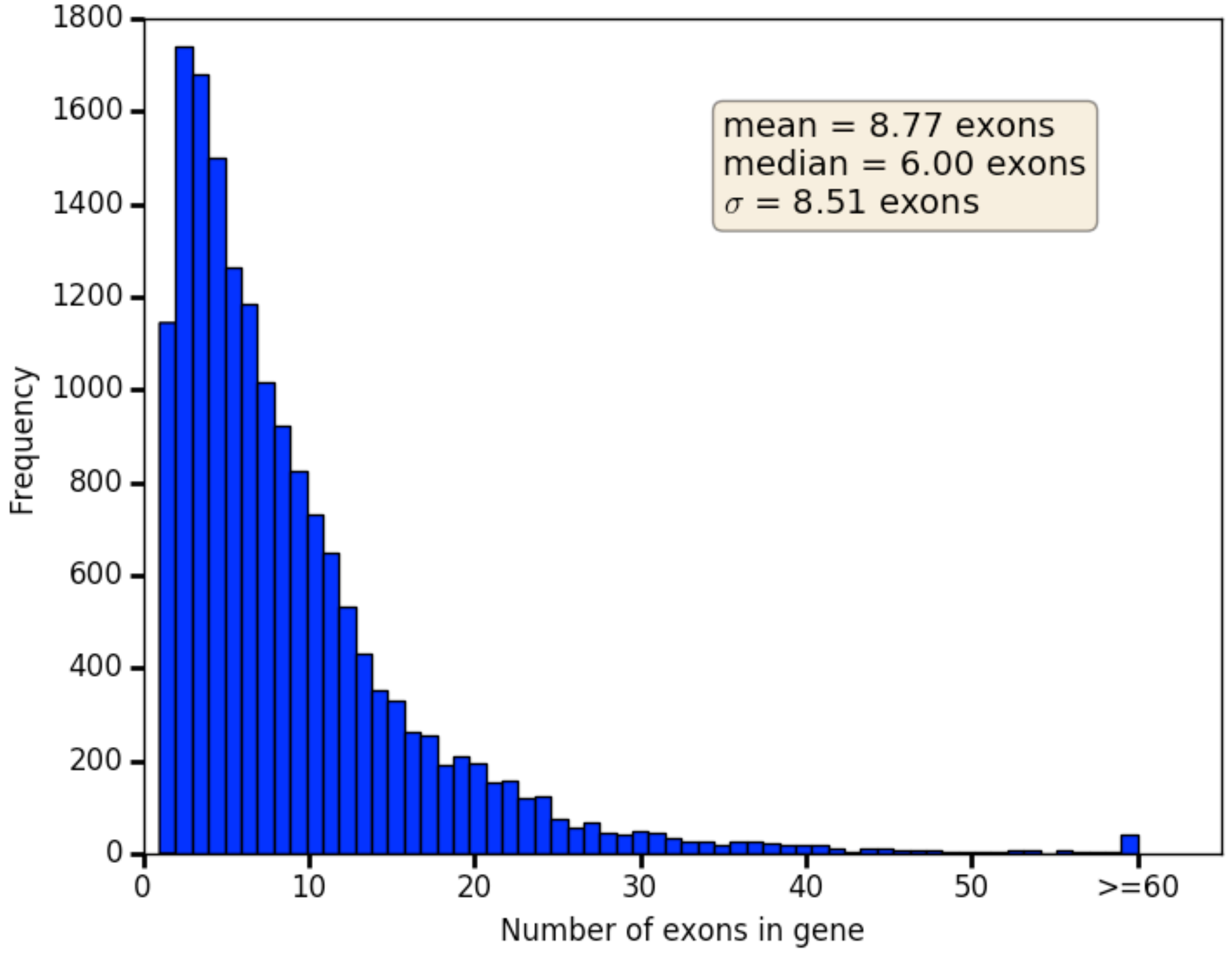

Figure S6. Histogram of the number of exons in genes annotated by MAKER. This is a histogram of the number of exons in genes annotated by MAKER. We included the exons from all of the gene annotations in the MAKER final output. We grouped the values into 60 frequency bins, one of these including all genes with greater than or equal to 60 exons. We have provided the mean, median, and standard deviation of the number of exons per gene in a text box.

5 Supplementary References

Altschul SF et al. 1997. Gapped BLAST and PSI-BLAST: a new generation of protein database search programs. Nucl. Acids Res. 25:3389–3402. doi: 10.1093/nar/25.17.3389.

Bao W, Kojima KK, Kohany O. 2015. Repbase Update, a database of repetitive elements in eukaryotic genomes. Mob DNA. 6(1):1-6. doi: 10.1186/s13100-015-0041-9.

Bao Z, Eddy SR. 2002. Automated De Novo Identification of Repeat Sequence Families in Sequenced Genomes. Genome Res. 12:1269–1276. doi: 10.1101/gr.88502.

Barnett D, Garrison E, Marth G, Strömberg M. 2015. BamTools. Version 2.4.0. [Accessed 2016 Oct 1]. Available from: https://github.com/pezmaster31/bamtools

Barnett DW, Garrison EK, Quinlan AR, Strömberg MP, Marth GT. 2011. BamTools: a C++ API and toolkit for analyzing and managing BAM files. Bioinformatics. 27:1691–1692. doi: 10.1093/bioinformatics/btr174.

Benson G. 1999. Tandem repeats finder: a program to analyze DNA sequences. Nucl. Acids Res. 27:573–580. doi: 10.1093/nar/27.2.573.

Benson G. 2012. Tandem Repeats Finder. Version 4.07b. [Accessed 2016 Oct 1]. Available from: https://tandem.bu.edu/trf/trf.html

Bernt M et al. 2013. MITOS: Improved de novo metazoan mitochondrial genome annotation. Molecular Phylogenetics and Evolution. 69:313–319. doi: 10.1016/j.ympev.2012.08.023.

Biomatters. 2016a. Geneious. Version 9.1.4. [Accessed 2016 Oct 1]. Available from: http://www.geneious.com

Biomatters. 2016b. Geneious. Version 9.1.6. [Accessed 2016 Oct 1]. Available from: http://www.geneious.com

Birney E. Wise2. Version 2.2.3-rc7. [Accessed 2016 Oct 1]. Available from: http://korflab.ucdavis.edu/datasets/cegma/#SCT4

Birney E, Clamp M, Durbin R. 2004. GeneWise and Genomewise. Genome Res. 14:988–995. doi: 10.1101/gr.1865504.

Blanco E et al. 2011. geneid. Version 1.1.4. [Accessed 2016 Oct 1]. Available from: http://genome.crg.es/software/geneid

Bolger AM, Lohse M, Usadel B. 2014. Trimmomatic: a flexible trimmer for Illumina sequence data. Bioinformatics. 30:2114–2120. doi: 10.1093/bioinformatics/btu170.

Bradnam KR et al. 2013. Assemblathon 2: evaluating de novo methods of genome assembly in three vertebrate species. Gigascience. 2:10. doi: 10.1186/2047-217X-2-10.

Bushnell B. 2014. BBMap. Version 34.00. [Accessed 2016 Oct 1]. Available from: http://sourceforge.net/projects/bbmap

Camacho C et al. 2009. BLAST+: architecture and applications. BMC Bioinformatics. 10:421. doi: 10.1186/1471-2105-10-421.

Campbell MS, Holt C, Moore B, Yandell M. 2014. Genome Annotation and Curation Using MAKER and MAKER-P. Curr Protoc Bioinformatics. 48:4.11.1-4.11.39. doi: 10.1002/0471250953.bi0411s48.

Cantarel BL et al. 2008. MAKER: An easy-to-use annotation pipeline designed for emerging model organism genomes. Genome Res. 18(1):188–196. doi: 10.1101/gr.6743907.

Clark K, Karsch-Mizrachi I, Lipman DJ, Ostell J, Sayers EW. 2016. GenBank. Nucleic Acids Res. 44:D67–D72. doi: 10.1093/nar/gkv1276.

CNHM<USA-OH>:ORNITH:B41533. Ornithology collection, Museum of Natural History & Science, Cincinnati Museum Center, Cincinnati, Ohio, United States of America.

Consortium TU. 2015. UniProt: a hub for protein information. Nucl. Acids Res. 43:D204–D212. doi: 10.1093/nar/gku989.

De Vita R, Cavallo D, Eleuteri P, Dell'Omo G. 1994. Evaluation of interspecific DNA content variations and sex identification in Falconiformes and Strigiformes by flow cytometric analysis. Cytometry. 16:346–350. doi: 10.1002/cyto.990160409.

DePristo MA et al. 2011. A framework for variation discovery and genotyping using next-generation DNA sequencing data. Nat Genet. 43:491–498. doi: 10.1038/ng.806.

Doležel J, Bartoš J, Voglmayr H, Greilhuber J. 2003. Letter to the editor. Cytometry. 51A:127–128. doi: 10.1002/cyto.a.10013.

Free Software Foundation. 2012. GNU Awk . Version 4.0.1. [Accessed 2016 Oct 1]. Available from: https://www.gnu.org/software/gawk/

Fridolfsson A-K, Ellegren H. 1999. A Simple and Universal Method for Molecular Sexing of Non-Ratite Birds. Journal of Avian Biology. 30:116–121. doi: 10.2307/3677252.

Gabriel E et al. 2004. Open MPI: Goals, Concept, and Design of a Next Generation MPI Implementation. In: Proceedings, 11th European PVM/MPI Users' Group Meeting. Budapest, Hungary pp. 97–104.

Gailly J, Adler M. 2013. zlib. Version 1.2.8. [Accessed 2016 Oct 1]. Available from: http://www.zlib.net

google-sparsehash@googlegroups.com. 2012. Google SparseHash. Version 2.0.2. [Accessed 2016 Oct 1]. Available from: https://github.com/sparsehash/sparsehash

Granlund T, Stallman RM. 2013. cat (GNU coreutils). Version 8.21. [Accessed 2016 Oct 1]. Available from: http://www.gnu.org/software/coreutils/coreutils.html

Gremme G, Steinbiss S, Kurtz S. 2013. GenomeTools: A Comprehensive Software Library for Efficient Processing of Structured Genome Annotations. IEEE/ACM Transactions on Computational Biology and Bioinformatics. 10:645–656. doi: 10.1109/TCBB.2013.68.

Guigó R. 1998. Assembling Genes from Predicted Exons in Linear Time with Dynamic Programming. Journal of Computational Biology. 5:681–702. doi: 10.1089/cmb.1998.5.681.

Gutiérrez RJ, Franklin AB, Lahaye WS. 1995. Spotted Owl (*Strix occidentalis*). The Birds of North America Online (A. Poole, Ed.). Ithaca: Cornell Lab of Ornithology. [Accessed 2016 Oct 1]. Retrieved from the Birds of North America Online: https://birdsna.org/Species-Account/bna/species/spoowl. doi: 10.2173/bna.179

Hamer TE, Forsman ED, Fuchs AD, Walters ML. 1994. Hybridization between Barred and Spotted Owls. The Auk. 111:487–492. doi: 10.2307/4088616.

Hanna ZR, Henderson JB. 2017. NSO-genome-scripts. Version 1.0.0. Zenodo. doi: 10.5281/zenodo.805012.

Henderson JB, Hanna ZR. 2016a. dupchk. Version 1.0.0. Zenodo. doi: 10.5281/zenodo.163722.

Henderson JB, Hanna ZR. 2016b. GItaxidIsVert. Version 1.0.0. Zenodo. doi: 10.5281/zenodo.163737.

Henderson JB, Hanna ZR. 2016c. ScaffSplitN50s. Version 1.0.0. Zenodo. doi: 10.5281/zenodo.163683.

Henderson JB, Hanna ZR. 2016d. scafN50. Version 1.0.0. Zenodo. doi: 10.5281/zenodo.163739.

Henderson JB, Hanna ZR. 2016e. scafSeqContigInfo. Version 1.0.0. Zenodo. doi: 10.5281/zenodo.163748.

Hoffman W, Martin K. 2003. The CMake Build Manager. Dr. Dobb's. [Accessed 2016 Jun 17]. http://www.drdobbs.com/cpp/the-cmake-build-manager/184405251

Hudson RR, Slatkin M, Maddison WP. 1992. Estimation of Levels of Gene Flow from DNA Sequence Data. Genetics. 132:583–589.

Hunter JD. 2007. Matplotlib: A 2D graphics environment. Computing In Science & Engineering. 9:90–95.

Ihnat DM, MacKenzie D, Meyering J. 2013. cut (GNU coreutils). Version 8.21. [Accessed 2016 Oct 1]. Available from: http://www.gnu.org/software/coreutils/coreutils.html

Jones P et al. 2014. InterProScan 5: genome-scale protein function classification. Bioinformatics. 30(9):1236-1240. doi: 10.1093/bioinformatics/btu031.

Jurka J. 2000. Repbase Update: a database and an electronic journal of repetitive elements. Trends in Genetics. 16:418–420. doi: 10.1016/S0168-9525(00)02093-X.

Jurka J et al. 2005. Repbase Update, a database of eukaryotic repetitive elements. Cytogenetic and Genome Research. 110:462–467. doi: 10.1159/000084979.

Jurka J. 1998. Repeats in genomic DNA: mining and meaning. Current Opinion in Structural Biology. 8:333–337. doi: 10.1016/S0959-440X(98)80067-5.

Kearse M et al. 2012. Geneious Basic: An integrated and extendable desktop software platform for the organization and analysis of sequence data. Bioinformatics. 28:1647–1649. doi: 10.1093/bioinformatics/bts199.

Keller O, Kollmar M, Stanke M, Waack S. 2011. A novel hybrid gene prediction method employing protein multiple sequence alignments. Bioinformatics. 27(6):757-763. doi: 10.1093/bioinformatics/btr010.

Kitware. 2015. CMake. Version 3.2.3. [Accessed 2016 Oct 1]. Available from: https://cmake.org

Korf I. 2004. Gene finding in novel genomes. BMC Bioinformatics. 5:1–9. doi: 10.1186/1471-2105-5-59.

Leinonen R, Sugawara H, Shumway M. 2011. The Sequence Read Archive. Nucl. Acids Res. 39:D19–D21. doi: 10.1093/nar/gkq1019.

Li H. 2013a. Aligning sequence reads, clone sequences and assembly contigs with BWA-MEM. arXiv:1303.3997 Q-Bio. [Accessed 2016 Feb 16]. Available from http://arxiv.org/abs/1303.3997

Li H. 2013b. bioawk. Version 1.0. [Accessed 2016 Oct 1]. Available from: https://github.com/lh3/bioawk

Li H. 2015. PSMC. Version 0.6.5-r67. [Accessed 2016 Oct 1]. Available from: https://github.com/lh3/psmc

Li H et al. 2009. The Sequence Alignment/Map format and SAMtools. Bioinformatics. 25:2078–2079. doi: 10.1093/bioinformatics/btp352.

Li H, Durbin R. 2011. Inference of human population history from individual whole-genome sequences. Nature. 475:493–496. doi: 10.1038/nature10231.

Li H, Handsaker B, Danecek P, McCarthy S, Marshall J. 2016. bcftools. Version 1.3.1. [Accessed 2016 Oct 1]. Available from: https://github.com/samtools/bcftools

Li H, Handsaker B, Marshall J, Danecek P. 2016. Samtools. Version 1.3.1 with HTSlib 1.3.1. [Accessed 2016 Oct 1]. Available from: http://www.htslib.org

Luo R et al. 2012. SOAPdenovo2: an empirically improved memory-efficient short-read de novo assembler. GigaScience. 1:18. doi: 10.1186/2047-217X-1-18.

MacKenzie D. 2013. fold (GNU coreutils). Version 8.21. [Accessed 2016 Oct 1]. Available from: http://www.gnu.org/software/coreutils/coreutils.html

Matplotlib Development Team. 2016. matplotlib. Version 1.5.1. [Accessed 2016 Oct 1]. Available from: http://matplotlib.org

Mazur KM, James PC. 2000. Barred Owl (*Strix varia*). *The Birds of North America Online* (A. Poole, Ed.) Ithaca: Cornell Lab of Ornithology. [Accessed 2016 Oct 1]. Retrieved from the Birds of North America Online: https://birdsna.org/Species-Account/bna/species/brdowl. doi: 10.2173/bna.508

McKenna A et al. 2010. The Genome Analysis Toolkit: A MapReduce framework for analyzing next-generation DNA sequencing data. Genome Res. 20:1297–1303. doi: 10.1101/gr.107524.110.

NCBI Resource Coordinators. 2016. Database resources of the National Center for Biotechnology Information. Nucleic Acids Res. 44:D7-19. doi: 10.1093/nar/gkv1290.

Noon BR, Biles CM. 1990. Mathematical Demography of Spotted Owls in the Pacific Northwest. The Journal of Wildlife Management. 54:18–27. doi: 10.2307/3808895.

NumPy Developers. 2016. NumPy. Version 1.11.1. [Accessed 2016 Oct 1]. Available from: http://www.numpy.org

O'Connell J et al. 2015. NxTrim: optimized trimming of Illumina mate pair reads. Bioinformatics. 31(12):2035-2037. doi: 10.1093/bioinformatics/btv057.

O'Connell J. 2014. NxTrim. Version 0.2.3-alpha. [Accessed 2016 Oct 1]. Available from: https://github.com/sequencing/NxTrim

Parra G, Bradnam K, Korf I. 2007. CEGMA: a pipeline to accurately annotate core genes in eukaryotic genomes. Bioinformatics. 23:1061–1067. doi: 10.1093/bioinformatics/btm071.

Price AL, Jones NC, Pevzner PA. 2005. De novo identification of repeat families in large genomes. Bioinformatics. 21:i351–i358. doi: 10.1093/bioinformatics/bti1018.

Python Software Foundation. 2016. Python. Version 2.7.12. [Accessed 2016 Oct 1]. Available from: https://www.python.org

Simão FA, Waterhouse RM, Ioannidis P, Kriventseva EV, Zdobnov EM. 2015a. BUSCO: assessing genome assembly and annotation completeness with single-copy orthologs. Bioinformatics. 31(19):3210-3212. doi: 10.1093/bioinformatics/btv351.

Simão FA, Waterhouse RM, Ioannidis P, Kriventseva EV, Zdobnov EM. 2015b. BUSCO. Version 1.1b1. [Accessed 2016 Oct 1]. Available from: http://busco.ezlab.org

Simpson JT. 2014. Exploring genome characteristics and sequence quality without a reference. Bioinformatics. 30:1228–1235. doi: 10.1093/bioinformatics/btu023.

Simpson JT, Durbin R. 2010. Efficient construction of an assembly string graph using the FM-index. Bioinformatics. 26:i367–i373. doi: 10.1093/bioinformatics/btq217.

Simpson JT, Durbin R. 2016. SGA - String Graph Assembler. Version 0.10.14. [Accessed 2016 Oct 1]. Available from: https://github.com/jts/sga

Slater GSC, Birney E. 2005. Automated generation of heuristics for biological sequence comparison. BMC Bioinformatics. 6:1–11. doi: 10.1186/1471-2105-6-31.

Smeds L, Qvarnström A, Ellegren H. 2016. Direct estimate of the rate of germline mutation in a bird. Genome Res. 26:1211–1218. doi: 10.1101/gr.204669.116.

Smit A, Hubley R, Green P. 2013. RepeatMasker Open-4.0. [Accessed 2016 Oct 1]. Available from: http://www.repeatmasker.org

Smit A, Hubley R, National Center for Biotechnology Information. 2015. RMBlast. [Accessed 2016 Oct 1]. Available from: http://www.repeatmasker.org/RMBlast.html

Smit AFA, Hubley R. 2015. RepeatModeler Open-1.0. [Accessed 2016 Oct 1]. Available from: http://www.repeatmasker.org

Stanke M. 2015. AUGUSTUS. Version 3.2.1. [Accessed 2016 Oct 1]. Available from: http://bioinf.uni-greifswald.de/augustus

USDA Forest Service. 1992. Final Environmental Impact Statement on Management for the Northern Spotted Owl in the National Forests. USDA Forest Service, National Forest System: Portland, Oregon. 2 vol.

Van der Auwera GA et al. 2013. From FastQ data to high confidence variant calls: the Genome Analysis Toolkit best practices pipeline. Curr Protoc Bioinformatics. 11:11.10.1-11.10.33. doi: 10.1002/0471250953.bi1110s43.

Vinogradov AE. 2005. Genome size and chromatin condensation in vertebrates. Chromosoma. 113:362–369. doi: 10.1007/s00412-004-0323-3.

Wheeler TJ et al. 2013. Dfam: a database of repetitive DNA based on profile hidden Markov models. Nucl. Acids Res. 41:D70–D82. doi: 10.1093/nar/gks1265.

Wu Y et al. 2016. Retinal transcriptome sequencing sheds light on the adaptation to nocturnal and diurnal lifestyles in raptors. Scientific Reports. 6:33578. doi: 10.1038/srep33578.

Yates A et al. 2016. Ensembl 2016. Nucl. Acids Res. 44:D710–D716. doi: 10.1093/nar/gkv1157.

Zhang Z, Schwartz S, Wagner L, Miller W. 2000. A Greedy Algorithm for Aligning DNA Sequences. Journal of Computational Biology. 7:203–214. doi: 10.1089/10665270050081478.
